# Supplementary material for: An improved termite life cycle optimizer algorithm for global function optimization
Source: PeerJ Comput Sci. 2025 Feb 17;11:e2671. doi: 10.7717/peerj-cs.2671 (PMC11888856; doi:10.7717/peerj-cs.2671)
Supplement: Supplemental Information 3 — The original source of CEC2013 is divided into two parts: the first part is the functional evaluation criteria and details of the CEC2013 test set, which can be found in Appendix CEC2013. The second part is the relevant data files needed to test the CEC2013 test set, which can be found in the code files (e.g., input_data, cec13, etc.,). [file peerj-cs-11-2671-s003.pdf]

ResearchGate

See discussions, stats, and author profiles for this publication at: https://www.researchgate.net/publication/256995189

Problem Definitions and Evaluation Criteria for the CEC 2013 Special Session on Real-Parameter Optimization

Article · January 2013

CITATIONS
823

READS
9,600

4 authors, including:

Jing Liang
Zhengzhou University
119 PUBLICATIONS **13,897** CITATIONS

SEE PROFILE

Ponnuthurai N. Suganthan
Nanyang Technological University
563 PUBLICATIONS **50,573** CITATIONS

SEE PROFILE

Alfredo García Hernández-Díaz
Universidad Pablo de Olavide
67 PUBLICATIONS **2,219** CITATIONS

SEE PROFILE

Some of the authors of this publication are also working on these related projects:

Project Survey on multi-objective evolutionary algorithms for the solution of the environmental/economic dispatch problems View project

Project 2019 CEC-31 Special Session on Evolutionary Computation for Smart City View project

All content following this page was uploaded by Alfredo García Hernández-Díaz on 22 May 2014.

The user has requested enhancement of the downloaded file.

Problem Definitions and Evaluation Criteria for the CEC 2013 Special Session on Real-Parameter Optimization

J. J. Liang[1], B. Y. Qu[2], P. N. Suganthan[3], Alfredo G. Hernández-Díaz[4]

[1] School of Electrical Engineering, Zhengzhou University, Zhengzhou, China
[2] School of Electric and Information Engineering, Zhongyuan University of Technology, Zhengzhou, China
[3] School of EEE, Nanyang Technological University, Singapore
[4] Department of Economics, Quantitative Methods and Economic History, Pablo de Olavide University, Seville, Spain

liangjing@zzu.edu.cn, e070088@e.ntu.edu.sg, epnsugan@e.ntu.edu.sg, agarher@upo.es

Technical Report 201212, Computational Intelligence Laboratory, Zhengzhou University, Zhengzhou China
And
Technical Report, Nanyang Technological University, Singapore

January 2013

Single objective optimization algorithms are the basis of the more complex optimization algorithms such as multi-objective optimizations algorithms, niching algorithms, constrained optimization algorithms and so on. Research on the single objective optimization algorithms influence the development of these optimization branches mentioned above. In the recent years various kinds of novel optimization algorithms have been proposed to solve real-parameter optimization problems. Eight years have passed since the CEC'05 Special Session on Real-Parameter Optimization[1]. Considering the comments on the CEC'05 test suite received by us, we propose to organize a new competition on real parameter single objective optimization. In the CEC'13 test suite, the previously proposed composition functions[2] are improved and additional test functions are included.

This special session is devoted to the approaches, algorithms and techniques for solving real parameter single objective optimization without making use of the exact equations of the test functions. We encourage all researchers to test their algorithms on the CEC'13 test suite which includes 28 benchmark functions. The participants are required to send the final results in the format specified in the technical report to the organizers. The organizers will present an overall analysis and comparison based on these results. We will also use statistical tests on convergence performance to compare algorithms that eventually generate similar final solutions. Papers on novel concepts that help us in understanding problem characteristics are also welcome.

The C and Matlab codes for CEC'13 test suite can be downloaded from the website given below:

http://www.ntu.edu.sg/home/EPNSugan/index_files/CEC2013/CEC2013.htm

1. Introduction to the 28 CEC'13 Test Functions

1.1 Some Definitions:

All test functions are minimization problems defined as following:

$$\text{Min } f(x), x = [x_1, x_2, ..., x_D]^{\mathrm{T}}$$

D: dimensions.

$o = [o_1, o_2, ..., o_D]^{\mathrm{T}}$: the shifted global optimum (defined in "shift_data.txt"), which is randomly distributed in $[-80,80]^{D}$.

All test functions are shifted to *o* and scalable.

For convenience, the same search ranges are defined for all test functions.

Search range: $[-100,100]^{D}$.

M_1, M_2, … , M_{10}: orthogonal (rotation) matrix generated from standard normally distributed entries by Gram-Schmidt orthonormalization. (Matrix data is saved in "M_D2.txt", "M_D10.txt", "M_D30.txt", "M_D50.txt", "M_D100.txt". Each file concludes ten *D*D* orthogonal matrices.)

Λ^{α} : a diagonal matrix in D dimensions with the ith diagonal element as $\lambda_{ii} = \alpha^{\frac{i-1}{2(D-1)}}$, $i=1,2,\ldots,D$.

T_{asy}^{β} : if $x_i > 0, x_i = x_i^{1+\beta\frac{i-1}{D-1}\sqrt{x_i}}$, for $i = 1,...,D$ [3]

T_{osz}: for $x_i = \text{sign}(x_i)\exp(\hat{x}_i + 0.049(\sin(c_1\hat{x}_i) + \sin(c_2\hat{x}_i)))$, for $i = 1$ and D [3]

$$\text{where } \hat{x}_i = \begin{cases} \log(|x_i|) & \text{if } x_i \neq 0 \\ 0 & \text{otherwise} \end{cases}, \text{sign}(x_i) = \begin{cases} -1 & \text{if } x_i < 0 \\ 0 & \text{if } x_i = 0 \\ 1 & \text{otherwise} \end{cases}$$

$$c_1 = \begin{cases} 10 & \text{if } x_i > 0 \\ 5.5 & \text{otherwise} \end{cases}, \text{ and } c_2 = \begin{cases} 7.9 & \text{if } x_i > 0 \\ 3.1 & \text{otherwise} \end{cases}$$

1.2 Summary of the 28 CEC'13 Test Functions

Table I. Summary of the 28 CEC'13 Test Functions

	No.	Functions	$f_i*=f_i(x*)$
Unimodal Functions	1	Sphere Function	-1400
	2	Rotated High Conditioned Elliptic Function	-1300
	3	Rotated Bent Cigar Function	-1200
	4	Rotated Discus Function	-1100
	5	Different Powers Function	-1000
Basic Multimodal Functions	6	Rotated Rosenbrock's Function	-900
	7	Rotated Schaffers F7 Function	-800
	8	Rotated Ackley's Function	-700
	9	Rotated Weierstrass Function	-600
	10	Rotated Griewank's Function	-500
	11	Rastrigin's Function	-400
	12	Rotated Rastrigin's Function	-300
	13	Non-Continuous Rotated Rastrigin's Function	-200
	14	Schwefel's Function	-100
	15	Rotated Schwefel's Function	100
	16	Rotated Katsuura Function	200
	17	Lunacek Bi_Rastrigin Function	300
	18	Rotated Lunacek Bi_Rastrigin Function	400
	19	Expanded Griewank's plus Rosenbrock's Function	500
	20	Expanded Scaffer's F6 Function	600
Composition Functions	21	Composition Function 1 (n=5,Rotated)	700
	22	Composition Function 2 (n=3,Unrotated)	800
	23	Composition Function 3 (n=3,Rotated)	900
	24	Composition Function 4 (n=3,Rotated)	1000
	25	Composition Function 5 (n=3,Rotated)	1100
	26	Composition Function 6 (n=5,Rotated)	1200
	27	Composition Function 7 (n=5,Rotated)	1300
	28	Composition Function 8 (n=5,Rotated)	1400
Search Range: $[-100,100]^D$			

***Please Notice: These problems should be treated as black-box problems. The explicit equations of the problems are not allowed to be used. However, the dimensionality of the problems and the total number of available function evaluations can be considered as known values which can be used to design dynamic or adaptive approaches in your algorithm.**

1.3 Definitions of the 28 CEC'13 Test Functions

A. Unimodal Functions:

1) Sphere Function

$$f_1(x) = \sum_{i=1}^{D} z_i^{\,2} + f_1{*}, \mathbf{z} = \mathbf{x} - \mathbf{o} \qquad (1)$$

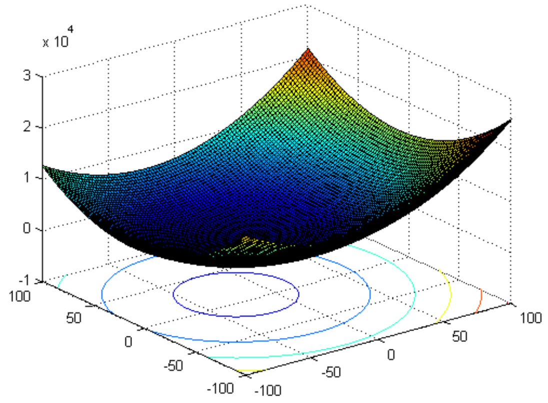

Figure 1. 3-*D* map for 2-*D* function

Properties:

- ➢ Unimodal
- ➢ Separable

2) Rotated High Conditioned Elliptic Function

$$f_2(x) = \sum_{i=1}^{D} (10^6)^{\frac{i-1}{D-1}} z_i^2 + f_2{*}, \quad z = T_{osz}(\mathbf{M}_1(x-o)) \qquad (2)$$

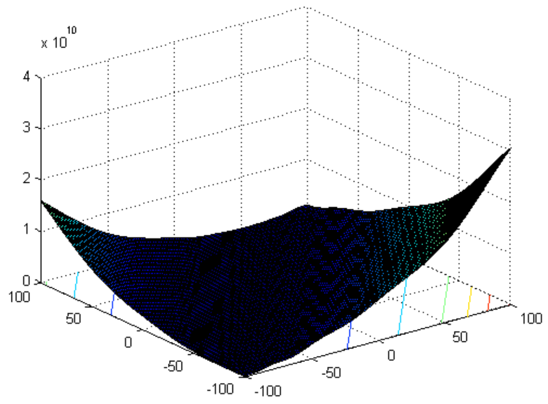

Figure 2. 3-*D* map for 2-*D* function

Properties:

- ➢ Unimodal
- ➢ Non-separable

- Quadratic ill-conditioned
- Smooth local irregularities

3) Rotated Bent Cigar Function

$$f_3(x) = z_1^2 + 10^6 \sum_{i=2}^{D} z_i^2 + f_3{*}, \quad z = \mathbf{M}_2 T_{asy}^{0.5}(\mathbf{M}_1(x - o)) \qquad (4)$$

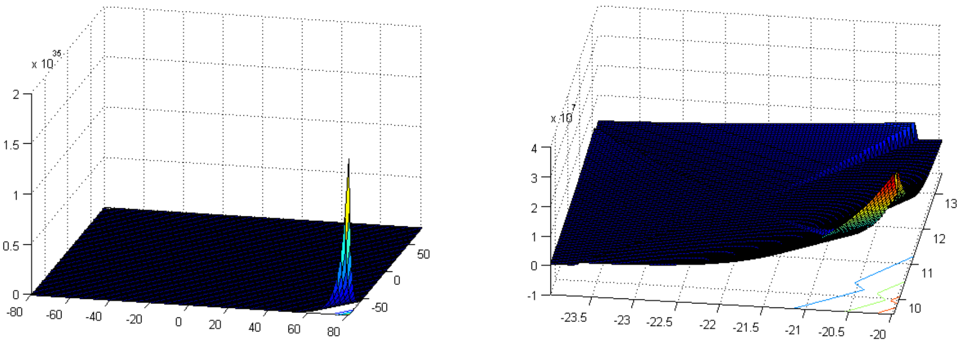

Figure 3. 3-*D* map for 2-*D* function

Properties:

- Unimodal
- Non-separable
- Smooth but narrow ridge

4) Rotated Discus Function

$$f_4(x) = 10^6 z_1^2 + \sum_{i=2}^{D} z_i^2 + f_4{*}, \quad z = T_{osz}(\mathbf{M}_1(x - o)) \qquad (3)$$

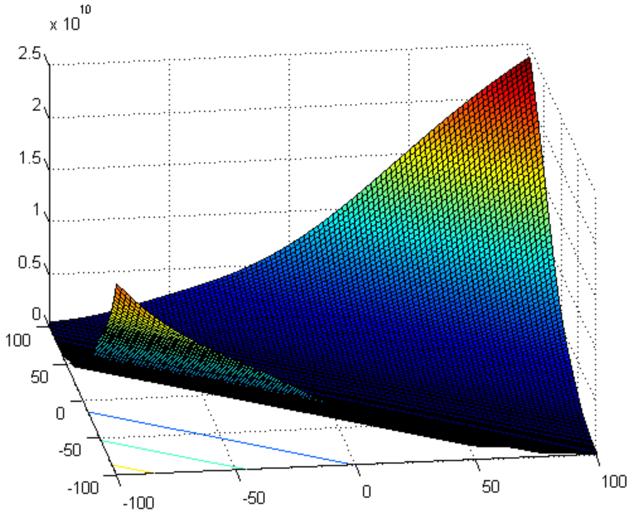

Figure 4. 3-*D* map for 2-*D* function

Properties:

- Unimodal
- Non-separable
- Asymmetrical
- Smooth local irregularities
- With one sensitive direction

5) Different Powers Function

$$f_5(x) = \sqrt{\sum_{i=1}^{D} |z_i|^{2+4\frac{i-1}{D-1}}} + f_5{*}, \quad z = x - o \qquad (5)$$

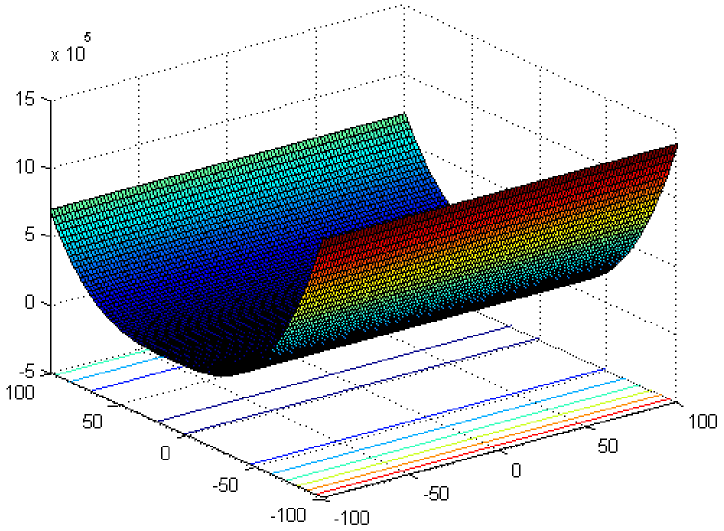

Figure 5. 3-*D* map for 2-*D* function

Properties:

- Unimodal
- Separable
- Sensitivities of the z_i-variables are different.

B. Basic Multimodal Functions

6) Rotated Rosenbrock's Function

$$f_6(x) = \sum_{i=1}^{D-1} (100(z_i^2 - z_{i+1})^2 + (z_i - 1)^2) + f_6{*}, \quad z = \mathbf{M}_1(\frac{2.048(x-o)}{100}) + 1 \qquad (6)$$

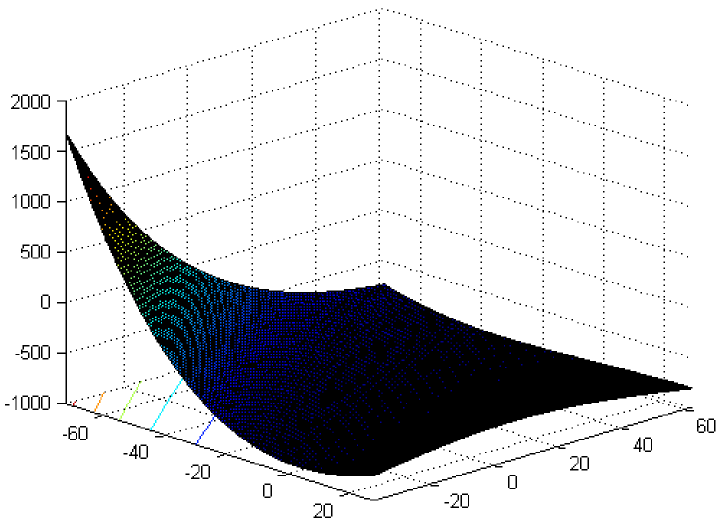

Figure 6(a). 3-*D* map for 2-*D* function

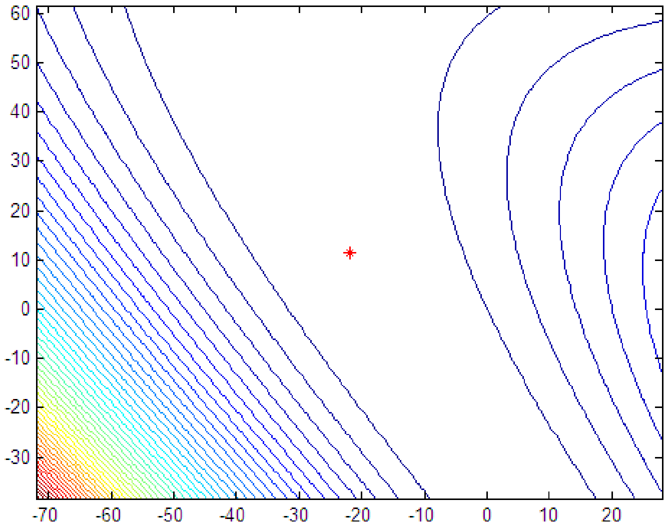

Figure 6(b).Contour map for 2-*D* function

Properties:

- Multi-modal
- Non-separable
- Having a very narrow valley from local optimum to global optimum

7) Rotated Schaffers F7 Function

$$f_7(\mathbf{x}) = \left(\frac{1}{D-1}\sum_{i=1}^{D-1}\left(\sqrt{z_i} + \sqrt{z_i}\sin^2(50 z_i^{0.2})\right)\right)^2 + f_7{}^* \qquad (7)$$

$$z_i = \sqrt{y_i^2 + y_{i+1}^2} \text{ for } i = 1,\ldots,D,\ y = \Lambda^{10}\mathbf{M}_2 T_{asy}^{0.5}(\mathbf{M}_1(x-o))$$

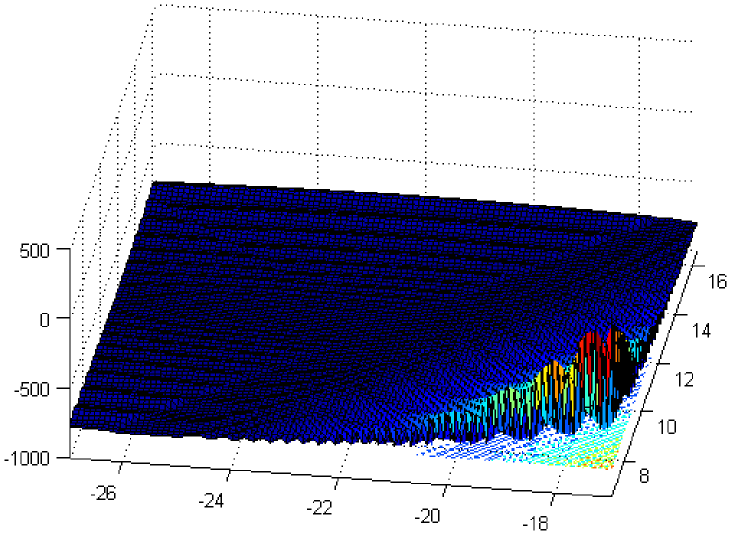

Figure 7(a). 3-*D* map for 2-*D* function

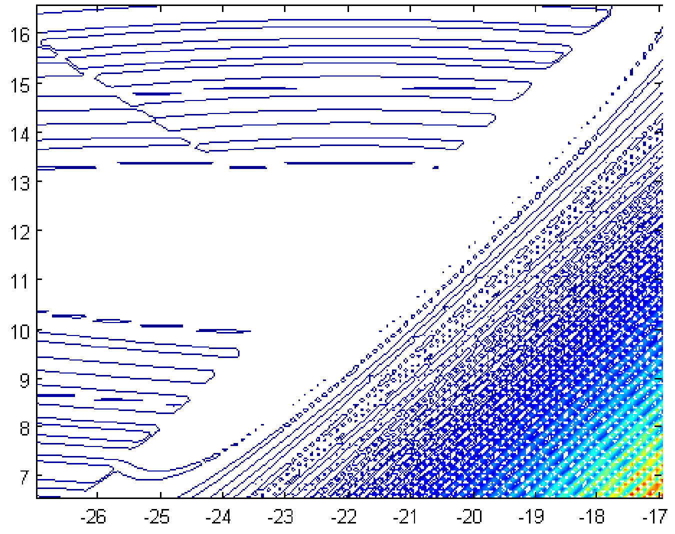

Figure 7(b).Contour map for 2-*D* function

Properties:

- Multi-modal
- Non-separable
- Asymmetrical
- Local optima's number is huge

8) Rotated Ackley's Function

$$f_8(x) = -20\exp(-0.2\sqrt{\frac{1}{D}\sum_{i=1}^{D} z_i^2}) - \exp(\frac{1}{D}\sum_{i=1}^{D}\cos(2\pi z_i)) + 20 + e + f_8{}^* \qquad (8)$$

$$z = \Lambda^{10}\mathbf{M}_2 T_{asy}^{0.5}(\mathbf{M}_1(x-o))$$

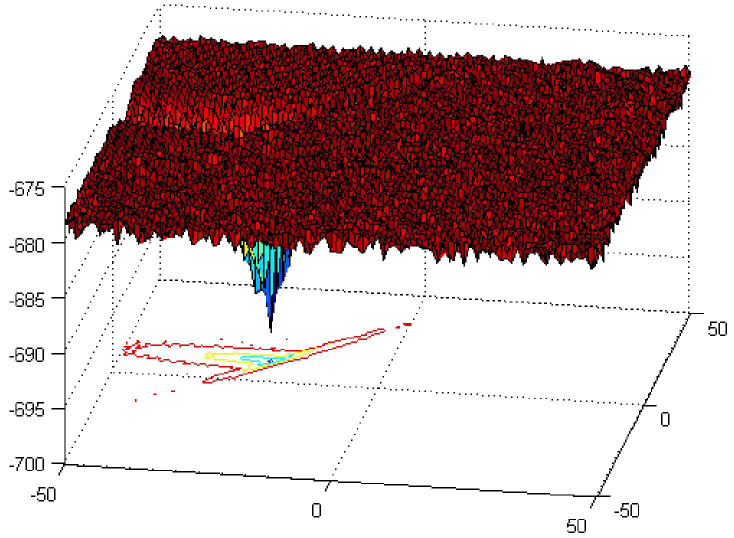

Figure 8(a). 3-*D* map for 2-*D* function

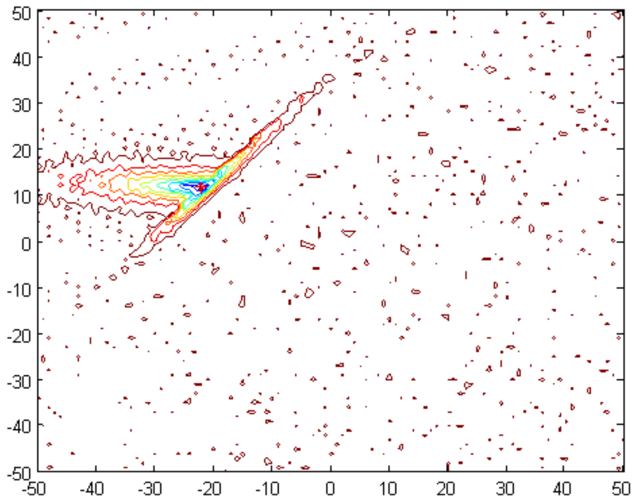

Figure 8(b).Contour map for 2-*D* function

Properties:

- Multi-modal
- Non-separable
- Asymmetrical

9) Rotated Weierstrass Function

$$f_9(x)=\sum_{i=1}^{D}\left(\sum_{k=0}^{k\max}[a^k\cos(2\pi b^k(z_i+0.5))]\right)-D\sum_{k=0}^{k\max}[a^k\cos(2\pi b^k\cdot 0.5)]+f_9^* \qquad (9)$$

a=0.5, b=3, kmax=20, $z=\Lambda^{10}\mathbf{M}_2T_{asy}^{0.5}(\mathbf{M}_1\frac{0.5(x-o)}{100})$

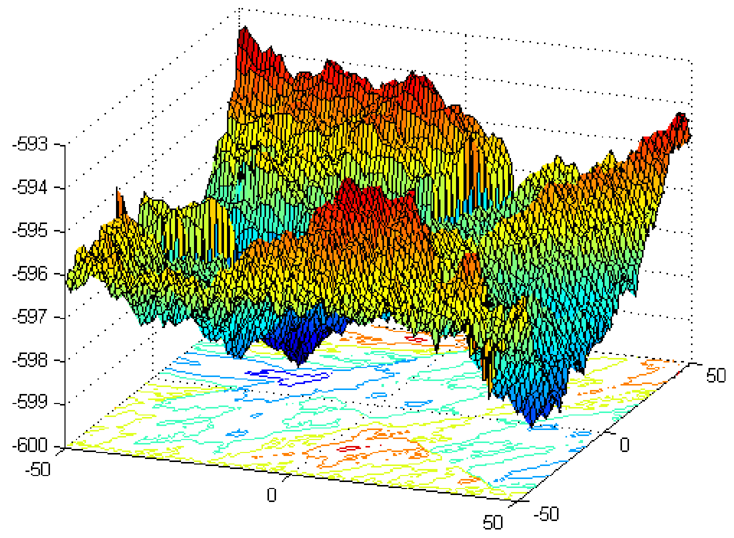

Figure 9(a). 3-*D* map for 2-*D* function

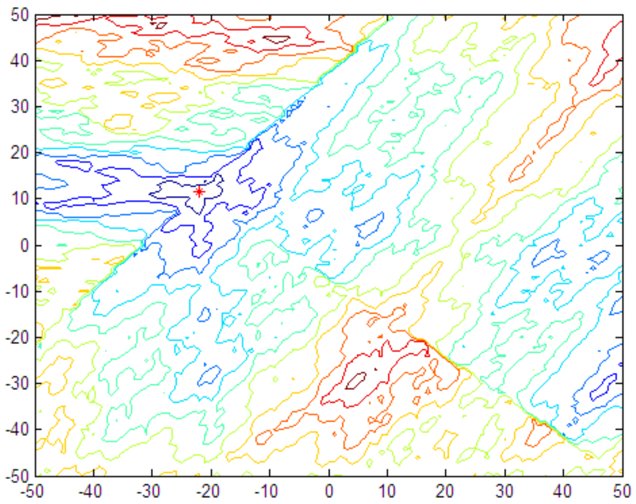

Figure 9(b).Contour map for 2-*D* function

Properties:

- Multi-modal
- Non-separable
- Asymmetrical
- Continuous but differentiable only on a set of points

10) Rotated Griewank's Function

$$f_{10}(x) = \sum_{i=1}^{D} \frac{z_i^{\,2}}{4000} - \prod_{i=1}^{D} \cos\left(\frac{z_i}{\sqrt{i}}\right) + 1 + f_{10}^{\,*} \quad , \quad z = \Lambda^{100} \mathbf{M}_1 \frac{600(x-o)}{100} \qquad (10)$$

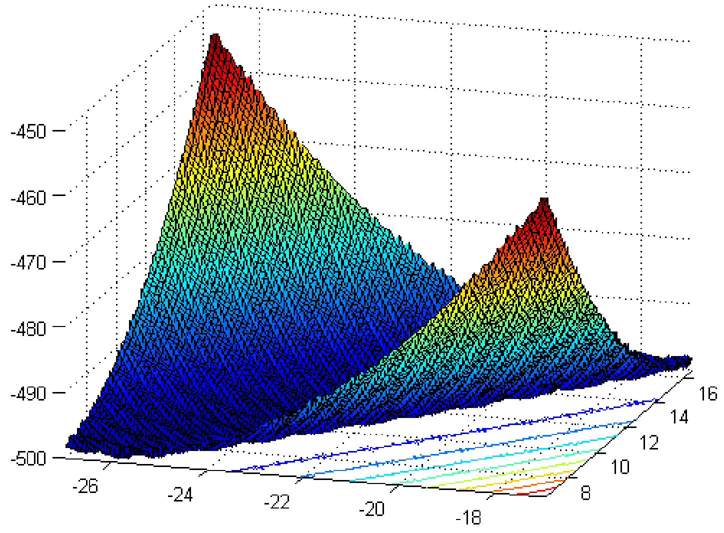

Figure 10(a). 3-*D* map for 2-*D* function

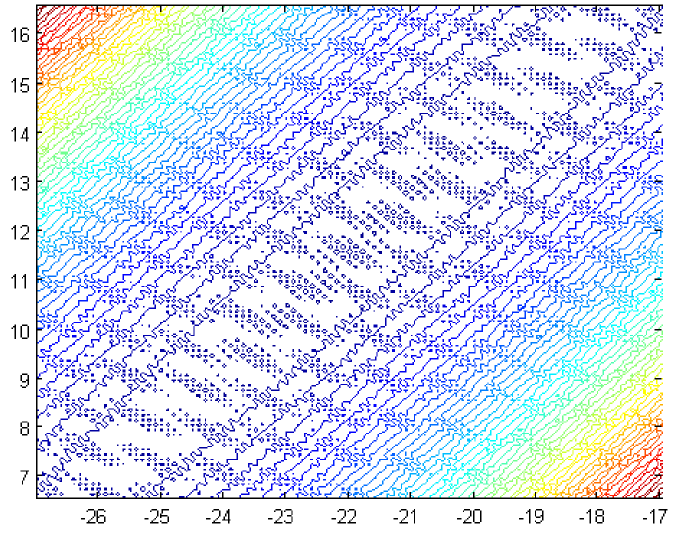

Figure 10(b).Contour map for 2-*D* function

Properties:

- Multi-modal
- Rotated
- Non-separable

11) Rastrigin's Function

$$f_{11}(x) = \sum_{i=1}^{D} (z_i^{\ 2} - 10\cos(2\pi z_i) + 10) + f_{11}^{\ *} \qquad (11)$$

$$z = \Lambda^{10} T_{asy}^{0.2} (T_{osz} (\frac{5.12(x-o)}{100}))$$

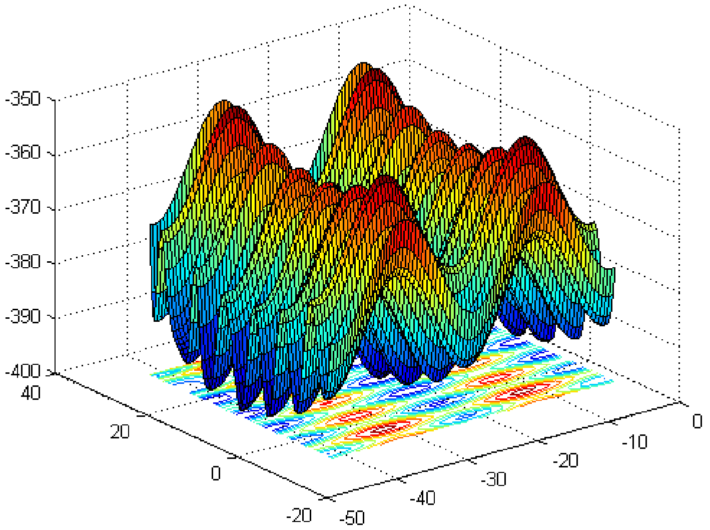

Figure 11(a). 3-*D* map for 2-*D* function

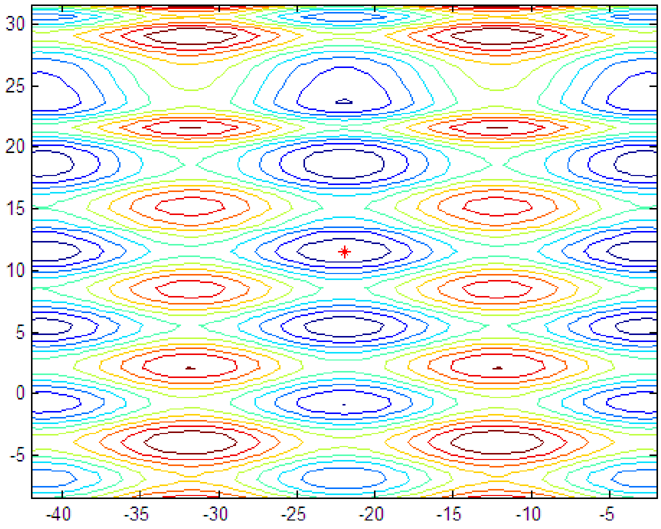

Figure 11(b).Contour map for 2-*D* function

Properties:

- Multi-modal
- Separable
- Asymmetrical
- Local optima's number is huge

12) Rotated Rastrigin's Function

$$f_{12}(x) = \sum_{i=1}^{D} (z_i^2 - 10\cos(2\pi z_i) + 10) + f_{12}^* \qquad (12)$$

$$z = \mathbf{M}_1 \Lambda^{10} \mathbf{M}_2 T_{asy}^{0.2}(T_{osz}(\mathbf{M}_1 \frac{5.12(x-o)}{100}))$$

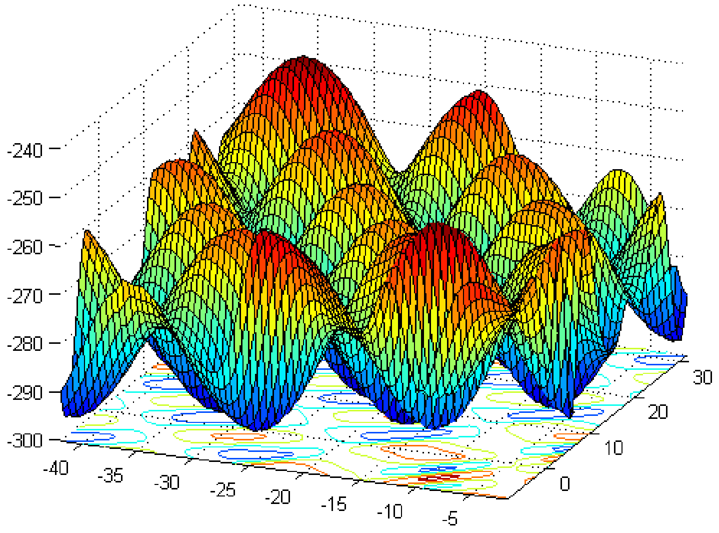

Figure 11(a). 3-*D* map for 2-*D* function

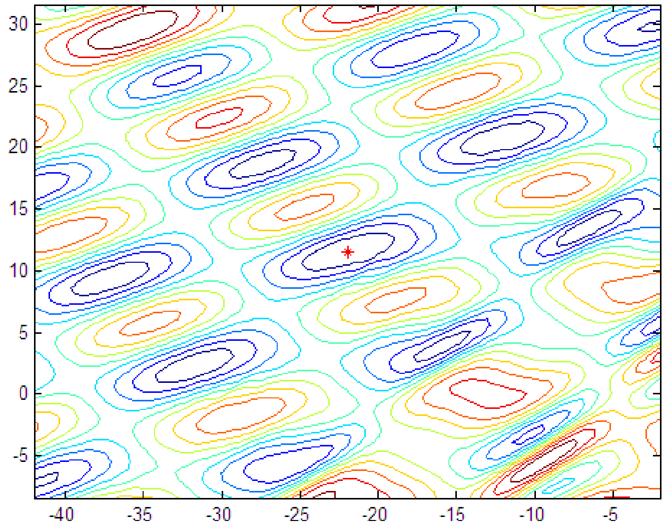

Figure 11(b).Contour map for 2-*D* function

Properties:

- Multi-modal
- Non-separable
- Asymmetrical
- Local optima’s number is huge

13) Non-continuous Rotated Rastrigin's Function

$$f_{13}(x)=\sum_{i=1}^{D}(z_i^2-10\cos(2\pi z_i)+10)+f_{13}* \qquad (13)$$

$$\hat{x}=\mathbf{M}_1\frac{5.12(x-o)}{100},\ y_i=\begin{cases}\hat{x}_i & \text{if } |\hat{x}_i|\le 0.5\\ round(2\hat{x}_i)/2 & \text{if } |\hat{x}_i|>0.5\end{cases}\ \text{for } i=1,2,...,D$$

$$z=\mathbf{M}_1\Lambda^{10}\mathbf{M}_2T_{asy}^{0.2}(T_{osz}(y))$$

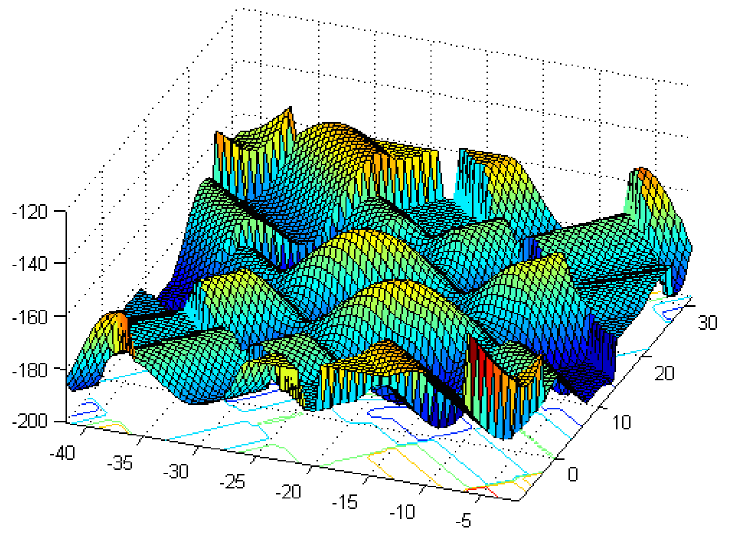

Figure 13(a). 3-*D* map for 2-*D* function

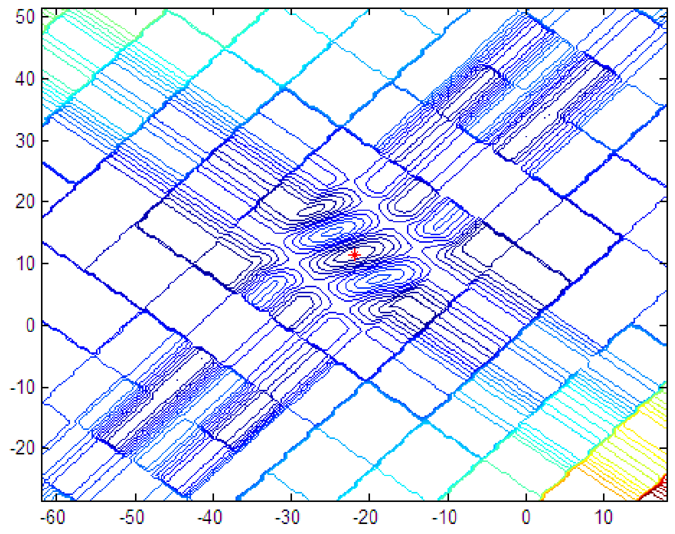

Figure 13(b).Contour map for 2-*D* function

Properties:

- Multi-modal
- Rotated

- Non-separable
- Asymmetrical
- Local optima's number is huge
- Non-continuous

14) Schwefel's Function

$$f_{14}(z) = 418.9829 \times D - \sum_{i=1}^{D} g(z_i) + f_{14}*$$

$$z = \Lambda^{10}(\frac{1000(x-o)}{100}) + 4.209687462275036e+002$$

$$g(z_i) = \begin{cases} z_i \sin(|z_i|^{1/2}) & \text{if } |z_i| \le 500 \\ (500 - \mathrm{mod}(z_i, 500))\sin(\sqrt{|500 - \mathrm{mod}(z_i, 500)|}) - \frac{(z_i - 500)^2}{10000D} & \text{if } z_i > 500 \\ (\mathrm{mod}(|z_i|, 500) - 500)\sin(\sqrt{|\mathrm{mod}(|z_i|, 500) - 500|}) - \frac{(z_i + 500)^2}{10000D} & \text{if } z_i < -500 \end{cases} \tag{14}$$

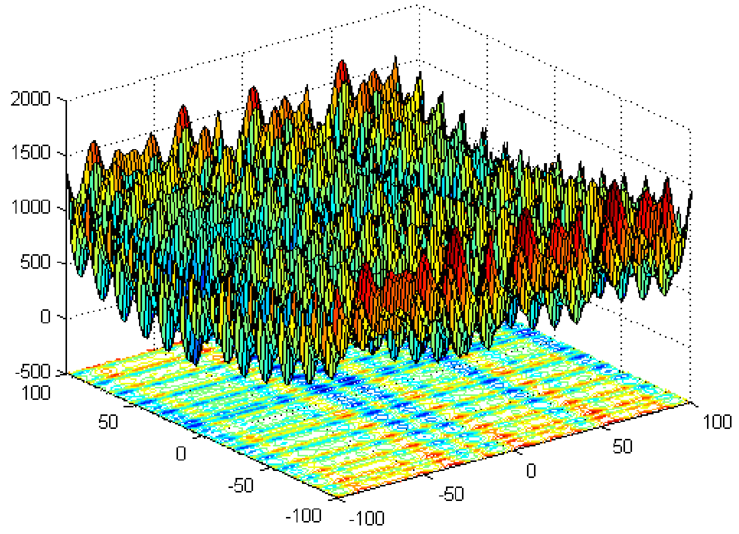

Figure 14(a). 3-*D* map for 2-*D* function

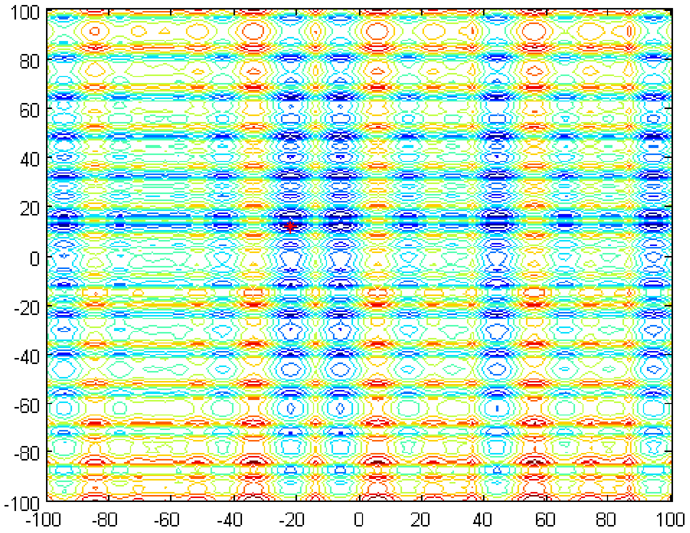

Figure 14(b).Contour map for 2-*D* function

Properties:

- Multi-modal
- Rotated
- Non-separable
- Asymmetrical
- Local optima's number is huge and second better local optimum is far from the global optimum.

15) Rotated Schwefel's Function

$$f_{15}(z) = 418.9829 \times D - \sum_{i=1}^{D} g(z_i) + f_{15}*$$

$$z = \Lambda^{10}\mathbf{M}_1\left(\frac{1000(x-o)}{100}\right)+4.209687462275036\text{e}+002$$

$$g(z_i) = \begin{cases} z_i \sin(|z_i|^{1/2}) & \text{if } |z_i| \le 500 \\ (500 - \text{mod}(z_i, 500))\sin(\sqrt{|500 - \text{mod}(z_i, 500)|}) + \dfrac{(z_i - 500)^2}{10000D} & \text{if } z_i > 500 \\ (\text{mod}(|z_i|, 500) - 500)\sin(\sqrt{|\text{mod}(|z_i|, 500) - 500|}) + \dfrac{(z_i + 500)^2}{10000D} & \text{if } z_i < -500 \end{cases} \quad (15)$$

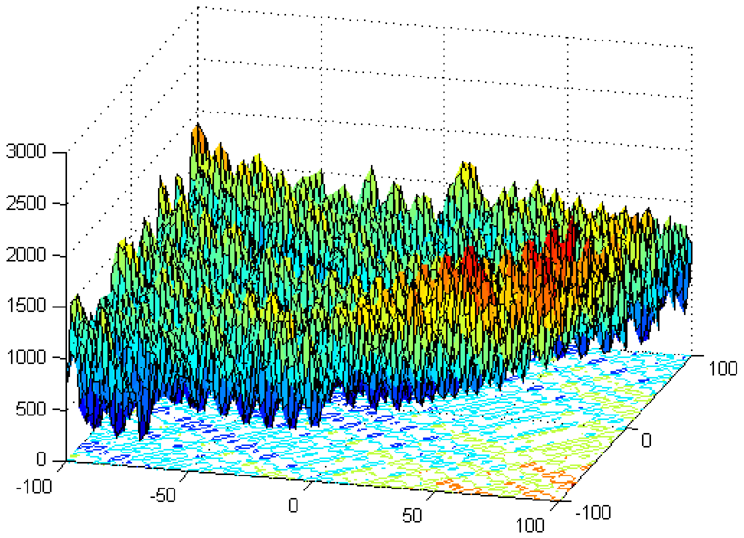

Figure 15(a). 3-*D* map for 2-*D* function

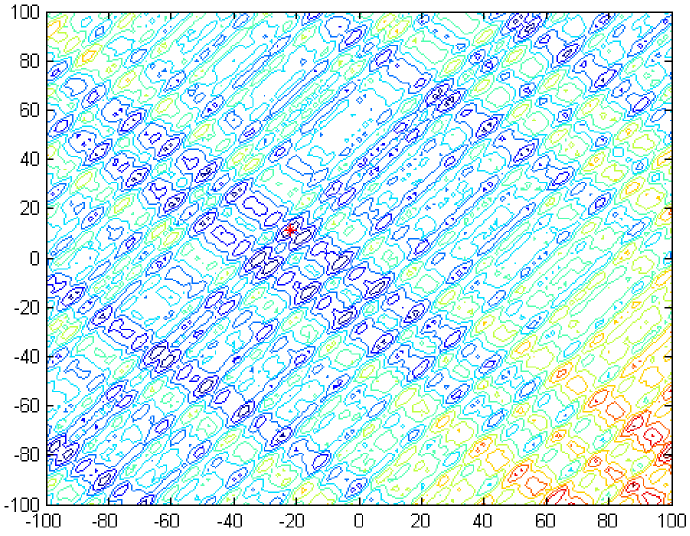

Figure 15(b).Contour map for 2-*D* function

Properties:

- Multi-modal
- Non-separable
- Asymmetrical
- Local optima's number is huge and second better local optimum is far from the global optimum.

16) Rotated Katsuura Function

$$f_{16}(x) = \frac{10}{D^2}\prod_{i=1}^{D}(1+i\sum_{j=1}^{32}\frac{\left|2^j z_i - round(2^j z_i)\right|}{2^j})^{\frac{10}{D^{1.2}}} - \frac{10}{D^2} + f_{16}* \qquad (16)$$

$$z = \mathbf{M}_2 \Lambda^{100} (\mathbf{M}_1 \frac{5(x-o)}{100}))$$

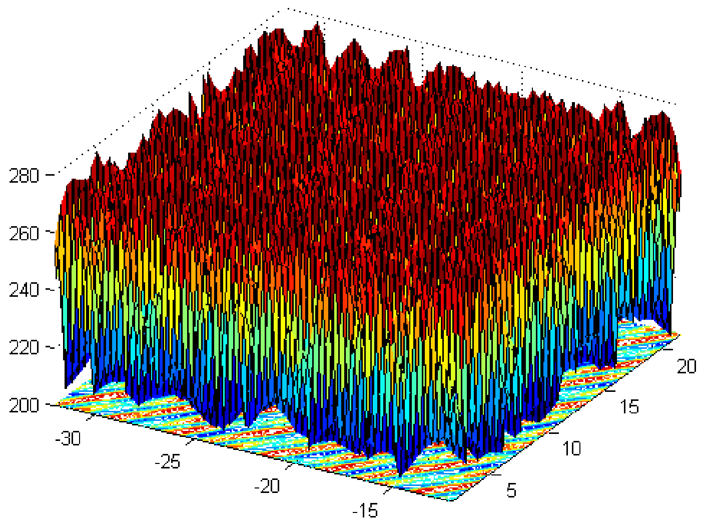

Figure 16(a). 3-*D* map for 2-*D* function

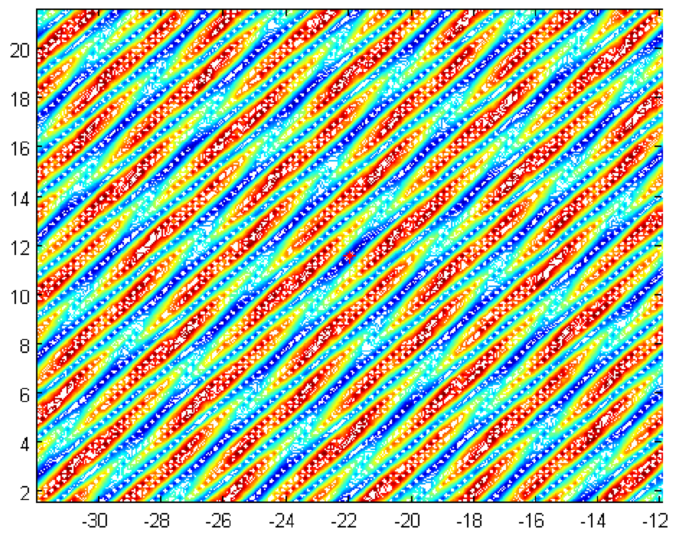

Figure 16(b).Contour map for 2-*D* function

Properties:

- Multi-modal
- Non-separable
- Asymmetrical
- Continuous everywhere yet differentiable nowhere

17) Lunacek bi-Rastrigin Function

$$f_{17}(x)=\min(\sum_{i=1}^{D}(\hat{x}_i-\mu_0)^2, dD+s\sum_{i=1}^{D}(\hat{x}_i-\mu_1)^2)+10(D-\sum_{i=1}^{D}\cos(2\pi\hat{z}_i))+f_{17}* \qquad (17)$$

$$\mu_0=2.5, \mu_1=-\sqrt{\frac{\mu_0^2-d}{s}}, s=1-\frac{1}{2\sqrt{D+20}-8.2}, d=1$$

$$y=\frac{10(x-o)}{100}, \hat{x}_i=2sign(x_i*)y_i+\mu_0, \text{for } i=1,2,...,D$$

$$z=\Lambda^{100}(\hat{x}-\mu_0)$$

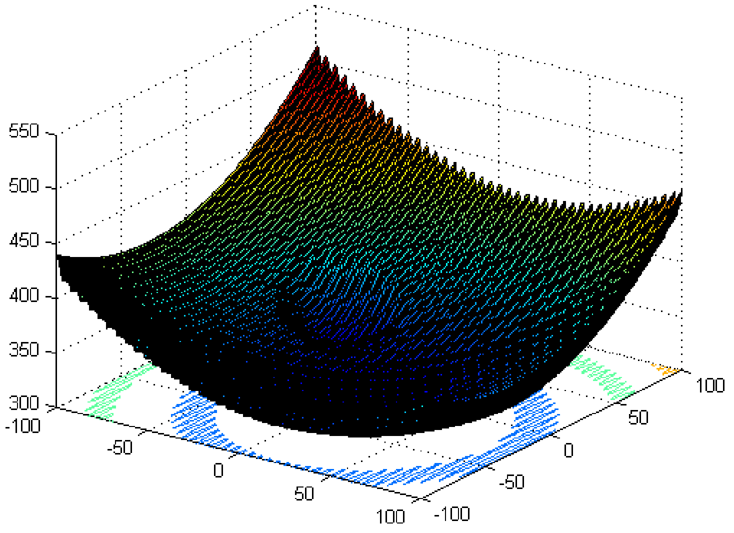

Figure 17(a). 3-*D* map for 2-*D* function

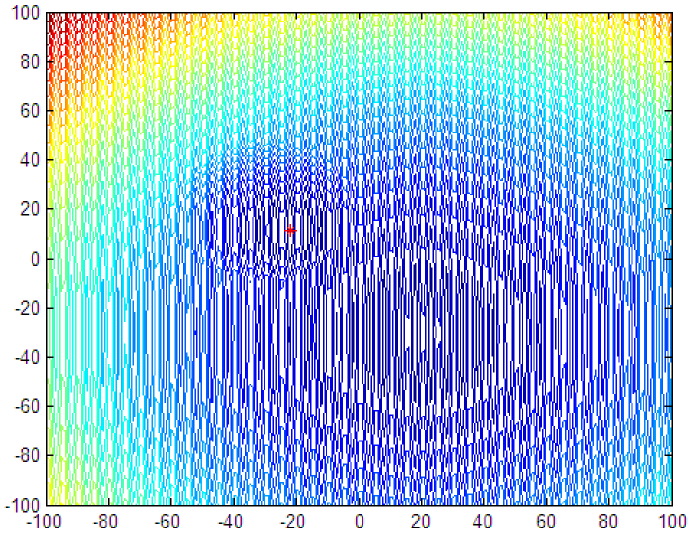

Figure 17(b).Contour map for 2-*D* function

18) Rotated Lunacek bi-Rastrigin Function

$$f_{18}(x) = \min(\sum_{i=1}^{D}(\hat{x}_i - \mu_0)^2, dD + s\sum_{i=1}^{D}(\hat{x}_i - \mu_1)^2) + 10(D - \sum_{i=1}^{D}\cos(2\pi \hat{z}_i)) + f_{18}^* \qquad (18)$$

$$\mu_0 = 2.5, \mu_1 = -\sqrt{\frac{\mu_0^2 - d}{s}}, s = 1 - \frac{1}{2\sqrt{D+20} - 8.2}, d = 1$$

$$y = \frac{10(x-o)}{100}, \hat{x}_i = 2sign(y_i^*)y_i + \mu_0, \text{for } i = 1, 2, ..., D, z = \mathbf{M}_2\Lambda^{100}(\mathbf{M}_1(\hat{x} - \mu_0))$$

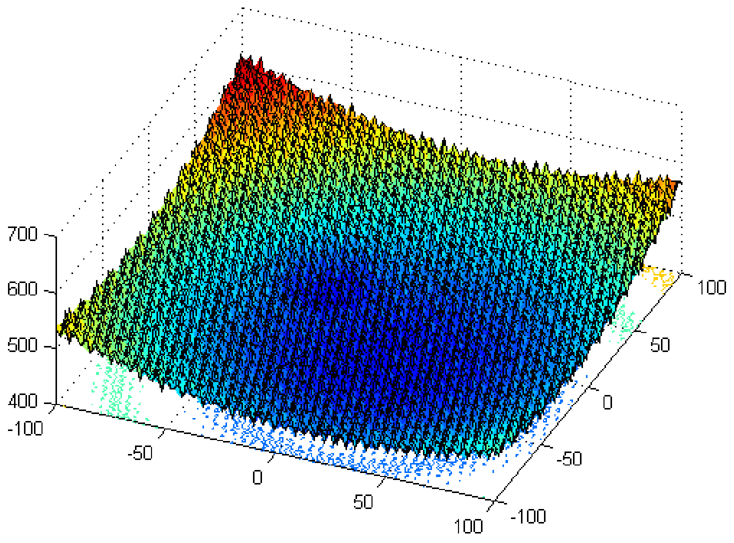

Figure 18(a). 3-*D* map for 2-*D* function

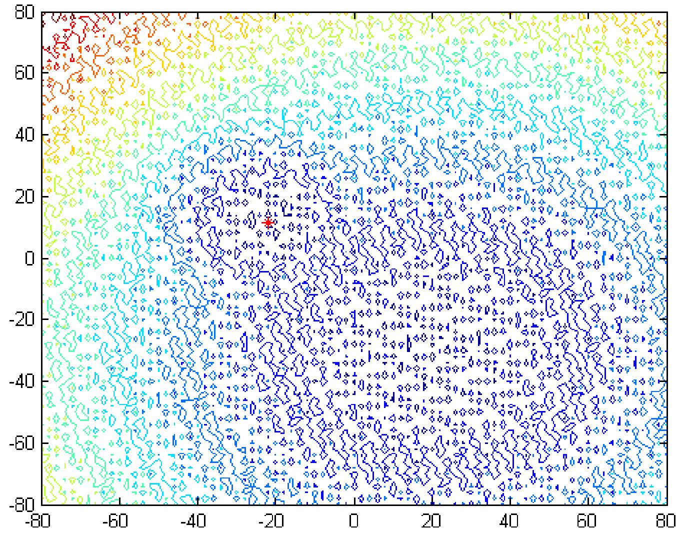

Figure 18(b).Contour map for 2-*D* function

Properties:

- Multi-modal
- Non-separable
- Asymmetrical
- Continuous everywhere yet differentiable nowhere

19) Rotated Expanded Griewank's plus Rosenbrock's Function

Basic Griewank's Function: $g_1(x) = \sum_{i=1}^{D} \frac{x_i^{\,2}}{4000} - \prod_{i=1}^{D} \cos(\frac{x_i}{\sqrt{i}}) + 1$

Basic Rosenbrock's Function: $g_2(\mathbf{x}) = \sum_{i=1}^{D-1} (100(x_i^2 - x_{i+1})^2 + (x_i - 1)^2)$

$$f_{19}(x) = g_1(g_2(z_1, z_2)) + g_1(g_2(z_2, z_3)) + ... + g_1(g_2(z_{D-1}, z_D)) + g_1(g_2(z_D, z_1)) + f_{19}{}^*(19)$$

$$z = \mathbf{M}_1(\frac{5(x-o)}{100}) + 1$$

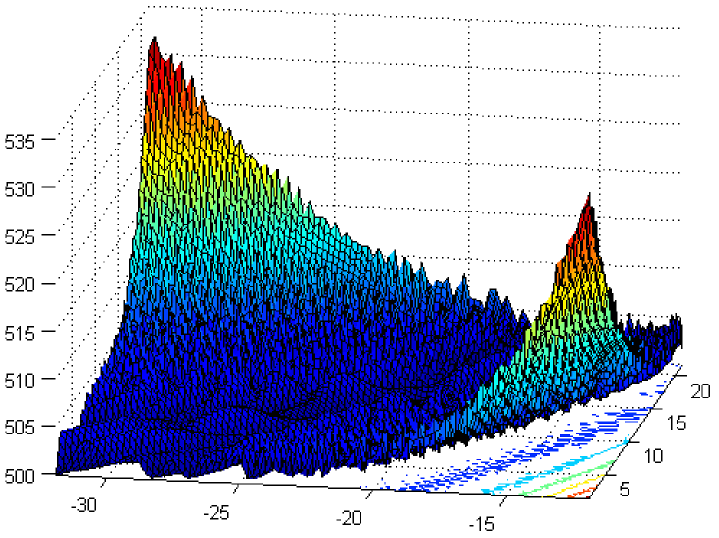

Figure 19(a). 3-*D* map for 2-*D* function

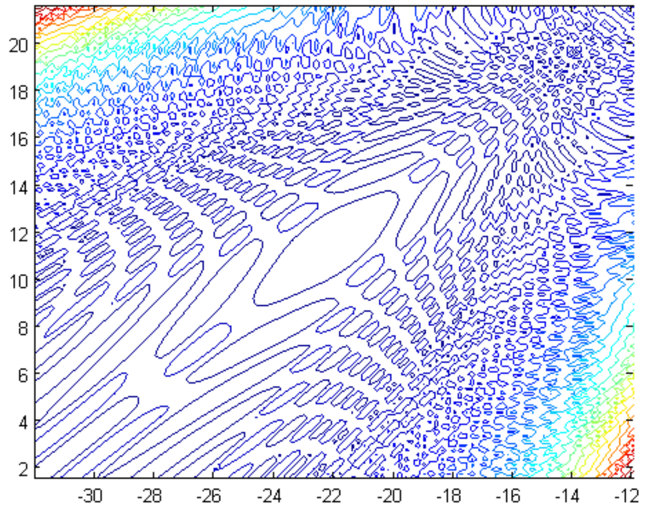

Figure 19(b).Contour map for 2-*D* function

Properties:

- Multi-modal
- Non-separable

20) Rotated Expanded Scaffer's F6 Function

Scaffer's F6 Function: $g(x,y)=0.5+\frac{(\sin^2(\sqrt{x^2+y^2})-0.5)}{(1+0.001(x^2+y^2))^2}$

$$f_{20}(\mathbf{x})=g(z_1,z_2)+g(z_2,z_3)+...+g(z_{D-1},z_D)+g(z_D,z_1)+f_{20}* \tag{20}$$

$$z=\mathbf{M}_2T_{asy}^{0.5}(\mathbf{M}_1(x-o))$$

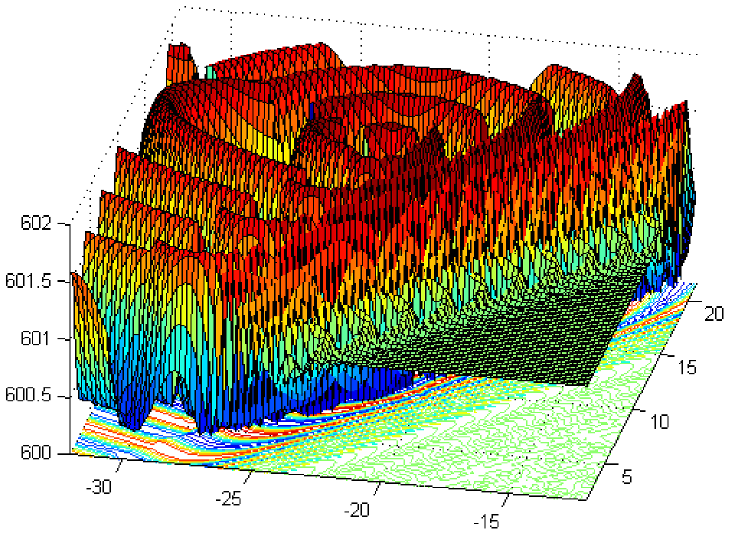

Figure 20(a). 3-*D* map for 2-*D* function

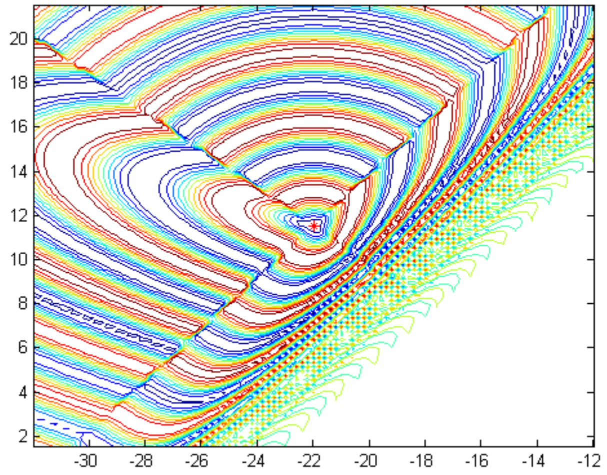

Figure 20(b).Contour map for 2-*D* function

Properties:

- Multi-modal
- Non-separable
- Asymmetrical

C. New Composition Functions

$$f(x)=\sum_{i=1}^{n}\{\omega_i*[\lambda_i g_i(x)+bias_i]\}+f* \quad (21)$$

f(*x*): new composition function

$g_i(x)$: i^{th} basic function used to construct the composition function

n: number of basic functions

o_i: new shifted optimum position for each $g_i(x)$,define the global and local optima's position

$bias_i$: define which optimum is global optimum

σ_i : used to control each $g_i(x)$'s coverage range, a small σ_i give a narrow range for that $g_i(x)$

λ_i : used to control each $g_i(x)$'s height

w_i : weight value for each $g_i(x)$, calculated as below:

$$w_i=\frac{1}{\sqrt{\sum_{j=1}^{D}(x_j-o_{ij})^2}}\exp\left(-\frac{\sum_{j=1}^{D}(x_j-o_{ij})^2}{2D\sigma_i^2}\right) \quad (19)$$

Then normalize the weight $\omega_i=w_i/\sum_{i=1}^{n}w_i$

So when $x = o_i$, $\omega_j = \begin{cases} 1 & j = i \\ 0 & j \neq i \end{cases}$ for $j = 1, 2, ..., n$, $f(x) = bias_i + f^*$

The local optimum which has the smallest bias values is global optimum.

Comparing with the previous composition functions in CEC'05, this new proposed composition function merges the properties of the sub-functions better and keeps continuous around the global/local optima.

Functions $f_i' = f_i - f_i^*$ are used as g_i. In this way, the function values of global optima of g_i are equal to 0 for all composition functions in this report.

21) Composition Function 1

$n = 5$, $\sigma = [10, 20, 30, 40, 50]$

$\lambda = [1, 1e\text{-}6, 1e\text{-}26, 1e\text{-}6, 0.1]$

$bias = [0, 100, 200, 300, 400]$

g_1: Rotated Rosenbrock's Function f_6'

g_2: Rotated Different Powers Function f_5'

g_3 Rotated Bent Cigar Function f_3'

g_4: Rotated Discus Function f_4'

g_5: Sphere Function f_1'

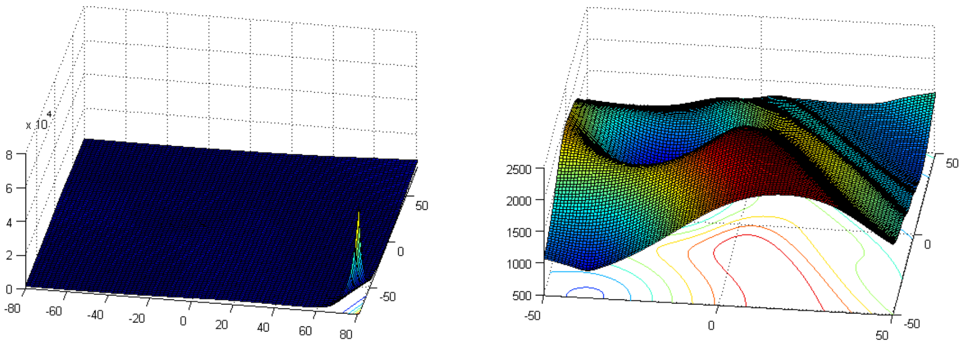

Figure 21(a). 3-*D* map for 2-*D* function

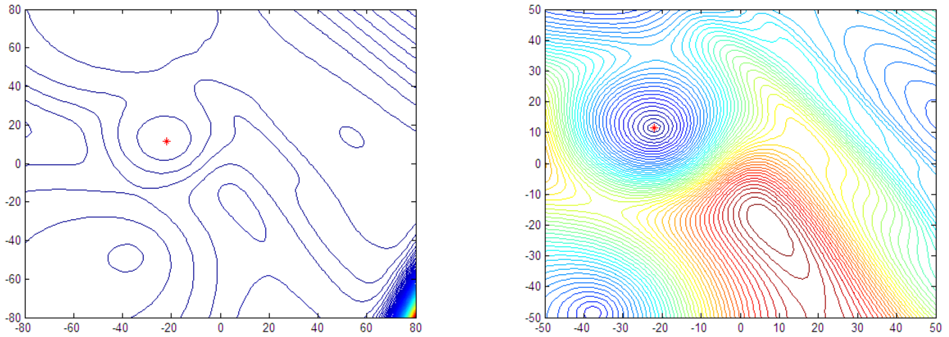

Figure 21(b).Contour map for 2-*D* function

Properties:

- Multi-modal
- Non-separable
- Asymmetrical
- Different properties around different local optima

22) Composition Function 2

$n = 3$

$\sigma = [20, 20, 20]$

$\lambda = [1, 1, 1]$

$bias = [0, 100, 200]$

$g_{1\text{-}3}$: Schwefel's Function f_{14}'

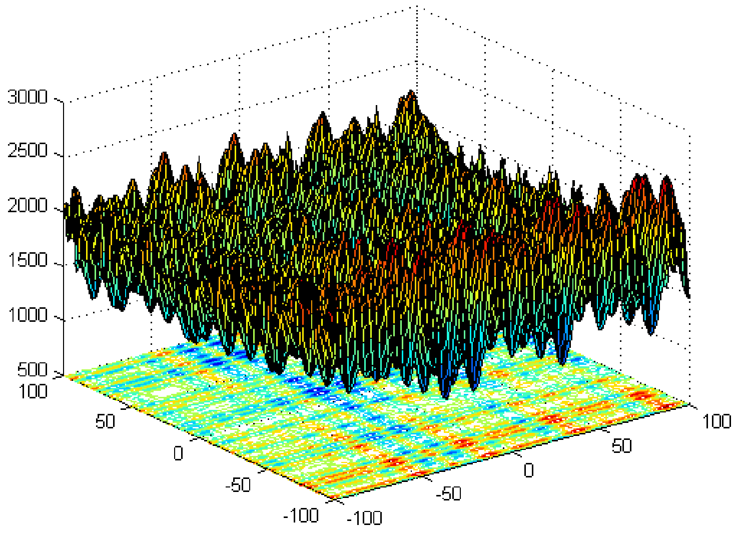

Figure 22(a). 3-*D* map for 2-*D* function

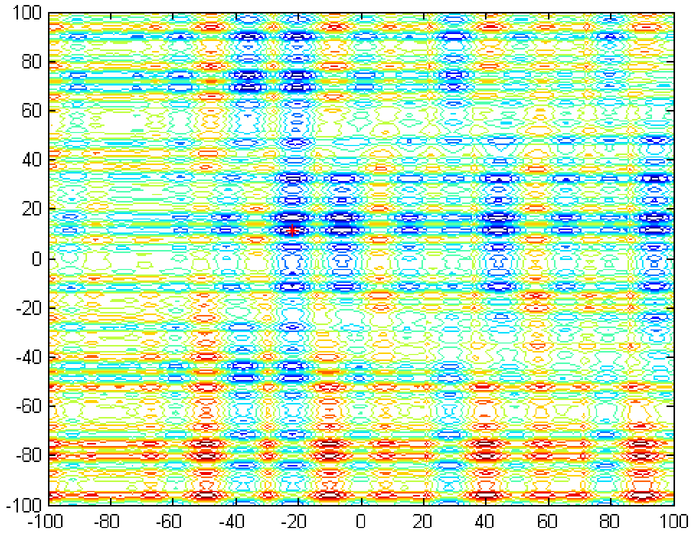

Figure 22(b).Contour map for 2-*D* function

Properties:

- ➢ Multi-modal
- ➢ Separable
- ➢ Asymmetrical
- ➢ Different properties around different local optima

23) Composition Function 3

$n = 3$

$\sigma = [20, 20, 20]$

$\lambda = [1, 1, 1]$

$bias = [0, 100, 200]$

$g_{1\text{-}3:}$ Rotated Schwefel's Function f_{15}'

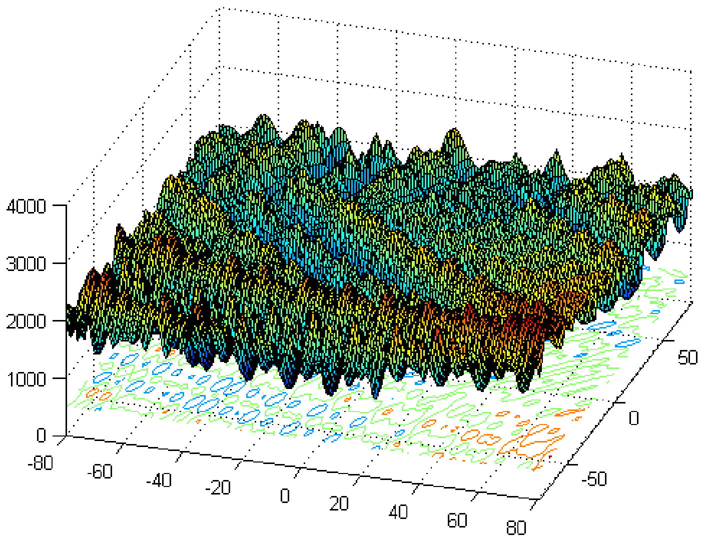

Figure 23(a). 3-*D* map for 2-*D* function

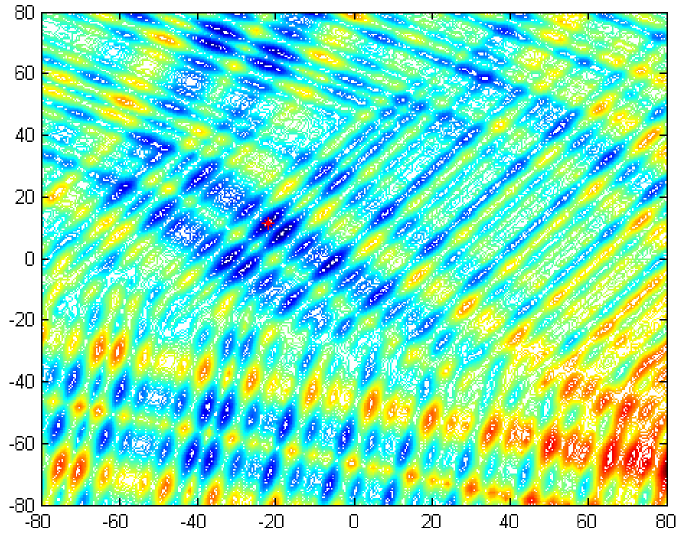

Figure 23(b).Contour map for 2-*D* function

Properties:

- Multi-modal
- Non-separable
- Asymmetrical
- Different properties around different local optima

24) Composition Function 4

$n = 3$

$\sigma = [20, 20, 20]$

$\lambda = [0.25, 1, 2.5]$

$bias = [0, 100, 200]$

g_1: Rotated Schwefel's Function f_{15}'

g_2: Rotated Rastrigin's Function f_{12}'

g_3: Rotated Weierstrass Function f_9'

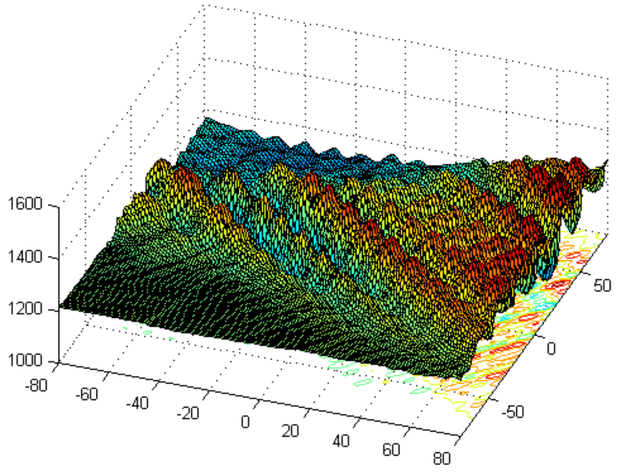

Figure 24(a). 3-*D* map for 2-*D* function

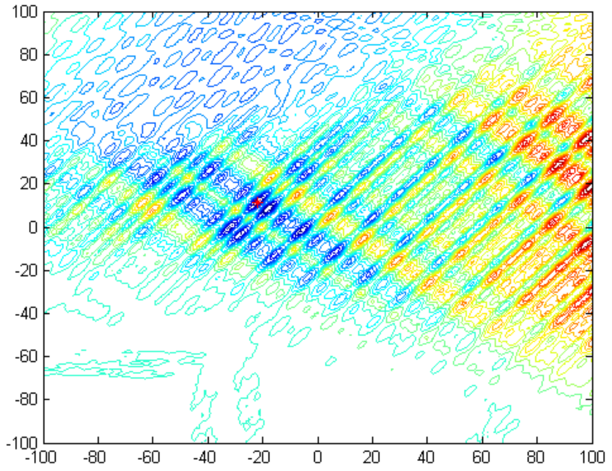

Figure 24(b).Contour map for 2-*D* function

Properties:

- Multi-modal
- Non-separable
- Asymmetrical
- Different properties around different local optima

25) Composition Function 5

All settings are same as Composition Function 4, except

σ = [10, 30, 50]

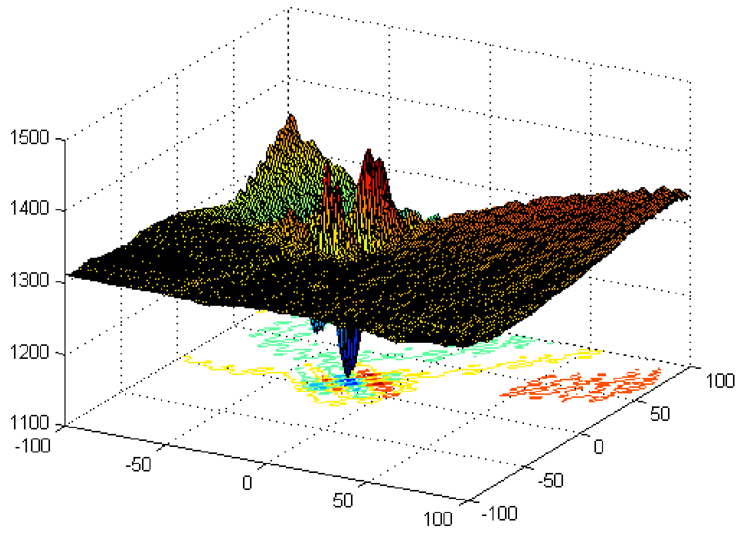

Figure 25(a). 3-*D* map for 2-*D* function

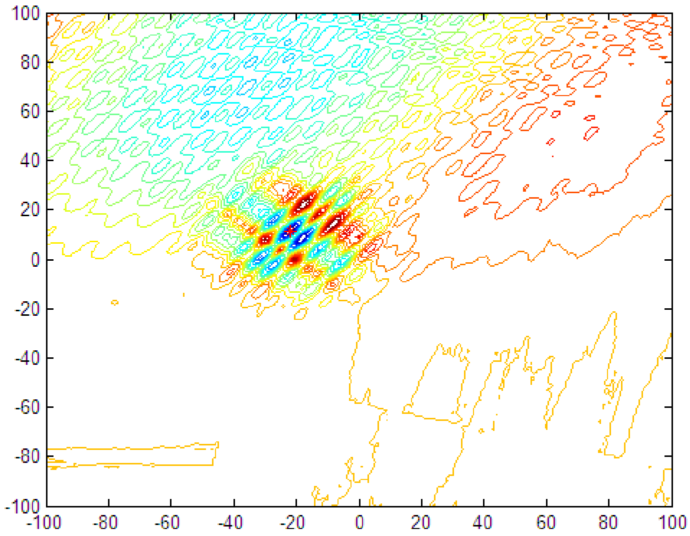

Figure 25(b).Contour map for 2-*D* function

Properties:

- Multi-modal

- Non-separable
- Asymmetrical
- Different properties around different local optima

26) Composition Function 6

$n = 5$

$\sigma = [10, 10, 10, 10, 10]$

$\lambda = [\ 0.25, 1, 1e\text{-}7, 2.5, 10]$

$bias = [0, 100, 200, 300, 400]$

$g_{1:}$ Rotated Schwefel's Function f_{15}’

$g_{2:}$ Rotated Rastrigin’s Function f_{12}’

$g_{3:}$ Rotated High Conditioned Elliptic Function f_2’

$g_{4:}$ Rotated Weierstrass Function f_9’

$g_{5:}$ Rotated Griewank’s Function f_{10}’

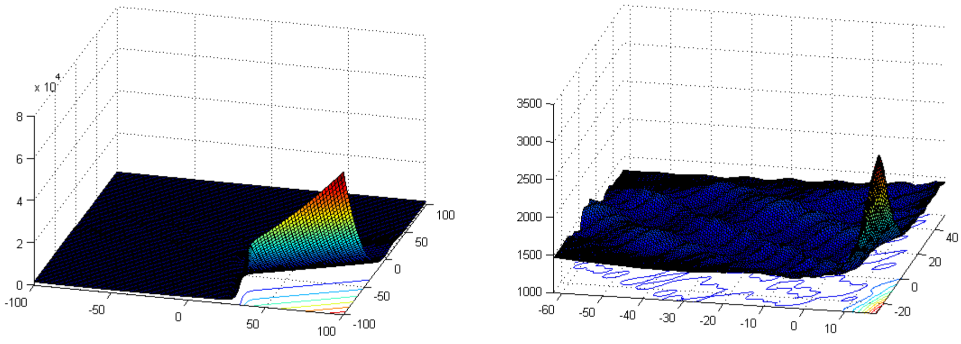

Figure 26(a). 3-*D* map for 2-*D* function

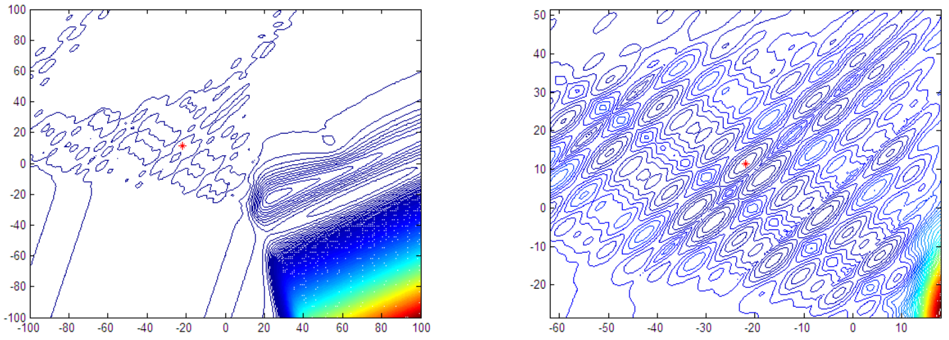

Figure 26(b).Contour map for 2-*D* function

Properties:

- Multi-modal
- Non-separable
- Asymmetrical
- Different properties around different local optima

27) Composition Function 7

$n = 5$

$\sigma = [10, 10, 10, 20, 20]$

$\lambda = [100, 10, 2.5, 25, 0.1]$

$bias = [0, 100, 200, 300, 400]$

g_1: Rotated Griewank's Function f_{10}'

g_2: Rotated Rastrigin's Function f_{12}'

g_3: Rotated Schwefel's Function f_{15}'

g_4: Rotated Weierstrass Function f_9'

g_5: Sphere Function f_1'

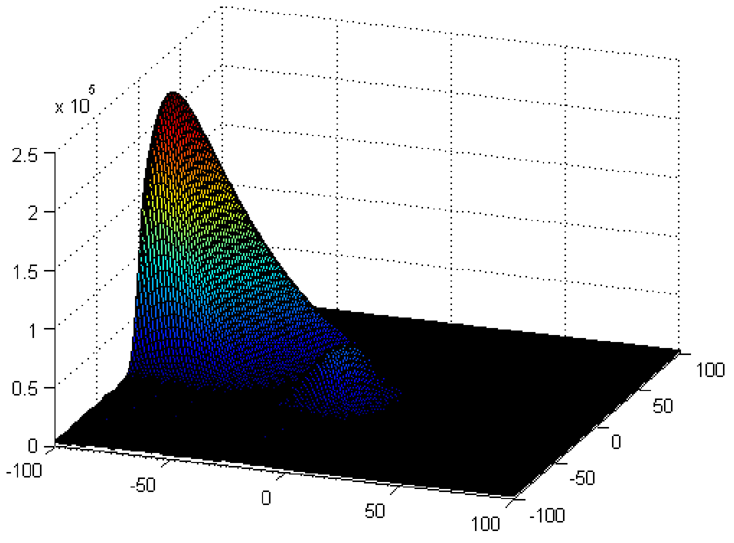

Figure 27(a). 3-*D* map for 2-*D* function

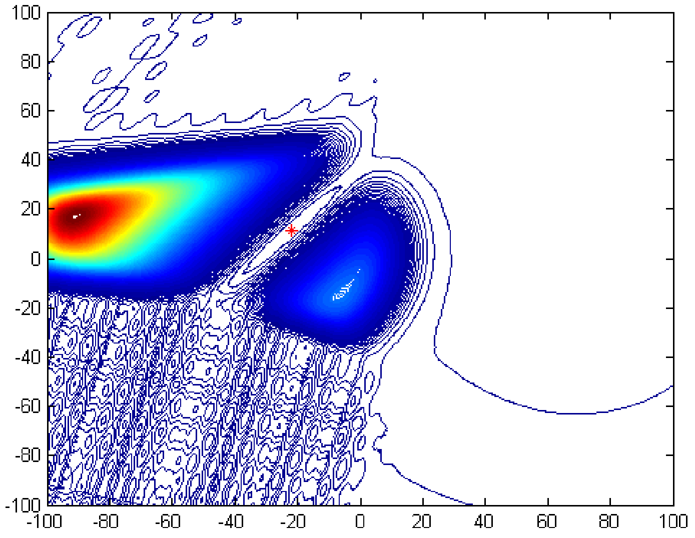

Figure 27(b).Contour map for 2-*D* function

Properties:

- Multi-modal

- Non-separable
- Asymmetrical
- Different properties around different local optima

28) Composition Function 8

$n = 5$

σ = [10, 20, 30, 40, 50]

λ = [2.5, 2.5e-3, 2.5, 5e-4,0.1]

bias = [0, 100, 200, 300, 400]

$g_{1:}$ Rotated Expanded Griewank's plus Rosenbrock's Function f_{19}'

$g_{2:}$ Rotated Schaffers F7 Function f_7'

$g_{3:}$ Rotated Schwefel's Function f_{15}'

$g_{4:}$ Rotated Expanded Scaffer's F6 Function f_{20}'

$g_{5:}$ Sphere Function f_1'

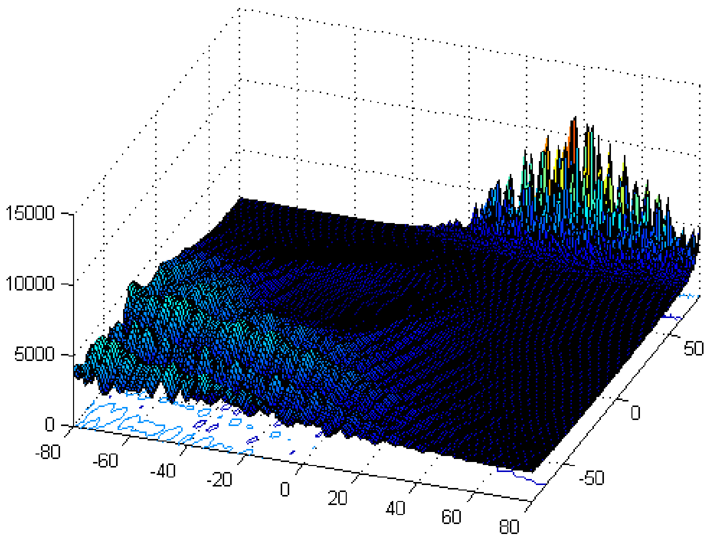

Figure 28(a). 3-*D* map for 2-*D* function

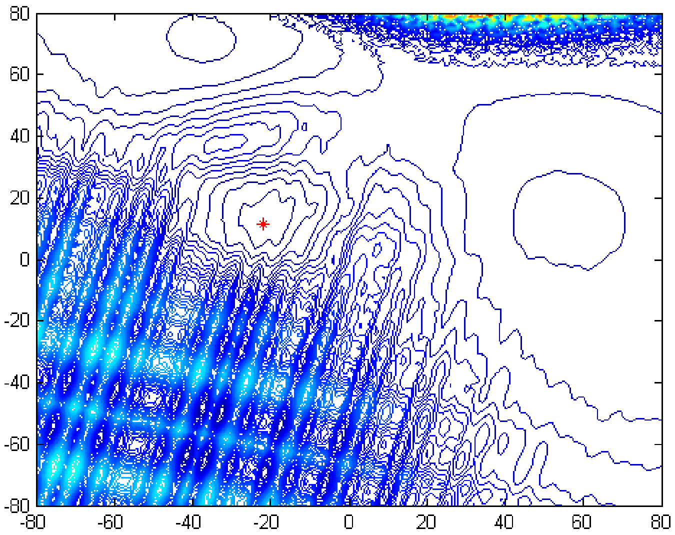

Figure 28(b).Contour map for 2-*D* function

Properties:

- Multi-modal
- Non-separable
- Asymmetrical
- Different properties around different local optima

2. Evaluation Criteria

2.1 Experimental Setting

Problems: 28 minimization problems

Dimensions: D=10, 30, 50 (Results only for 10D and 30D are acceptable for the initial submission; but 50D should be included in the final version of CEC 2013)

Runs / problem: 51 (**Do not run many 51 runs to pick the best 51 runs**)

MaxFES: 10000*D (Max_FES for 10D = 100000; for 30D = 300000; for 50D = 500000)

Search Range: $[-100,100]^D$

Initialization: Uniform random initialization within the search space. Random seed is based on time, Matlab users can use rand('state', sum(100*clock)).

Global Optimum: All problems have the global optimum within the given bounds and there is no need to perform search outside of the given bounds for these problems.

$f_i(x^*) = f_i(o) = f_i{}^*$

Termination: Terminate when reaching MaxFES or the error value is smaller than 10^{-8}.

2.1 Results to Record

1) **Record function error value ($f_i(x)$-$f_i(x^*)$) after (0.01, 0.1, 0.2, 0.3, 0.4, 0.5, 0.6, 0.7, 0.8, 0.9, 1.0)*MaxFES for each run.**

   In this case, 11 error values are recorded for each function for each run.

   Sort the error values achieved after MaxFES in 51 runs from the smallest (best) to the largest (worst) and present the **best, worst, mean, median** and **standard deviation** values of function error values for the 51 runs.

   Please Notice: Error values smaller than $\mathbf{10^{-8}}$ are taken as zero.

2) **Algorithm Complexity**

a) Run the test program below:

for i=1:1000000

x= 0.55 + (double) i;

x=*x* + *x*; *x*=*x*./2; *x=x*x; x=sqrt(x); x=log(x); x=exp(x); y=x/x;*

end

Computation time for the above=*T0*;

b) Evaluate the computation time just for **Function 14**. For 200000 evaluations of a certain dimension *D*, it gives T1;

c) The complete computation time for the algorithm with 200000 evaluations of the same *D* dimensional benchmark **function 14** is *T2*.

d) Execute step c 5 times and get 5 *T2* values. $\hat{T}2$ =Mean(*T2*)

The complexity of the algorithm is reflected by: $\hat{T}2$, *T1*, *T0*, and ($\hat{T}2$ -*T1*)/*T0*

The algorithm complexities are calculated on 10, 30 and 50 dimensions, to show the algorithm complexity's relationship with dimension. Also provide sufficient details on the computing system and the programming language used. In step c, we execute the complete algorithm 5 times to accommodate variations in execution time due adaptive nature of some algorithms.

Please Notice: Similar programming styles should be used for all T0, T1 and T2.

(For example, if m individuals are evaluated at the same time in the algorithm, the same style should be employed for calculating T1; if parallel calculation is employed for calculating T2, the same way should be used for calculating T0 and T1. In other word, the complexity calculation should be fair.)

3) Parameters

Participants are requested not to search for the best distinct set of parameters for each problem/dimension/etc. Please provide details on the following whenever applicable:

a) All parameters to be adjusted

b) Corresponding dynamic ranges

c) Guidelines on how to adjust the parameters

d) Estimated cost of parameter tuning in terms of number of FEs

e) Actual parameter values used.

Dimensionality and maximum available function evaluations can be used to design dynamic and adaptive algorithms.

4) Encoding

If the algorithm requires encoding, then the encoding scheme should be independent of the specific problems and governed by generic factors such as the search ranges, dimensionality of the problems, etc.

5) Results Format

The participants are required to send the final results as the following format to the organizers and the organizers will present an overall analysis and comparison based on these results.

Create one txt document with the name "AlgorithmName_FunctionNo._D.txt" for each test function and for each dimension.

For example, PSO results for test function 5 and D=30, the file name should be "PSO_5_30.txt".

Then save the results matrix (***the gray shadowing part***) as Table II in the file:

Table II. Information Matrix for *D* Dimensional Function X

***.txt	Run 1	Run 2	…	Run 51
Function error values when FES=0.01*MaxFES				
Function error values when FES=0.1*MaxFES				
Function error values when FES=0.2*MaxFES				
… …				
Function error values when FES=0.9*MaxFES				
Function error values when FES=MaxFES				

Thus **28*3** (10D, 30D and 50D) files should be zipped and sent to the organizers. Each file contains an **11*51** matrix.

Notice: All participants are allowed to improve their algorithms further after submitting the initial version of their papers to CEC2013. And they are required to

submit their results in the specified format to the organizers after submitting the **final** version of paper as soon as possible.

2.3 Results Template

Language: Matlab 2008a

Algorithm: Particle Swarm Optimizer (PSO)

Results

Notice:

Considering the length limit of the paper, only Error Values Achieved with MaxFES are need to be listed.

While the authors are required to send all results (28*2 files described in section 2.2) to the organizers for a better comparison among the algorithms.

Table III. Results for 10D

Func.	Best	Worst	Median	Mean	Std
1					
2					
3					
4					
5					
6					
7					
8					
9					
10					
11					
12					
13					
14					
15					
16					
17					
18					
19					
20					
21					
22					

**23**					
**24**					
**25**					
**26**					
**27**					
**28**					

Table IV. Results for 30D

…

Table V. Results for 50D

(mandatory in the final version, optionally in the 1st submission)…

Algorithm Complexity

Table XI. Computational Complexity

	$T0$	$T1$	$\hat{T}2$	$(\hat{T}2 - T1)/T0$
D=10				
D=30				
D=50				

Parameters

a) All parameters to be adjusted

b) Corresponding dynamic ranges

c) Guidelines on how to adjust the parameters

d) Estimated cost of parameter tuning in terms of number of FES

e) Actual parameter values used.

References

[1] P. N. Suganthan, N. Hansen, J. J. Liang, K. Deb, Y.-P. Chen, A. Auger & S. Tiwari, "Problem Definitions and Evaluation Criteria for the CEC 2005 Special Session on Real-Parameter Optimization," Technical Report, Nanyang Technological University, Singapore, May 2005 and KanGAL Report #2005005, IIT Kanpur, India, 2005.

[2] J. J. Liang, P. N. Suganthan & K. Deb, "Novel composition test functions for numerical global optimization," in Proc. of IEEE International Swarm Intelligence Symposium, pp. 68-75, 2005.

[3] Joaqu´ın Derrac, Salvador Garcia, Sheldon Hui, Francisco Herrera, Ponnuthurai N. Suganthan, "Statistical analysis of convergence performance throughout the search: A case study with SaDE-MMTS and Sa-EPSDE-MMTS," accepted by Symp. DE 2013, *IEEE SSCI 2013*, Singapore, April 2013.

[4] Nikolaus Hansen, Steffen Finck, Raymond Ros and Anne Auger,"Real-Parameter Black-Box Optimization Benchmarking 2010: Noiseless Functions Definitions" INRIA research report RR-6829, March 24, 2012.

View publication stats
