# Supplementary material for: An improved termite life cycle optimizer algorithm for global function optimization
Source: PeerJ Comput Sci. 2025 Feb 17;11:e2671. doi: 10.7717/peerj-cs.2671 (PMC11888856; doi:10.7717/peerj-cs.2671)
Supplement: Supplemental Information 4 [file peerj-cs-11-2671-s004.pdf]

The 100-Digit Challenge:

Problem Definitions and Evaluation Criteria for the *100-Digit Challenge* Special Session and Competition on Single Objective Numerical Optimization

K. V. Price[1], N. H. Awad[2], M. Z. Ali[3], P. N. Suganthan[2]

[1] Vacaville, California, USA

[2]School of EEE, Nanyang Technological University, Singapore

[3]School of Computer Information Systems, Jordan University of Science and Technology, Jordan

kvprice@pacbell.net, noor0029@ntu.edu.sg, mzali.pn@ntu.edu.sg, epnsugan@ntu.edu.sg

Technical Report

Nanyang Technological University, Singapore

November 2018

1. Introduction to the 100-Digit Challenge

1.1 Background

Research on single objective optimization algorithms often forms the foundation for more complex methods, such as niching algorithms and both multi-objective and constrained optimization algorithms. Traditionally, single objective benchmark problems are also the first test for new evolutionary and swarm algorithms. Additionally, single objective benchmark problems can be transformed into dynamic, niching composition, computationally expensive and many other classes of problems. It is with the goal of better understanding the behavior of evolutionary algorithms as single objective optimizers that we are introducing the 100-Digit Challenge.

The original SIAM 100-Digit Challenge was developed in 2002 by Oxford's Nick Trefethen in conjunction with the Society for Industrial and Applied Mathematics (SIAM) as a test for high-accuracy computing [1, 2]. Specifically, the challenge was to solve 10 hard problems to 10 digits of accuracy. One point was awarded for each correct digit, making the maximum score 100, hence the name. Contestants were allowed to apply any method to any problem and take as long as needed to solve it. Out of the 94 teams that entered, 20 scored 100 points and 5 others scored 99.

In a similar vein, we propose the 100-Digit Challenge. Like the SIAM version, there are 10 problems, which in our case are 10 functions to optimize, and the goal is to compute each function's minimum value to 10 digits of accuracy without being limited by time. In contrast to the SIAM version, however, the 100-Digit Challenge asks contestants to solve all ten problems with one algorithm, although limited control parameter "tuning" for each function will be permitted to restore some of the original contest's flexibility. Another difference is that the score for a given function is the average number of correct digits in the best 25 out of 50 trials (still a maximum of 10 points per function).

1.2 Contest Rationale

The current special session competitions on real parameter optimization strictly limit the maximum number of function evaluations, the reason being that time is a precious commodity in many real-world scenarios. For a similar reason, these contests often forbid algorithm tuning. The problem with this approach is that if allowed run-times are set too low, then rapid convergence will

be rewarded over final accuracy. The cautionary tale of the tortoise and the hare should remind us that sometimes slow and steady wins the race. There are many real-world situations in which time is less of an issue than getting the right answer. In practice, people will also tune an algorithm and run multiple trials if necessary. In this scenario, people need to know which algorithm will most likely succeed regardless of time and tuning effort. It is this aspect of numerical optimization that the 100-Digit Challenge addresses.

As mentioned above, recent contests measure the objective function value at "vertical" time-slices of the convergence plot (function value *vs*. function evaluations). The 100-Digit Challenge complements the current test bed by measuring function values at "horizontal" slices of the convergence plot. In earlier contests, the "horizontal slice" was a given "value-to-reach" (VTR) and the average number of function evaluations to attain the VTR was the primary statistic of performance. Previous contests have also relied on the success performance measure. One problem with this approach is that it depends on the notion of a success, which is undefined when an algorithm fails to attain the VTR. The 100-Digit Challenge, however, provides a more graduated way to measure "horizontal" performance (accuracy) because even "failures" can have some correct digits.

This competition requires participants to send their final results to the organizers in a technical report that adheres to the specified format. The organizers will present an overall analysis and comparison based on these results. We will also use statistical tests on convergence performance to compare algorithms that generate similar final solutions.

The C and Matlab codes for 100-Digit Challenge can be downloaded from the website given below:

http://www.ntu.edu.sg/home/EPNSugan/index_files/CEC2019

1.3 Some Definitions

- All test functions are minimization problems defined as follows:

$$\text{Min } f(\mathbf{x}),\ \mathbf{x} = [x_1, x_2 \ldots, x_D]^{\mathrm{T}}$$

- D: Dimensions.
- $\mathbf{o}_{i1} = [o_{i1}, o_{i2} \ldots, o_{iD}]^{\mathrm{T}}$: The shifted global optimum (defined in "shift_data_x.txt"), which is randomly distributed in $[-80, 80]^D$.

- All test functions are scalable. Functions 4–10 are valid for any $D > 1$, while functions 1–3 are valid for $D = 2n$, n^2 and $3n$, respectively, where $n = 1, 2...$ In addition, functions 4–10 are also shifted to **o** and rotated, whereas functions 1–3 are not.
- For convenience, the same search ranges are defined for functions 4–10. The search ranges for functions 1–3 are in Table I.
- $\mathbf{M}_i$: Rotation matrix. The rotation matrix for each subcomponent is generated from standard normally distributed entries by Gram-Schmidt ortho-normalization with a condition number c that is equal to 1 or 2. Different rotation matrices are assigned to functions 4–10 to guarantee that each function's parameters are fully linked. Functions 1–3 are fully parameter dependent and therefore not rotated.

1.4 Summary of the Basic Functions in the 100-Digit Challenge

Table 1. The 100-Digit Challenge Basic Test Functions

No.	Functions	$F_i^*=F_i(\mathbf{x}^*)$	D	Search Range
1	Storn's Chebyshev Polynomial Fitting Problem	1	9	[-8192, 8192]
2	Inverse Hilbert Matrix Problem	1	16	[-16384, 16384]
3	Lennard-Jones Minimum Energy Cluster	1	18	[-4,4]
4	Rastrigin's Function	1	10	[-100,100]
5	Griewangk's Function	1	10	[-100,100]
6	Weierstrass Function	1	10	[-100,100]
7	Modified Schwefel's Function	1	10	[-100,100]
8	Expanded Schaffer's F6 Function	1	10	[-100,100]
9	Happy Cat Function	1	10	[-100,100]
10	Ackley Function	1	10	[-100,100]

1.5 Definitions of the Basic Test Functions

1) Storn's Chebyshev Polynomial Fitting Problem

$$f_1(\mathbf{x}) = p_1 + p_2 + p_3,$$

$$p_1 = \begin{cases} (u-d)^2 & \text{if } u < d, \\ 0 & \text{otherwise;} \end{cases} \qquad u = \sum_{j=1}^{D} x_j\,(1.2)^{D-j}$$

$$p_2 = \begin{cases} (v - d)^2 & \text{if } v < d, \\ 0 & \text{otherwise;} \end{cases} \qquad v = \sum_{j=1}^{D} x_j\,(-1.2)^{D-j} \tag{1}$$

$$p_k = \begin{cases} (w_k - 1)^2 & \text{if } w_k > 1 \\ (w_k + 1)^2 & \text{if } w_k < 1 \\ 0 & \text{otherwise;} \end{cases} \qquad w_k = \sum_{j=1}^{D} x_j \left(\frac{2k}{m} - 1\right)^{D-j}$$

$$p_3 = \sum_{k=0}^{m} p_k, \quad k = 0, 1, \ldots, m, \qquad m = 32D.$$

$$d = 72.661 \text{ for } D = 9$$

2) Inverse Hilbert Matrix

$$f_2(\mathbf{x}) = \sum_{i=1}^{n} \sum_{k=1}^{n} \left| w_{i,k} \right|$$

$$\left(w_{i,k}\right) = \mathbf{W} = \mathbf{HZ} - \mathbf{I}, \qquad \mathbf{I} = \begin{bmatrix} 1 & 0 \ldots 0 \\ 0 & 1 \ldots 0 \\ \vdots & \ddots \\ 0 & 0 \ldots 1 \end{bmatrix} \tag{2}$$

$$\mathbf{H} = \left(h_{i,k}\right), \quad h_{i,k} = \frac{1}{i + k - 1}, \quad i, k = 1, 2, \ldots, n, \quad n = \sqrt{D}$$

$$\mathbf{Z} = \left(z_{i,k}\right), \quad z_{i,k} = x_{i + n(k-1)}$$

3) Lennard-Jones Minimum Energy Cluster

$$f_3(\mathbf{x}) = 12.7120622568 + \sum_{i=1}^{n-1} \sum_{j=i+1}^{n} \left(\frac{1}{d_{i,j}^2} - \frac{2}{d_{i,j}}\right), \tag{3}$$

$$d_{i,j} = \left(\sum_{k=0}^{2}\left(x_{3i+k-2} - x_{3j+k-2}\right)^2\right)^3, \; n = D/3$$

4) Rastrigin's Function

$$f_4(\mathbf{x}) = \sum_{i=1}^{D} \left(x_i^2 - 10\cos(2\pi x_i) + 10\right) \tag{4}$$

5) Griewank's Function

$$f_5(\mathbf{x}) = \sum_{i=1}^{D} \frac{x_i^2}{4000} - \prod_{i=1}^{D} \cos\left(\frac{x_i}{\sqrt{i}}\right) + 1 \tag{5}$$

6) Weierstrass Function

$$f_6(\mathbf{x}) = \sum_{i=1}^{D} \left(\sum_{k=0}^{k_{\max}} \left[a^k \cos\left(2\pi b^k (x_i + 0.5)\right) \right] \right) - D \sum_{k=0}^{k_{\max}} a^k \cos\left(\pi b^k\right) \tag{6}$$

$$a = 0.5,\ \ b = 3,\ \ k_{\max} = 20$$

7) Modified Schwefel's Function

$$f_7(\mathbf{x}) = 418.9829D - \sum_{i=1}^{D} g(z_i) \qquad z_i = x_i + 420.9687462275036$$

$$g(z_i) = \begin{cases} z_i \sin\left(|z_i|^{1/2}\right) & \text{if } |z_i| \leq 500 \\ \left(500 - \mathrm{mod}(z_i, 500)\right) \sin\left(\sqrt{|500 - \mathrm{mod}(z_i, 500)|}\right) - \dfrac{(z_i - 500)^2}{10000D} & \text{if } z_i > 500 \\ (\mathrm{mod}(|z_i|, 500) - 500) \sin\left(\sqrt{|\mathrm{mod}(z_i, 500) - 500|}\right) - \dfrac{(z_i + 500)^2}{10000D} & \text{if } z_i < -500 \end{cases} \tag{7}$$

8) Expanded Schaffer's F6 Function

$$g(x, y) = 0.5 + \frac{\sin^2\left(\sqrt{x^2 + y^2}\right) - 0.5}{\left(1 + 0.001(x^2 + y^2)\right)^2} \tag{8}$$

$$f_8(\mathbf{x}) = g(x_1, x_2) + g(x_2, x_3) \ldots + g(x_{D-1}, x_D) + g(x_D, x_1)$$

9) Happy Cat Function

$$f_9(\mathbf{x}) = \left| \sum_{i=1}^{D} x_i^2 - D \right|^{1/4} + \left(0.5 \sum_{i=1}^{D} x_i^2 + \sum_{i=1}^{D} x_i \right) \Big/ D + 0.5 \tag{9}$$

10) Ackley Function

$$f_{10}(\mathbf{x}) = -20\exp\left(0.2\sqrt{\frac{1}{D}\sum_{i=1}^{D} x_i^2}\right) - \exp\left(\frac{1}{D}\sum_{i=1}^{D}\cos(2\pi x_i)\right) + 20 + e \qquad (10)$$

1.6 Definitions of the 100-Digit Challenge Problems

1) Storn's Chebyshev Polynomial Fitting Problem

No maps. This function not defined for $D = 2$.

Properties:
- Multimodal with one global minimum
- Very highly conditioned
- Non-separable; fully parameter-dependent

$$F_1(\mathbf{x}) = f_1(\mathbf{x}) + F_1^* \qquad (11)$$

2) Inverse Hilbert Matrix Problem

No maps. This function not defined for $D = 2$.

Properties:
- Multi-modal with one global minimum
- Highly conditioned
- Non-separable; fully parameter-dependent

$$F_2(\mathbf{x}) = f_2(\mathbf{x}) + F_2^* \qquad (12)$$

3) Lennard-Jones Minimum Energy Cluster Problem

No maps. This function not defined for $D = 2$.

Properties:
- Multi-modal with one global minimum
- Non-separable; fully parameter-dependent

$$F_3(\mathbf{x}) = f_3(\mathbf{x}) = F_3^* \qquad (13)$$

4) Shifted and Rotated Rastrigin's Function

$$F_4(\mathbf{x}) = f_4(\mathbf{M}(\mathbf{x} - \mathbf{o}_4)) + F_4^* \tag{14}$$

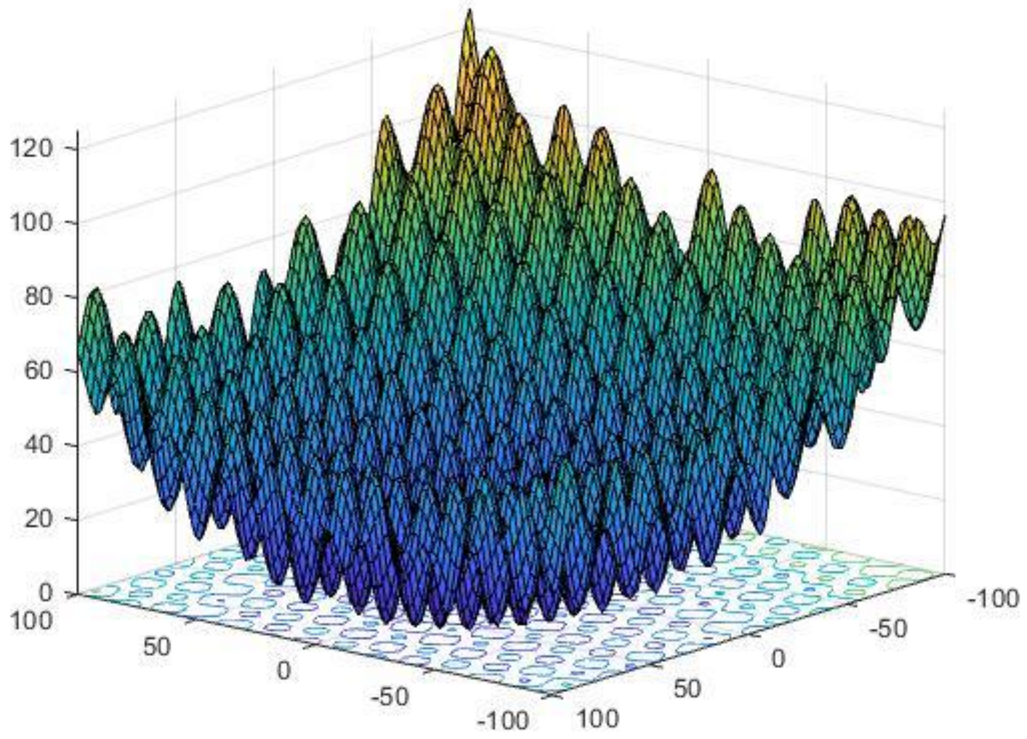

Figure 1(a). 3-*D* map for 2-*D* function

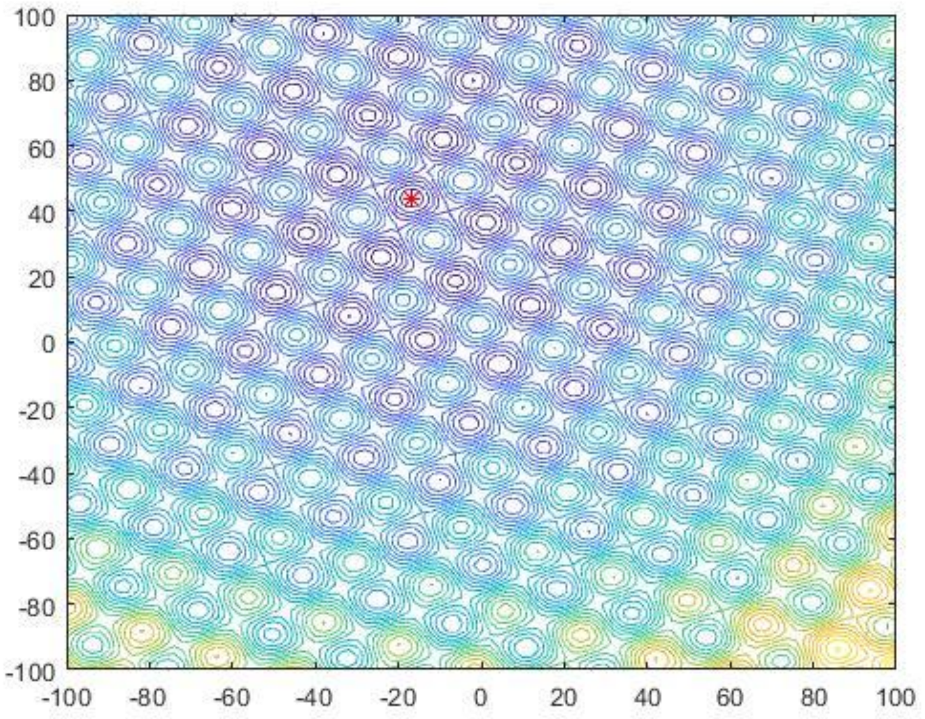

Figure 1(b).Contour map for 2-*D* function

Properties:

- Multi-modal
- Non-separable
- Local optima's number is huge and the penultimate optimum is far from the global optimum.

5) Shifted and Rotated Griewank's Function

$$F_5(\mathbf{x}) = f_5(\mathbf{M}(\mathbf{x} - \mathbf{o}_5)) + F_5^* \tag{15}$$

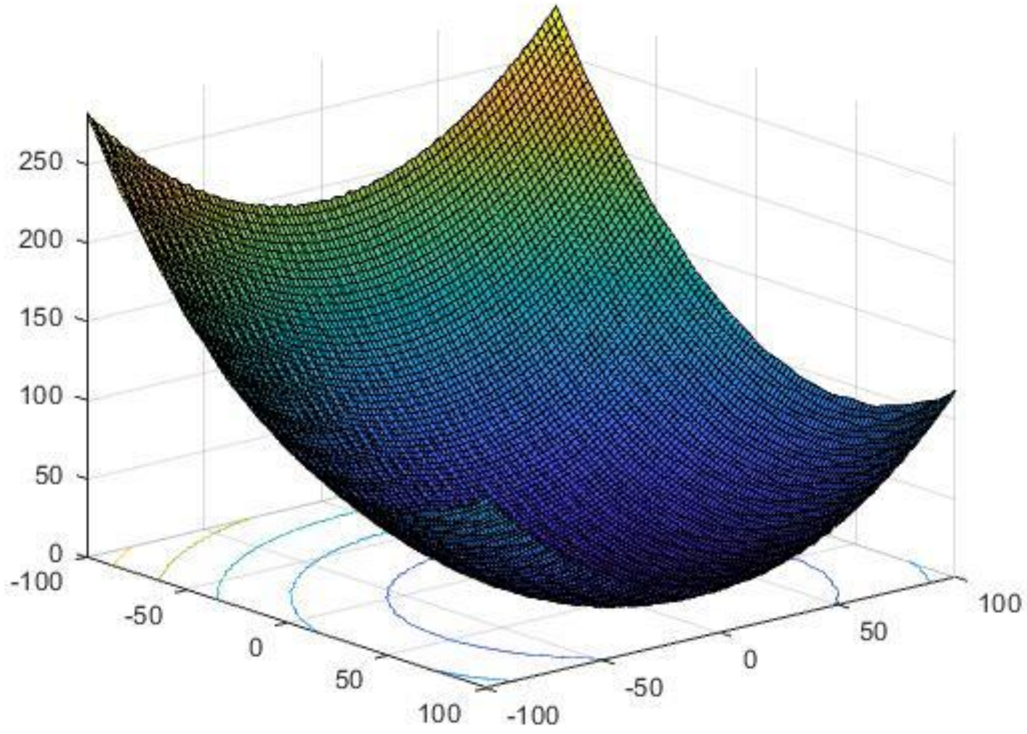

Figure 2(a). 3-*D* map for 2-*D* function

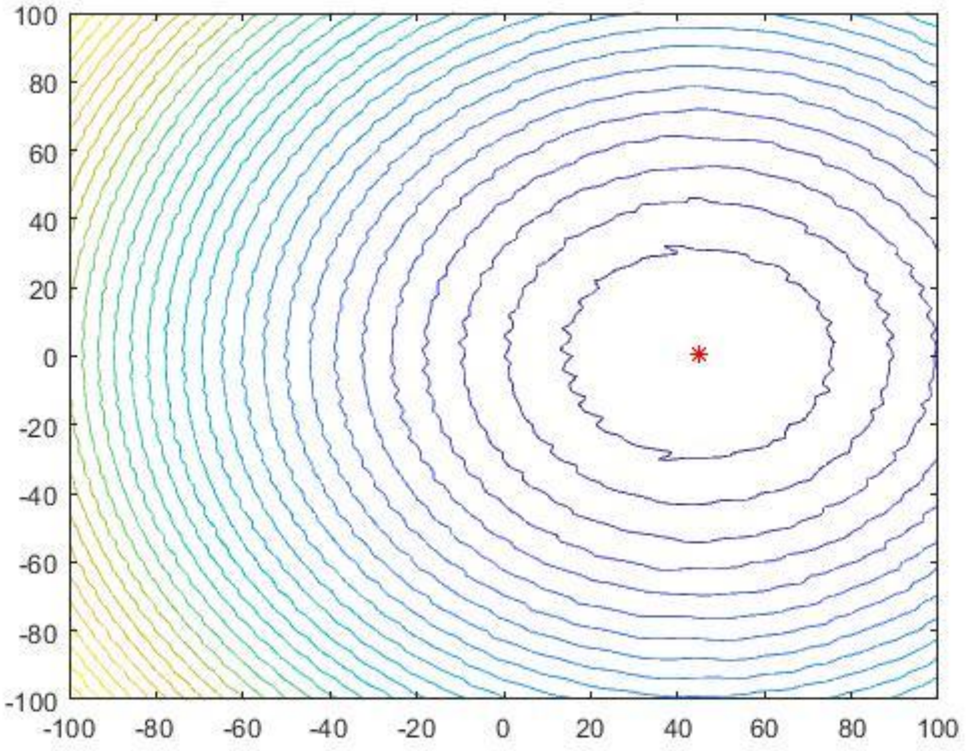

Figure 2(b).Contour map for 2-*D* function

Properties:

- Multi-modal
- Non-separable

6) Shifted and Rotated Weierstrass Function

$$F_6(\mathbf{x}) = f_6(\mathbf{M}(\mathbf{x} - \mathbf{o}_6) + F_6^* \qquad (16)$$

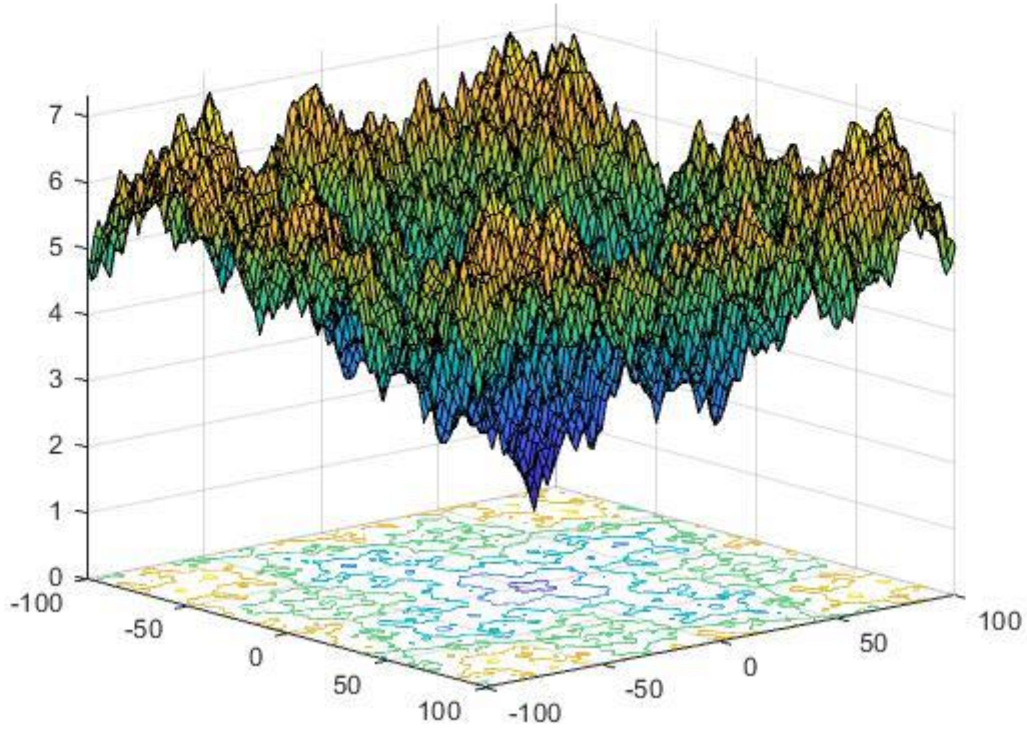

Figure 3(a). 3-*D* map for 2-*D* function

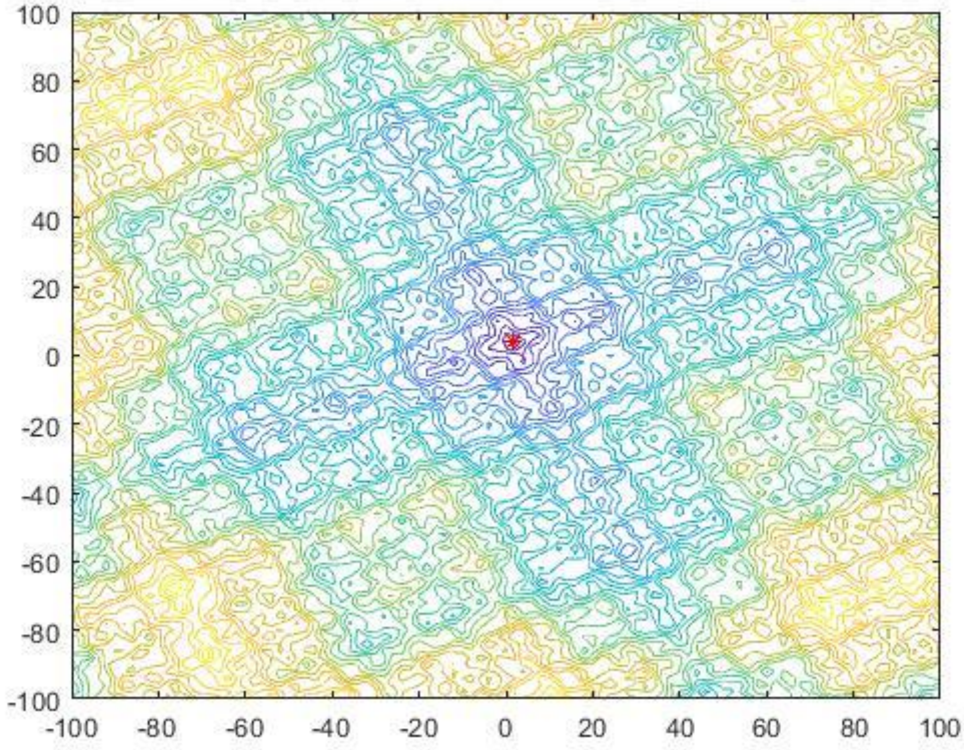

Figure 3(b).Contour map for 2-*D* function

Properties:

- Multi-modal
- Non-separable
- Local optima's number is huge

7) Shifted and Rotated Schwefel's Function

$$F_7(\mathbf{x}) = f_7(\mathbf{M}(\mathbf{x} - o_7) + F_7^* \quad (17)$$

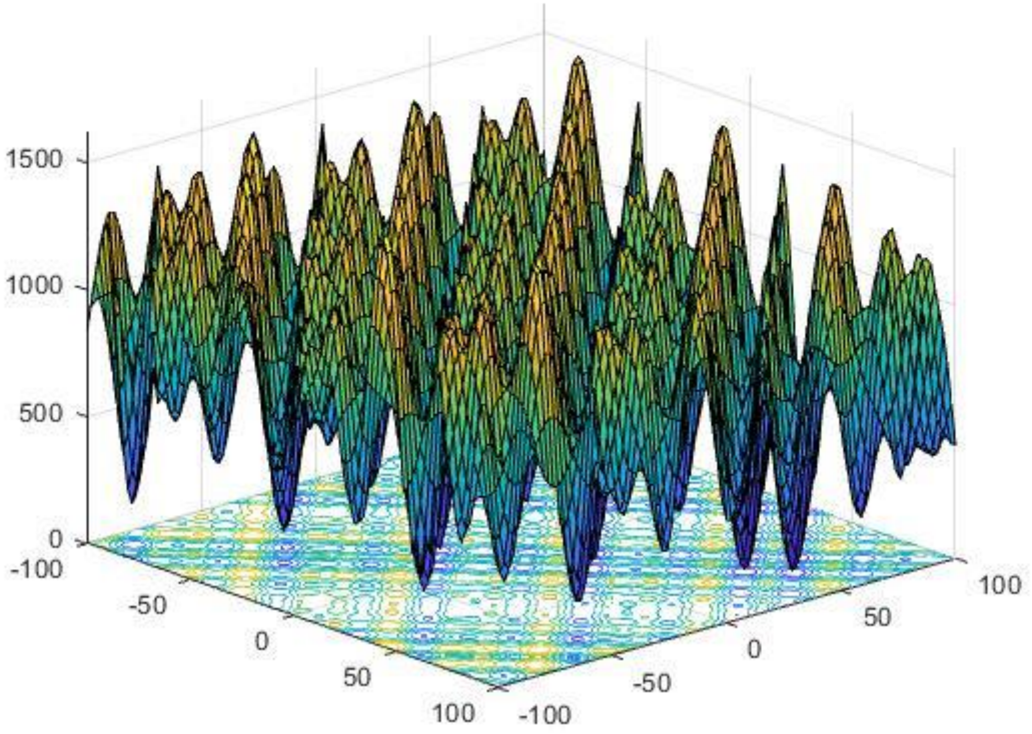

Figure 4(a). 3-*D* map for 2-*D* function

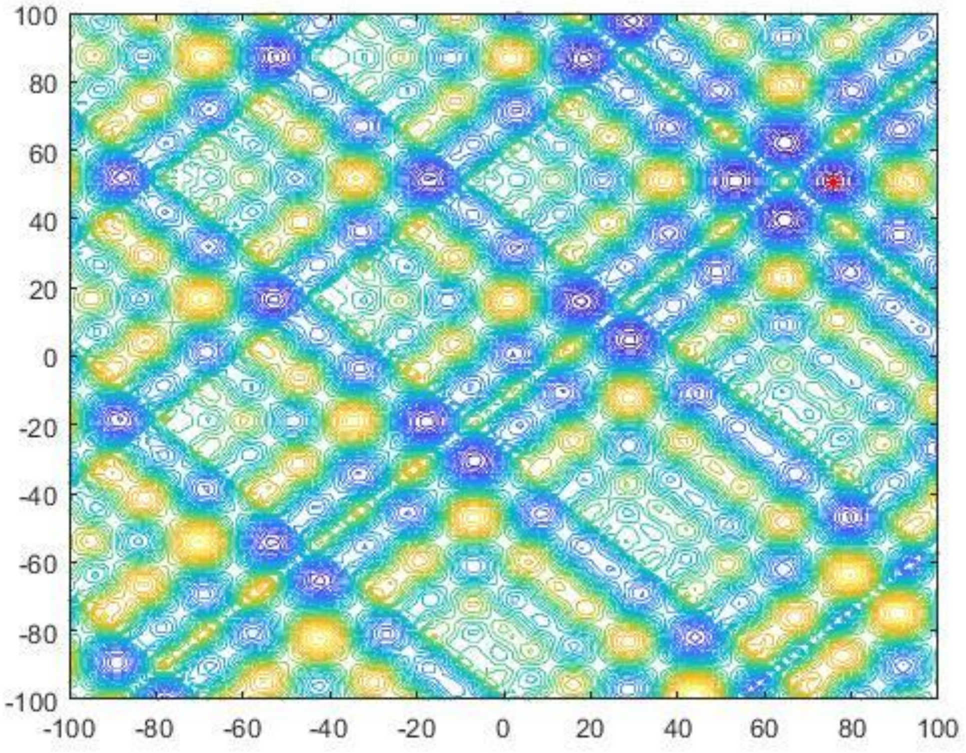

Figure 4(b).Contour map for 2-*D* function

Properties:

- Multi-modal
- Non-separable
- Local optima's number is huge

8) Shifted and Rotated Expanded Schaffer's F6 Function

$$F_8 = f_8(\mathbf{M}(0.005(\mathbf{x} - \mathbf{o}_8))) + F_8^* \quad (18)$$

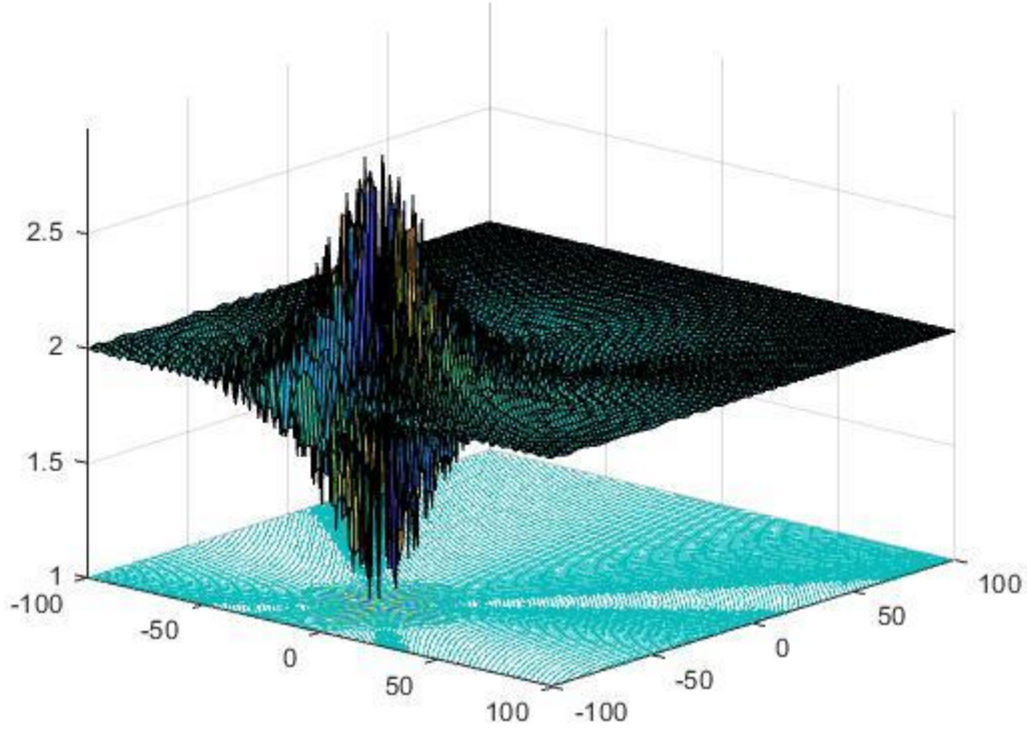

Figure 5(a). 3-*D* map for 2-*D* function

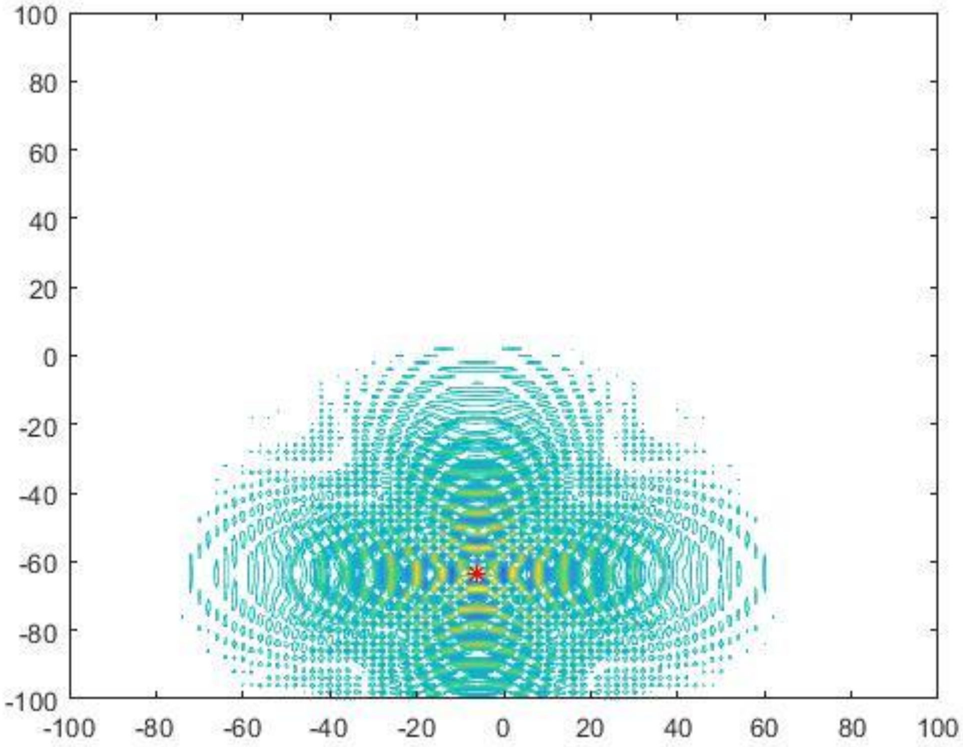

Figure 5(b).Contour map for 2-*D* function

Properties:

- Multi-modal
- Non-separable
- Local optima’s number is huge

9) Shifted and Rotated Happy Cat Function

$$F_9(\mathbf{x}) = f_9(\mathbf{M}(\mathbf{x} - \mathbf{o}_9)) + F_9^* \tag{19}$$

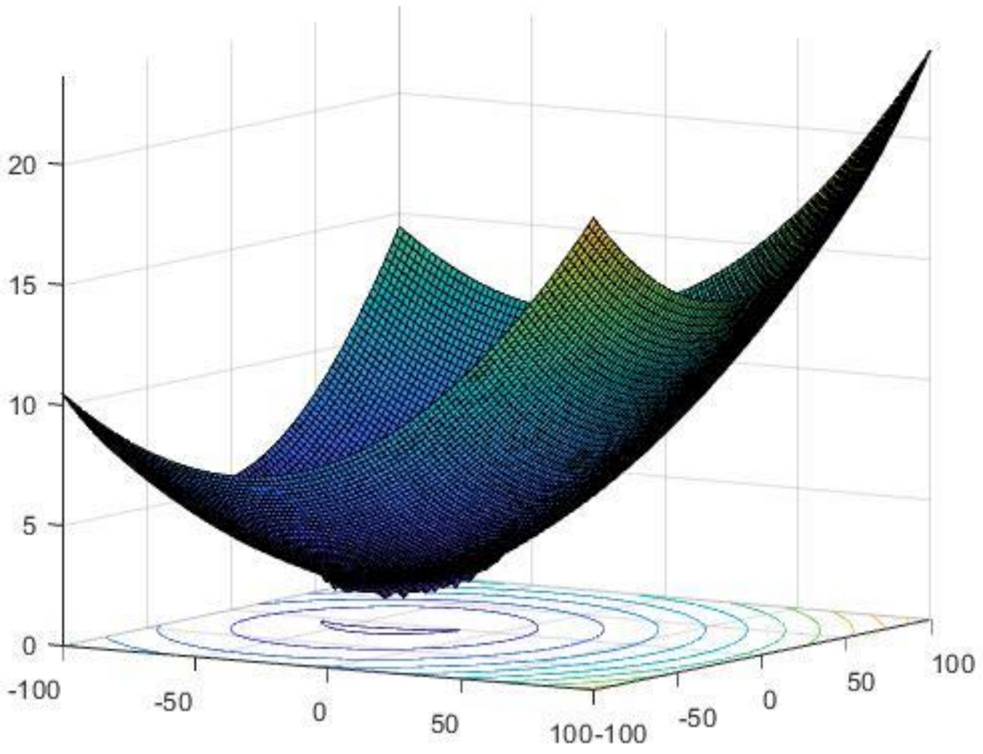

Figure 6(a). 3-*D* map for 2-*D* function

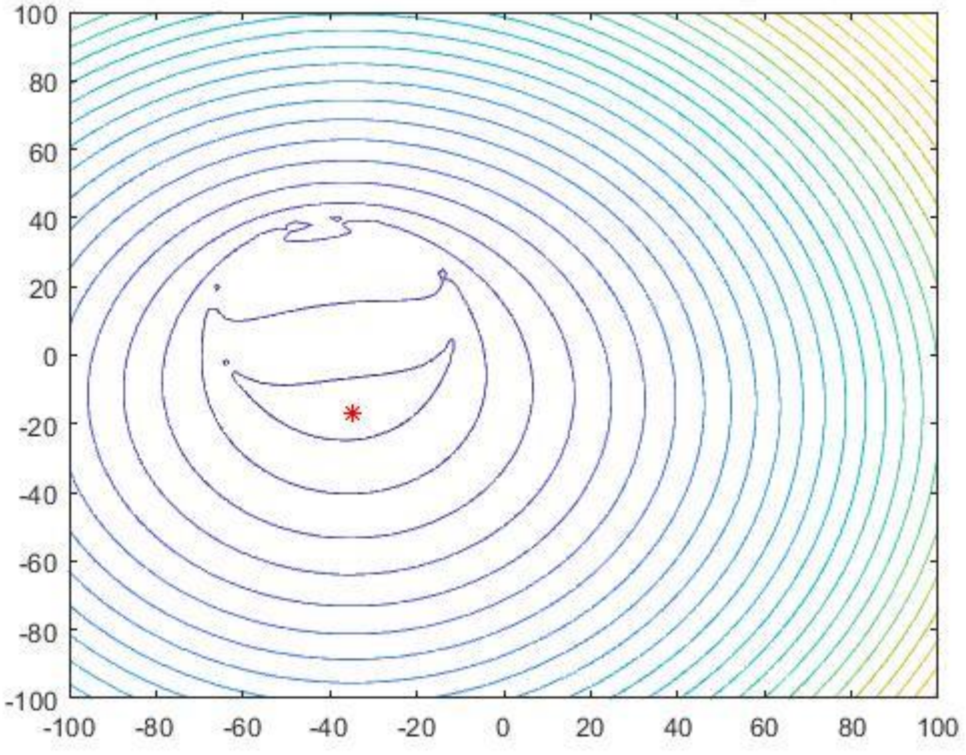

Figure 6(b).Contour map for 2-*D* function

Properties:

- Multi-modal
- Non-Separable

10) Shifted and Rotated Ackley Function

$$F_{10} = f_{10}(\mathbf{M}(\mathbf{x} - \mathbf{o}_{10})) + F_{10}^* \quad (20)$$

Properties:

- Multi-modal
- Non-Separable

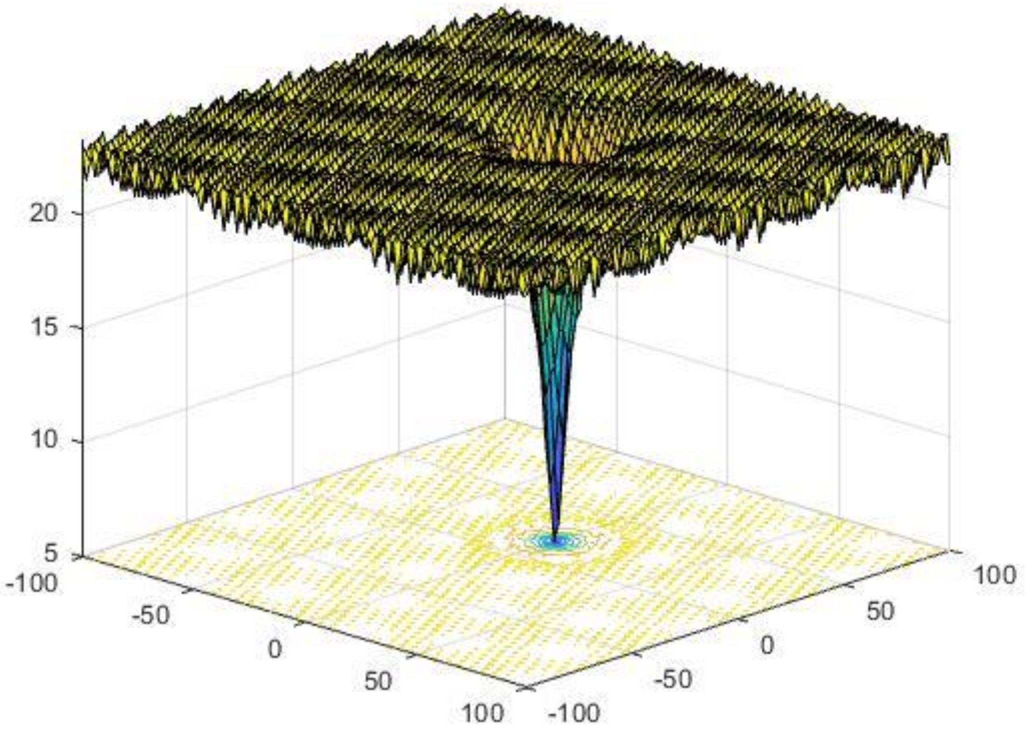

Figure 7(a). 3-*D* map for 2-*D* function

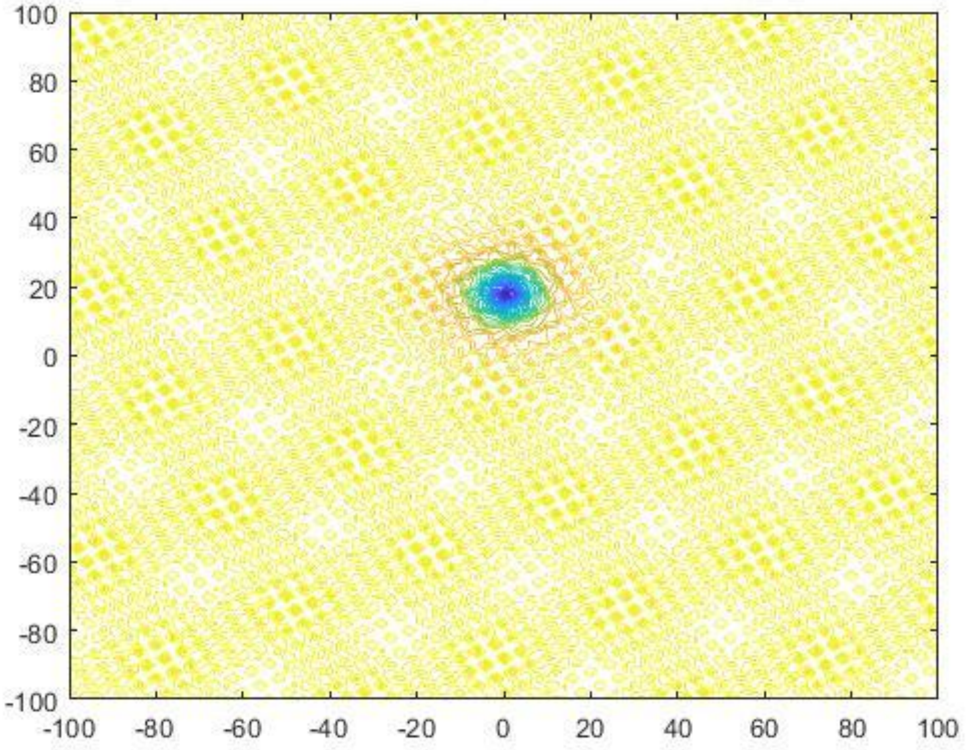

Figure 7(a). Contour map for 2-*D* function

2. Competition Rules

a) You may tune up to 2 parameter(s) and/or operator(s) independently for each problem. There is no limit to the number of parameters and operators that are fixed or adjusted in the identical manner for all 10 problem, but the two tunable parameter(s) and/or operator(s) must be the same for all 10 problems. Adaptive parameters do not count as tuned parameters provided that they are both initialized and adapted in the identical manner for all 10 problems.

b) For each function, run 50 consecutive trials, each with a different initial population (if applicable) and at one control parameter/operator setting of your choice, as specified in (a).

c) Count the total number of correct digits in the 25 trials that have the lowest function values.

d) The score for that function is the average number of correct digits in the best 25 trials, i.e. if 50% or more of the trials find all 10 digits, then the score for that function is a perfect 10.

e) The maximum score for the ten function total is 100, i.e. when the best 25 out of 50 trials for all 10 functions give the minimum to 10-digit accuracy.

f) The top three performing entries are required to release their codes online.

No tricks (since you already know the answers). You may not enlist special knowledge. Algorithm performance must not depend on the function number. These problems should be treated as black-box problems. You may not reference the explicit equations of the functions in your scheme.

Example: *How to Count Correct Digits*.

The minimum for all functions to ten digits of accuracy is 1.000000000. If your answer is:

- 2.000000000, then there are *no* correct digits even though the solution has 9 zeros to match the zeros in the optimal value.
- 1.924235666, then there is only one correct digit ("1").
- 1.003243567, then there are 3 correct digits ("1.00").

3. Evaluation Criteria

3.1 Experimental Setting

Problems: 10 minimization problems

Dimensions: As given in Table 1

Runs / problem: 50

MaxFES: (Maximum number of function evaluations). There is *no limit* on either time or the number of function evaluations.

Search Range: As given in Table 1

Initialization: Uniform random initialization within the search space. Random seed is based on time; Matlab users can use rand ('state', sum(100*clock)).

Global Optimum: All problems have the global optimum within the given bounds and there is no need to perform a search outside of the given bounds for these problems:

$F_i(\mathbf{x}^*) = F_i(\mathbf{o}_i) = F_i^* = 1.000000000$

Termination: Terminate a run when reaching the 10-digit level of accuracy or when you are satisfied that no further improvement is possible.

3.2 Presenting Results

Below is the template for presenting results. It consists of four parts:

- **A text file:** (see Table 2 below) A results record that records the number of function evaluations that each trial took to reach the 1, 2…, 10-digit level of accuracy. In the last row, record the number of function evaluations when the run was terminated.
- **A table:** (see Table 3 below) that lists for each function the number of trials in a run of 50 that found *n* correct digits, where *n* = 1, 2…, 10. In the final column, enter the average number of correct digits in the best 25 runs, i.e. the score for that function. Enter the total score (the sum of the scores for all 10 functions) in the bottommost right-hand cell.
- **A table:** (see Table 4 below) that records the values of at most two parameters that were tuned. For each function, report the values that that gave the results in Tables 2 and 3.
- **A paper** that contains both Tables 3 and 4, and
    - Describes your algorithm
    - Lists the dynamic ranges for tuned parameters
    - Provides guidelines on how to adjust the tuned parameters
    - Reports the default values of other important parameters of your algorithm that were held constant

- Describes your termination criterion when a trial does not reach the 10-digit level of accuracy

3.3 Text File Template

Language: Matlab 2017a

Algorithm: Particle Swarm Optimizer (PSO)

Record the number of function evaluations (FEs) after (1, 2, 3, 4, 5, 6, 7, 8, 9, 10) digit(s) of accuracy for each of the best 25 runs. If the given level of accuracy is not reached, leave the corresponding cell empty.

Create one txt document with the name “AlgorithmName_FunctionNo._D.txt” for each test function for the given dimension. For example, PSO results for test function 5 and *D* = 10, the file name should be “PSO_5_10.txt”. Save the results in a matrix (***the gray shadowing part***) as Table 2 in the file:

Table 2. Information Matrix for *D* Dimensional Function X

***.txt	Run 1	Run 2	…	Run 25
Function evaluations to reach 1-digit accuracy				
Function evaluations to reach 2-digit accuracy				
Function evaluations to reach 3-digit accuracy				
Function evaluations to reach 4-digit accuracy				
… …				
Function evaluations to reach 9-digit accuracy				
Function evaluations to reach 10-digit accuracy				
Function evaluations at termination				

3.4 Template for results to be tabulated in your paper

Table 3. Fifty runs for each function sorted by the number of correct digits

Function	Number of correct digits											Score
	0	1	2	3	4	5	6	7	8	9	10	
1												
2												

3												
4												
5												
6												
7												
8												
9												
10												
Total:												

Table 4. Tuned Parameter Values

Function	(Parameter 1 name)*	(Parameter 2 name)*
1		
2		
3		
4		
5		
6		
7		
8		
9		
10		

* Replace with your tuned parameters’ names.

Notice: All participants are allowed to improve their algorithms further after submitting the initial version of their papers. And they are required to submit their results in the introduced format to the organizers after submitting the final version of paper as soon as possible. The participants are required to send the final results as the specified format to the organizers who will present an overall analysis and comparison based on these results.

References

[1] N. Trefethen, A Hundred-dollar, Hundred-digit Challenge, SIAM News. 35 (1): 65, 2002.

[2] F. Bornemann, D. Laurie, S. Wagon, J. Waldvogel, The SIAM 100-digit challenge: A study in high-accuracy numerical computing. Philadelphia, PA: Society for Industrial and Applied Mathematics (SIAM), 2004.
