# Supplementary material for: An improved termite life cycle optimizer algorithm for global function optimization
Source: PeerJ Comput Sci. 2025 Feb 17;11:e2671. doi: 10.7717/peerj-cs.2671 (PMC11888856; doi:10.7717/peerj-cs.2671)
Supplement: Supplemental Information 5 [file peerj-cs-11-2671-s005.pdf]

ResearchGate

See discussions, stats, and author profiles for this publication at: https://www.researchgate.net/publication/338711283

Problem Definitions and Evaluation Criteria for the CEC 2020 Special Session on Multimodal Multiobjective Optimization

Technical Report · December 2019

DOI: 10.13140/RG.2.2.31746.02247

CITATIONS
52

READS
7,044

5 authors, including:

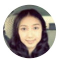

Jing Liang
Zhengzhou University
120 PUBLICATIONS **17,651** CITATIONS
SEE PROFILE

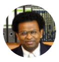

Ponnuthurai N. Suganthan
Qatar University
653 PUBLICATIONS **67,359** CITATIONS
SEE PROFILE

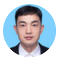

Cai Tong Yue
Zhengzhou University
107 PUBLICATIONS **3,478** CITATIONS
SEE PROFILE

All content following this page was uploaded by Cai Tong Yue on 21 January 2020.

The user has requested enhancement of the downloaded file.

Problem Definitions and Evaluation Criteria for the CEC 2020 Special Session on Multimodal Multiobjective Optimization

J. J. Liang[1], P. N. Suganthan[2], B. Y. Qu[3], D. W. Gong[4] and C. T. Yue[1]

[1] School of Electrical Engineering, Zhengzhou University, Zhengzhou, China
[2] School of EEE, Nanyang Technological University, Singapore
[3] School of Electric and Information Engineering, Zhongyuan University of Technology, Zhengzhou, China
[4] School of Information and Control Engineering, China University of Mining and Technology, Xuzhou, China
liangjing@zzu.edu.cn, epnsugan@ntu.edu.sg, qby1984@hotmail.com, dwgong@vip.163.com, zzuyuecaitong@163.com

Technical Report 201912, Computational Intelligence Laboratory, Zhengzhou University, Zhengzhou China
And
Technical Report, Nanyang Technological University, Singapore

December 2019

In multiobjective optimization problems, there may exist two or more global or local Pareto optimal sets (PSs) and some of them may correspond to the same Pareto Front (PF). These problems are defined as multimodal multiobjective optimization problems (MMOPs) [1, 2]. Arguably, finding one of these multiple PSs may be sufficient to obtain an acceptable solution for some problems. However, failing to identify more than one of the PSs may prevent the decision maker from considering solution options that could bring about improved performance. Recently, many researchers [3-10] proposed different multimodal multiobjective optimization (MMO) algorithms, so there is definitely a need of evaluating these algorithms in a more systematic manner on an open and fair competition platform.

In the MMO test suite of CEC'2020, a set of MMO test problems with different characters are designed, such as problems with different shape of PSs and PFs, coexistence of local and global PSs, scalable number of PSs, decision variables and objectives. In addition, a fair and appropriate evaluation criterion and reference data are given to assess the performance of different MMO algorithms.

The Matlab codes for the MMO test suite of CEC'2020 can be downloaded from the website given below:

https://github.com/P-N-Suganthan

1 Introduction to the CEC'2020 MMO test problems

1.1 Some Definitions

Given a multiobjective optimization problem Min $\vec{f}(\vec{x}) = \left[f_1(\vec{x}), f_2(\vec{x}), \ldots, f_m(\vec{x}) \right]$, a feasible solution $\vec{x_1}$ is said to dominate [1] the other feasible $\vec{x_2}$ if both of the two conditions are met:

1) The solution $\vec{x_1}$ is no worse than $\vec{x_2}$ for all objectives, i.e. $f_i(\vec{x_1}) \le f_i(\vec{x_2})$ for $i = 1, \ldots, m$;

2) The solution $\vec{x_1}$ is strictly better than $\vec{x_2}$ for at least one objective, i.e. $f_i(\vec{x_1}) < f_i(\vec{x_2})$ for $i \in [1, m]$.

If a solution is not dominated by any other solutions, it is called a *nondominated* solution. The *nondominated* solution set is called Pareto optimal set (PS). The set of vectors in the objective space that corresponds to the PS is called Pareto front (PF).

The definitions of Local PS, PF and Global PS, PF [1, 11]are as follows:

Local Pareto optimal set (Local PS): For arbitrary solution $\vec{x}$ in a solution set P_L, if there is no neighborhood solution $\vec{y}$ satisfying $\left\|\vec{y} - \vec{x}\right\|_\infty \le \sigma$ (σ is a small positive value), dominating any solution in the set P_L, then P_L is called Local Pareto optimal set;

Global Pareto optimal set (Global PS): For arbitrary solution in a solution set P_G, if there is no solution dominating any solution in the set P_G, then P_G is called Global Pareto optimal set.

Local Pareto Front (Local PF): The set of all the vectors in the objective space that corresponds to the Local PS is defined as Local Pareto Front.

Global Pareto Front (Global PF): The set of all the vectors in the objective space that corresponds to the Global PS is defined as Global Pareto Front.

Fig. 1 shows a bi-objective minimization problem with two Global PSs and one Local PS. Solid lines with stars are global PS/PF, while dashed lines with circles dots represent local PS/PF. Note that a certain multimodal multiobjective problem may have several Local PSs and Global PSs.

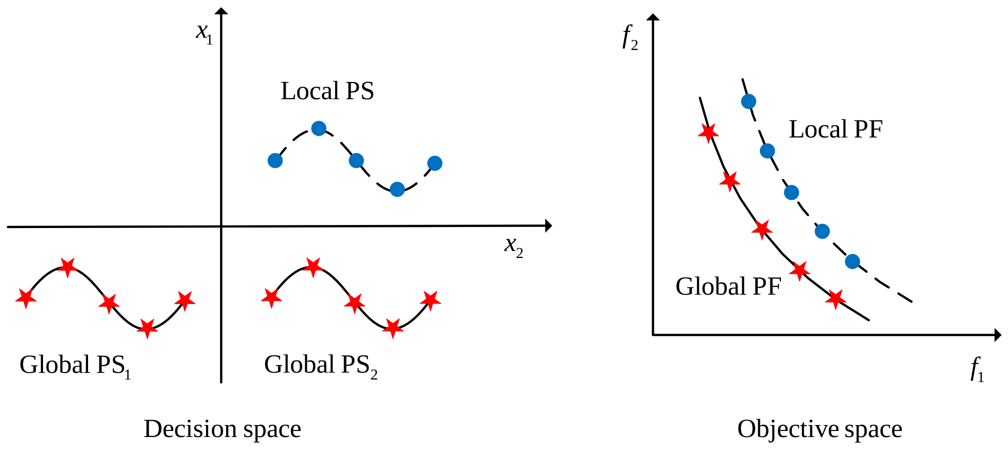

Fig. 1. Illustration of Local PS, Global PS, Local PF, and Global PF.

The method to judge whether a given multiobjective optimization problem is MMO problem or not is given in this report. For a multiobjective optimization problem, if it meets one of the following conditions, it is a MMO problem:

1) It has at least one local Pareto optimal solution;

2) It has at least two global Pareto optimal solutions corresponding to the same point on the PF.

The solution which is not dominated by any neighborhood solution is called local Pareto optimal solution. The solution which is not dominated by any solutions in the feasible space is called global Pareto optimal solution.

1.2 Summary of the CEC'2020 MMO test problems

The characters of the MMO test functions are shown in Table I. In the last column of Table I, *N*_ops represents the number of PS to be obtained. *N*_ops = *N*_global + *N*_local where *N*_global represents the number of global PS need to be obtained and *N*_local represent the number of local PSs need to be obtained. Only the shading problems' local PS need to be obtained. The equations of MMF10 and MMF10_*l* are the same, but their reference data are different. The reference data of MMF10 only include global PS and PF while those of MMF10_*l* include both local and global PS and PF. MMF11_*l*, MMF12_*l*, MMF13_*l*, MMF15_*l* and MMF15_a_*l* are of the same situation.

Table I. Information and features of the MMO test problems suite

	MMO test problem name	Scalable number of variables	Scalable number of objectives	Pareto optima known	Pareto front geometry	Pareto set geometry	Scalable number of Pareto set	*N*_ops (*N*_global + *N*_local)
1	MMF1	×	×	✓	Convex	Nonlinear	×	2 + 0
2	MMF2	×	×	✓	Convex	Nonlinear	×	2 + 0
3	MMF4	×	×	✓	Concave	Nonlinear	×	2 + 0
4	MMF5	×	×	✓	Convex	Nonlinear	×	2 + 0
5	MMF7	×	×	✓	Convex	Nonlinear	×	2 + 0
6	MMF8	×	×	✓	Concave	Nonlinear	×	2 + 0
7	MMF10	×	×	✓	Convex	Linear	×	1 + 0
8	MMF11	×	×	✓	Convex	Linear	✓	1 + 0
9	MMF12	×	×	✓	Convex	Linear	✓	1 + 0
10	MMF13	×	×	✓	Convex	Nonlinear	✓	1 + 0
11	MMF14	✓	✓	✓	Concave	Linear	✓	2 + 0
12	MMF15	✓	✓	✓	Concave	Linear	✓	1 + 0
13	MMF1_e	×	×	✓	Convex	Nonlinear	×	2 + 0
14	MMF14_a	✓	✓	✓	Concave	Nonlinear	✓	2 + 0
15	MMF15_a	✓	✓	✓	Concave	Nonlinear	✓	1 + 0
16	MMF10_*l*	×	×	✓	Convex	Linear	×	1 + 1
17	MMF11_*l*	×	×	✓	Convex	Linear	✓	1 + 1
18	MMF12_*l*	×	×	✓	Convex	Linear	✓	1 + 1
19	MMF13_*l*	×	×	✓	Convex	Nonlinear	✓	1 + 1
20	MMF15_*l*	✓	✓	✓	Concave	Linear	✓	1 + 1
21	MMF15_a_*l*	✓	✓	✓	Concave	Nonlinear	✓	1 + 1
22	MMF16_*l*1	✓	✓	✓	Concave	Linear	✓	2 + 1
23	MMF16_*l*2	✓	✓	✓	Concave	Linear	✓	1 + 2
24	MMF16_*l*3	✓	✓	✓	Concave	Linear	✓	2 + 2

*N*_ops represents the number of PS to be obtained. *N*_ops = *N*_global + *N*_local where *N*_global represents the number of global PS need to be obtained and *N*_local represent the number of local PSs need to be obtained.

*Please Notice: These problems should be treated as black-box problems. The explicit equations of the problems are not allowed to be used. However, the dimensionality of the problems and the total number of function evaluations can be considered as known values which can be used to design your algorithm.

1.3 Definitions of the CEC'2020 MMO test problems

The equations and figures of true PS and PF are present in this subsection.

MMF1

$$\begin{cases} f_1 = |x_1 - 2| \\ f_2 = 1 - \sqrt{|x_1 - 2|} + 2(x_2 - \sin(6\pi|x_1 - 2| + \pi))^2 \end{cases}$$

Its search space is

$$x_1 \in [1, 3], \quad x_2 \in [-1, 1].$$

Its global PSs are

$$\begin{cases} x_1 = x_1 \\ x_2 = \sin(6\pi|x_1 - 2| + \pi) \end{cases}$$

where $1 \le x_1 \le 3$.

Its global PFs are

$$f_2 = 1 - \sqrt{f_1}$$

where $0 \le f_1 \le 1$.

Its true PSs and PF are illustrated in Fig. 2.

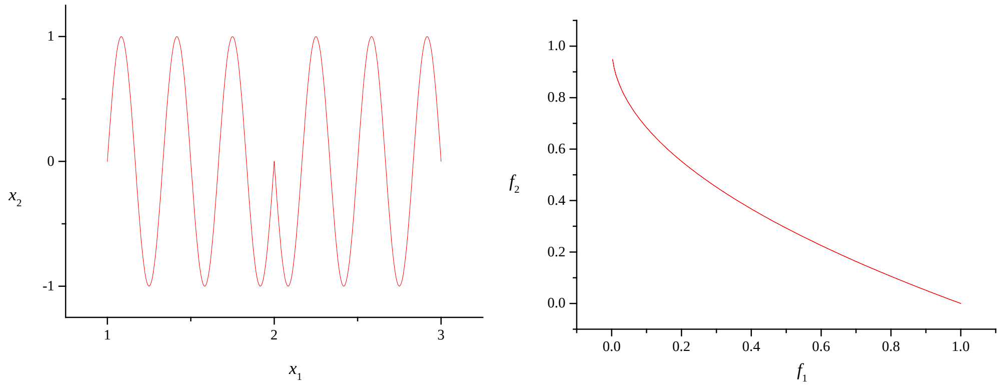

(a) True PSs of MMF1 (b) True PF of MMF1

Fig. 2. The true PSs and PF of MMF1.

MMF1_e

$$\text{Min} \begin{cases} f_1 = |x_1 - 2| \\ f_2 = \begin{cases} 1 - \sqrt{|x_1 - 2|} + 2(x_2 - \sin(6\pi|x_1 - 2| + \pi))^2, \; x_1 \in [1, 2) \\ 1 - \sqrt{|x_1 - 2|} + 2(x_2 - a^{x_1}\sin(6\pi|x_1 - 2| + \pi))^2, \; x_1 \in [2, 3] \end{cases} \end{cases}$$

where $a > 0 \,\&\, a \ne 1$ (a controls the amplitude of the global PS in $x_1 \in [2, 3]$).

Its search space is

$$x_1 \in [1, 3], \; x_2 \in [-a^3, a^3].$$

Its global PSs are

$$x_2 = \begin{cases} \sin(6\pi|x_1 - 2| + \pi), \; x_1 \in [1, 2) \\ a^{x_1}\sin(2\pi|x_1 - 2| + \pi), \; x_1 \in [2, 3] \end{cases}$$

where $a>0 \,\&\, a\neq 1$.

Its global PF is

$$f_2 = 1-\sqrt{f_1},\ f_1\in[0,1]$$

When $a=e$, its true PSs and PF are shown in Fig. 3.

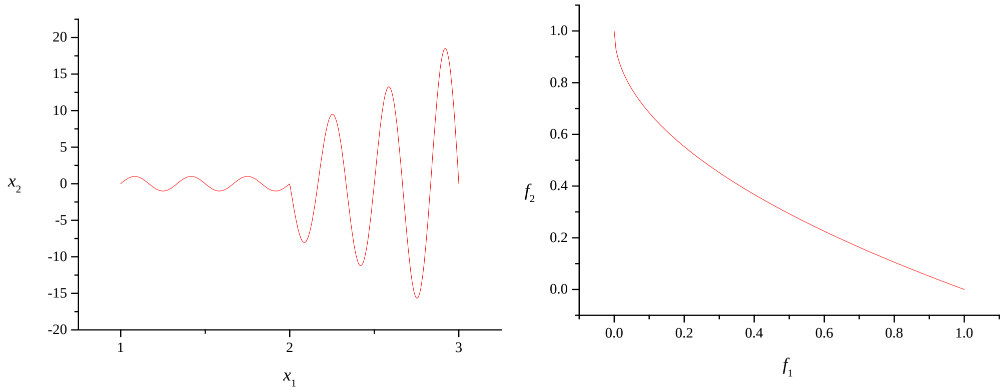

(a) True PSs of MMF1_e (b) True PF of MMF1_e

Fig. 3. The true PSs and PF of MMF1_e.

MMF2

$$\begin{cases} f_1 = x_1 \\ f_2 = \begin{cases} 1-\sqrt{x_1}+2(4(x_2-\sqrt{x_1})^2 \\ -2\cos(\dfrac{20(x_2-\sqrt{x_1})\pi}{\sqrt{2}})+2), \quad 0\le x_2\le 1 \\ 1-\sqrt{x_1}+2(4(x_2-1-\sqrt{x_1})^2 \\ -\cos(\dfrac{20(x_2-1-\sqrt{x_1})\pi}{\sqrt{2}})+2), 1< x_2\le 2 \end{cases} \end{cases}$$

Its search space is

$$x_1\in[0,1],\quad x_2\in[0,2].$$

Its global PSs are

$$\begin{cases} x_2 = x_2 \\ x_1 = \begin{cases} x_2^2 & 0\le x_2\le 1 \\ (x_2-1)^2 & 1< x_2\le 2 \end{cases} \end{cases}$$

Its global PFs are

$$f_2 = 1-\sqrt{f_1}$$

where $0\le f_1\le 1$.

Its true PSs and PF are illustrated in Fig. 4.

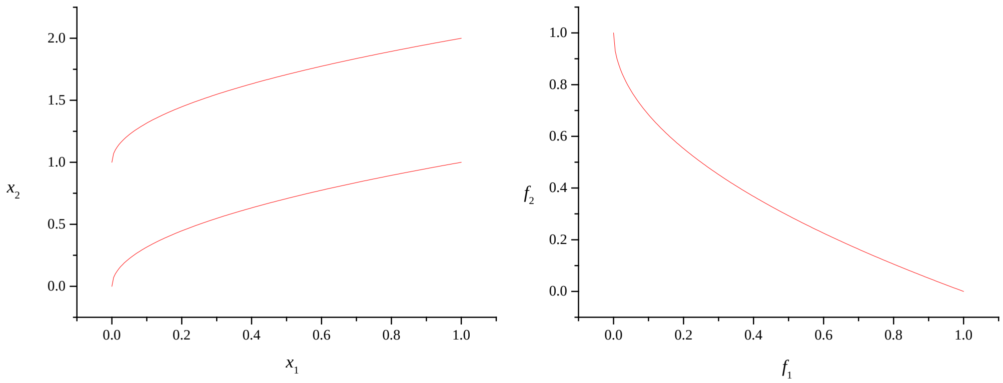

(a) True PSs of MMF2 (b) True PF of MMF2

Fig. 4. The true PSs and PF of MMF2.

MMF4

$$\begin{cases} f_1 = |x_1| \\ f_2 = \begin{cases} 1 - x_1^{\ 2} + 2(x_2 - \sin(\pi|x_1|))^2 & 0 \le x_2 < 1 \\ 1 - x_1^{\ 2} + 2(x_2 - 1 - \sin(\pi|x_1|))^2 & 1 \le x_2 \le 2 \end{cases} \end{cases}$$

Its search space is

$$x_1 \in [-1, 1], \quad x_2 \in [0, 2].$$

Its global PSs are

$$\begin{cases} x_1 = x_1 \\ x_2 = \begin{cases} \sin(\pi|x_1|) & 0 \le x_2 \le 1 \\ \sin(\pi|x_1|) + 1 & 1 < x_2 \le 2 \end{cases} \end{cases}$$

Its global PFs are

$$f_2 = 1 - f_1^{\ 2}$$

where $0 \le f_1 \le 1$.

Its true PSs and PF are illustrated in Fig. 5.

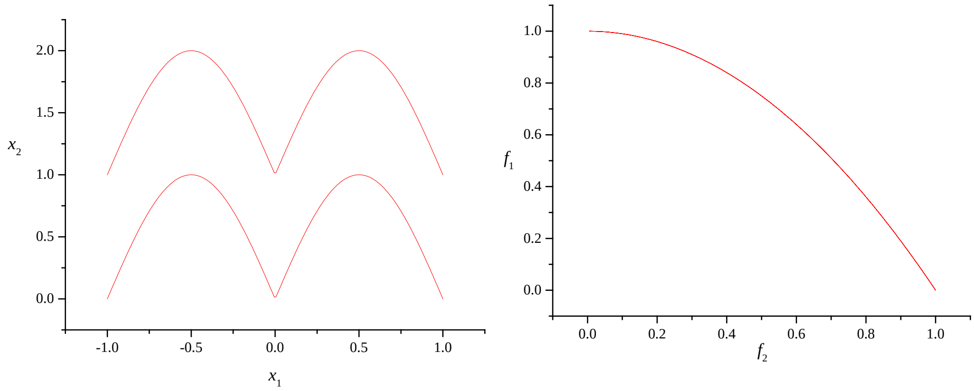

(a) True PSs of MMF4 (b) True PF of MMF4

Fig. 5. The true PSs and PF of MMF4.

MMF5

$$\begin{cases} f_1 = |x_1 - 2| \\ f_2 = \begin{cases} 1 - \sqrt{|x_1 - 2|} + 2(x_2 - \sin(6\pi|x_1 - 2| + \pi))^2 & -1 \le x_2 \le 1 \\ 1 - \sqrt{|x_1 - 2|} + 2(x_2 - 2 - \sin(6\pi|x_1 - 2| + \pi))^2 & 1 < x_2 \le 3 \end{cases} \end{cases}$$

Its search space is

$$x_1 \in [-1, 3], \quad x_2 \in [1, 3].$$

Its global PSs are

$$x_2 = \begin{cases} \sin(6\pi\,|\,x_1 - 2\,|+\pi) & -1 \le x_2 \le 1 \\ \sin(6\pi\,|\,x_1 - 2\,|+\pi) + 2 & 1 < x_2 \le 3 \end{cases}$$

Its global PFs are

$$f_2 = 1 - \sqrt{f_1}$$

where $0 \le f_1 \le 1$.

Its true PSs and PF are illustrated in Fig. 6.

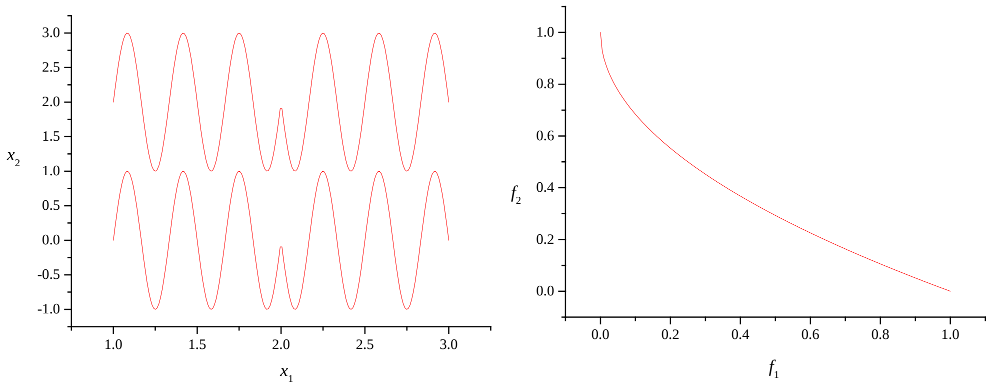

(a) True PSs of MMF5 (b) True PF of MMF5

Fig. 6. The true PSs and PF of MMF5.

MMF7

$$\begin{cases} f_1 = |x_1 - 2| \\ f_2 = 1 - \sqrt{|x_1 - 2|} + \{x_2 - [0.3|x_1 - 2|^2 \cdot \cos(24\pi|x_1 - 2| \\ \quad +4\pi) + 0.6|x_1 - 2|] \cdot \sin(6\pi|x_1 - 2| + \pi)\}^2 \end{cases}$$

Its search space is

$$x_1 \in [1, 3], \quad x_2 \in [-1, 1].$$

Its global PSs are

$$x_2 = [0.3|x_1 - 2|^2 \cos(24\pi|x_1 - 2| + 4\pi) + 0.6|x_1 - 2|] \cdot \sin(6\pi|x_1 - 2| + \pi)$$

where $1 \le x_1 \le 3$.

Its global PFs are

$$f_2 = 1 - \sqrt{f_1}$$

where $0 \le f_1 \le 1$.

Its true PSs and PF are illustrated in Fig. 7.

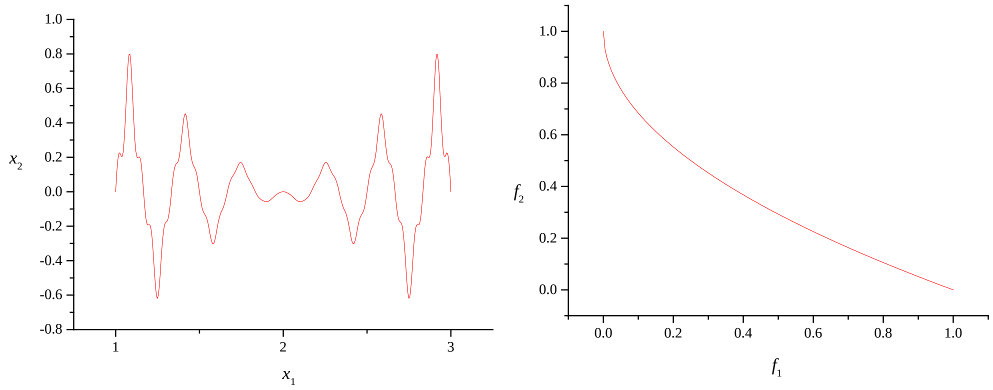

(a) True PSs of MMF7 (b) True PF of MMF7

Fig. 7. The true PSs and PF of MMF7.

MMF8

$$\begin{cases} f_1 = \sin|x_1| \\ f_2 = \begin{cases} \sqrt{1-(\sin|x_1|)^2} + 2(x_2 - \sin|x_1| - |x_1|)^2 & 0 \le x_2 \le 4 \\ \sqrt{1-(\sin|x_1|)^2} + 2(x_2 - 4 - \sin|x_1| - |x_1|)^2 & 4 < x_2 \le 9 \end{cases} \end{cases}$$

Its search space is

$$x_1 \in [-\pi, \pi], \quad x_2 \in [0, 9].$$

Its global PSs are

$$x_2 = \begin{cases} \sin|x_1| + |x_1| & 0 \le x_2 \le 4 \\ \sin|x_1| + |x_1| + 4 & 4 < x_2 \le 9 \end{cases}$$

where $-\pi \le x_1 \le \pi$.

Its global PFs are

$$f_2 = 1 - \sqrt{f_1}$$

where $0 \le f_1 \le 1$.

Its true PSs and PF are illustrated in Fig. 8.

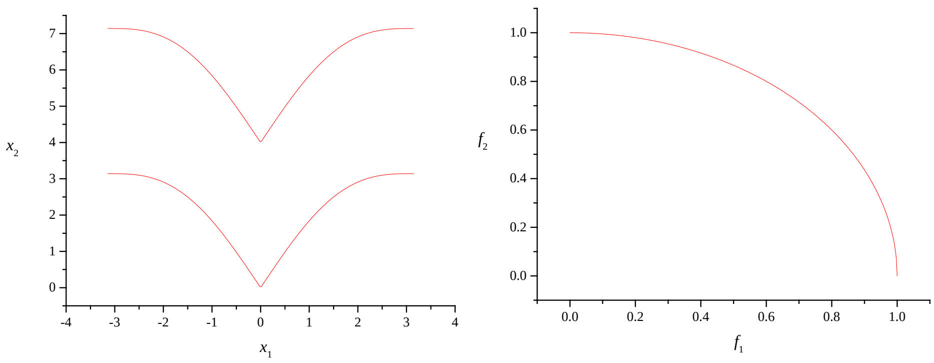

(a) True PSs of MMF8 (b) True PF of MMF8

Fig. 8. The true PSs and PF of MMF8.

MMF10 / MMF10_*l*

$$\text{Min} \begin{cases} f_1 = x_1 \\ f_2 = \dfrac{g(x_2)}{x_1} \end{cases}$$

where $g(x) = 2 - \exp\left[-\left(\dfrac{x-0.2}{0.004}\right)^2\right] - 0.8\exp\left[-\left(\dfrac{x-0.6}{0.4}\right)^2\right]$.

Its search space is

$$x_1 \in [0.1, 1.1],\ \ x_2 \in [0.1, 1.1].$$

Its global PS is

$$x_2 = 0.2,\ x_1 \in [0.1, 1.1].$$

Its local PS is

$$x_2 = 0.6,\ x_1 \in [0.1, 1.1].$$

Its global PF is

$$f_2 = \frac{g(0.2)}{f1},\ f_1 \in [0.1, 1.1].$$

Its local PF is

$$f_2 = \frac{g(0.6)}{f1},\ f_1 \in [0.1, 1.1].$$

Its true PSs and PFs are shown in Fig. 9.

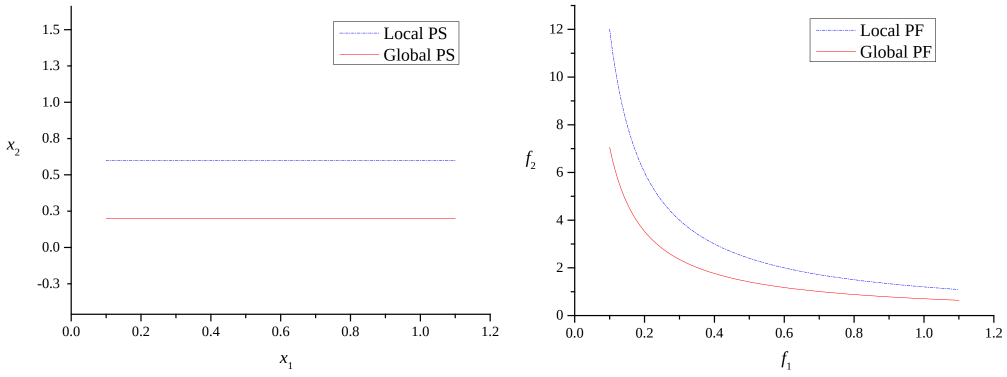

(a) True PSs of MMF10 (b) True PFs of MMF10

Fig. 9. The true PSs and PFs of MMF10.

MMF11 / MMF11_*l*

$$\text{Min} \begin{cases} f_1 = x_1 \\ f_2 = \dfrac{g(x_2)}{x_1} \end{cases}$$

where $g(x)=2-\exp\left[-2\log(2)\cdot\left(\frac{x-0.1}{0.8}\right)^2\right]\cdot\sin^6\left(n_p\pi x\right)$, n_p is the total number of global and local PSs.

Its search space is

$$x_1\in[0.1,1.1],\ \ x_2\in[0.1,1.1].$$

Its global PS is

$$x_2=\frac{1}{2n_p},\ x_1\in[0.1,1.1].$$

Its ith local PS is

$$x_2=\frac{1}{2n_p}+\frac{1}{n_p}\cdot(i-1),\ x_1\in[0.1,1.1]$$

where $i=2,3,\ldots,n_p$.

Its global PF is

$$f_2=\frac{g\left(\frac{1}{2n_p}\right)}{f_1},\ f_1\in[0.1,1.1].$$

Its local PF is

$$f_2=\frac{g\left(\frac{1}{2n_p}+\frac{1}{n_p}\cdot(i-1)\right)}{f_1},\ f_1\in[0.1,1.1]$$

where $i=2,3,\ldots,n_p$.

When $n_p=2$, its true PSs and PFs are shown in Fig. 10.

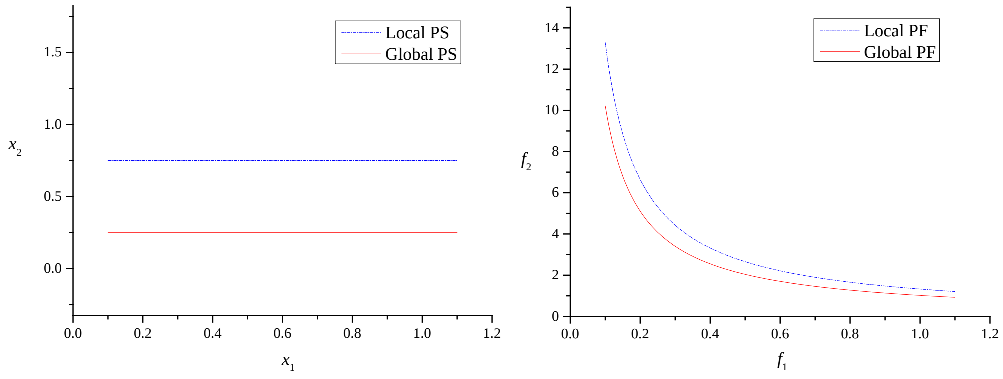

(a) True PSs of MMF11 (b) True PFs of MMF11

Fig. 10. The true PSs and PFs of MMF11.

MMF12 / MMF12_*l*

$$\text{Min} \begin{cases} f_1 = x_1 \\ f_2 = g(x_2) \cdot h(f_1, g) \end{cases}$$

where $g(x) = 2 - \exp\left[-2\log(2)\cdot\left(\frac{x-0.1}{0.8}\right)^2\right]\cdot\sin^6\left(n_p\pi x\right)$, n_p is the total number of global and local PSs, $h(f_1, g) = 1 - \left(\frac{f_1}{g}\right)^2 - \frac{f_1}{g}\sin(2\pi q f_1)$, q is the number of discontinuous pieces in each PF (PS).

Its search space is

$$x_1 \in [0,1], \quad x_2 \in [0,1].$$

Its global PS is discontinuous pieces in

$$x_2 = \frac{1}{2n_p}.$$

Its ith local PSs are discontinuous pieces in

$$x_2 = \frac{1}{2n_p} + \frac{1}{n_p}\cdot(i-1)$$

where $i = 2, 3, \ldots, n_p$.

Its global PF is discontinuous pieces in

$$f_2 = g^* \cdot h(f_1, g^*)$$

where g^* is the global optimum of $g(x)$.

Its local PFs are discontinuous pieces in

$$f_2 = g_l^* \cdot h(f_1, g_l^*)$$

where g_l^* are the local optima of $g(x)$.

The ranges of discontinuous pieces depend on the minima of $f_2 = g^* \cdot h(f_1, g^*)$.

When $n_p = 2$, its true PSs and PFs are shown in Fig. 11.

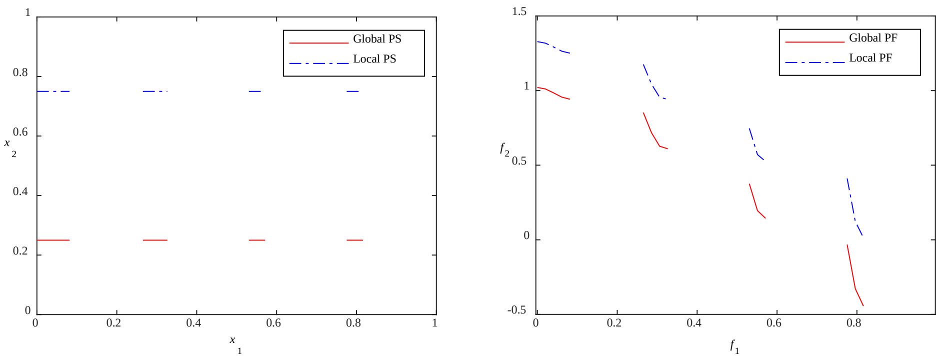

(a) True PSs of MMF12 (b) True PFs of MMF12

Fig. 11. The true PSs and PFs of MMF12.

MMF13 / MMF13_*l*

$$\min \begin{cases} f_1 = x_1 \\ f_2 = \dfrac{g(t)}{x_1} \end{cases}$$

where $g(t) = 2 - \exp\left[-2\log(2)\cdot\left(\dfrac{t-0.1}{0.8}\right)^2\right]\cdot\sin^6\left(n_p\pi(t)\right)$,

$t = x_2 + \sqrt{x_3}$, n_p is the total number of global and local PSs.

Its search space is

$$x_1 \in [0.1, 1.1],\ x_2 \in [0.1, 1.1],\ x_3 \in [0.1, 1.1].$$

Its global PS is

$$x_2 + \sqrt{x_3} = \frac{1}{2n_p},\ x_1 \in [0.1, 1.1].$$

Its ith local PSs is

$$x_2 + \sqrt{x_3} = \frac{1}{2n_p} + \frac{i-1}{n_p},\ x_1 \in [0.1, 1.1].$$

where $i = 2, 3, \ldots, n_p$.

Its global PF is

$$f_2 = \frac{2 - \exp\left[-2\log(2)\cdot\left(\dfrac{\frac{1}{2n_p} - 0.1}{0.8}\right)^2\right]\cdot\sin^6\left(n_p\pi\left(\dfrac{1}{2n_p}\right)\right)}{f_1}$$

Its local PFs are

$$f_2 = \frac{2-\exp\left[-2\log(2)\cdot\left(\frac{\left(\frac{1}{2n_p}+\frac{i-1}{n_p}\right)-0.1}{0.8}\right)^2\right]\cdot\sin^6\left(n_p\pi\left(\frac{1}{2n_p}+\frac{i-1}{n_p}\right)\right)}{f_1}$$

where $i = 2, 3, \ldots, n_p$.

When $n_p = 2$, its true PSs and PFs are shown in Fig. 12.

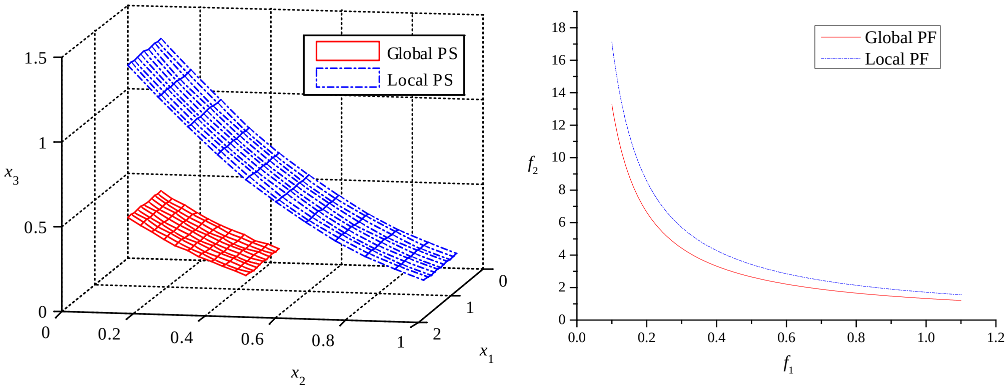

(a) True PSs of MMF13 (b) True PFs of MMF13

Fig. 12. The true PSs and PFs of MMF13.

MMF14

$$\text{Min}\begin{cases} f_1 = \cos(\pi/2\, x_1)\cos(\pi/2\, x_2)\cdots\cos(\pi/2\, x_{m-2})\cos(\pi/2\, x_{m-1})(1+g(x_m, x_{m+1}, \ldots, x_{m-1+k})) \\ f_2 = \cos(\pi/2\, x_1)\cos(\pi/2\, x_2)\cdots\cos(\pi/2\, x_{m-2})\sin(\pi/2\, x_{m-1})(1+g(x_m, x_{m+1}, \ldots, x_{m-1+k})) \\ f_3 = \cos(\pi/2\, x_1)\cos(\pi/2\, x_2)\cdots\sin(\pi/2\, x_{m-2})(1+g(x_m, x_{m+1}, \ldots, x_{m-1+k})) \\ \vdots \\ f_{m-1} = \cos(\pi/2\, x_1)\sin(\pi/2\, x_2)(1+g(x_m, x_{m+1}, \ldots, x_{m-1+k})) \\ f_m = \sin(\pi/2\, x_1)(1+g(x_m, x_{m+1}, \ldots, x_{m-1+k})) \end{cases}$$

where $g(x_m, x_{m+1}, \ldots, x_{m-1+k}) = 2-\sin^2\left(n_p\pi\left(x_{m-1+k}\right)\right)$, n_p is the number of global PSs.

Its search space is

$$x_i \in [0,1], \text{ for } i = 1, 2, \ldots, n,$$

where n is the dimension of decision space; m is the dimension of objective space; $k = n-(m-1)$.

Its ith (i = 1, 2,…, n_p) global PSs are

$$x_n = \frac{1}{2n_p} + \frac{1}{n_p}\cdot(i-1),\ x_j \in [0,1] \text{ for } j = 1:n-1.$$

Its global PFs are

$$\sum_{j=1}^{M}\left(f_j\right)^2 = (1+g^*)^2$$

where g^* are the global optima of $g(x)$.

When $n_p = 2, m = 2, n = 3$, its true PSs and PFs are shown in Fig. 13.

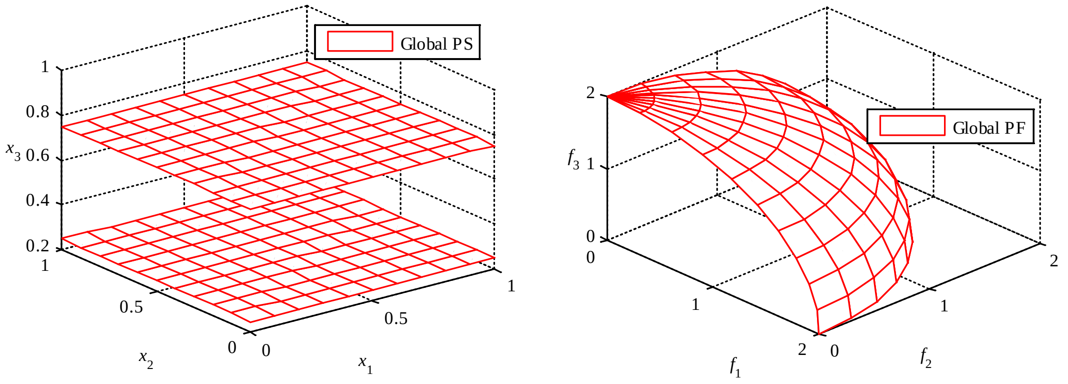

(a) True PSs of MMF14 (b) True PFs of MMF14

Fig. 13. The true PSs and PFs of MMF14.

MMF14_a

$$\text{Min}\begin{cases} f_1 = \cos(\pi/2\, x_1)\cos(\pi/2\, x_2)\cdots\cos(\pi/2\, x_{m-2})\cos(\pi/2\, x_{m-1})(1+g(x_m, x_{m+1}, \dots, x_{m-1+k})) \\ f_2 = \cos(\pi/2\, x_1)\cos(\pi/2\, x_2)\cdots\cos(\pi/2\, x_{m-2})\sin(\pi/2\, x_{m-1})(1+g(x_m, x_{m+1}, \dots, x_{m-1+k})) \\ f_3 = \cos(\pi/2\, x_1)\cos(\pi/2\, x_2)\cdots\sin(\pi/2\, x_{m-2})(1+g(x_m, x_{m+1}, \dots, x_{m-1+k})) \\ \vdots \\ f_{m-1} = \cos(\pi/2\, x_1)\sin(\pi/2\, x_2)(1+g(x_m, x_{m+1}, \dots, x_{m-1+k})) \\ f_m = \sin(\pi/2\, x_1)(1+g(x_m, x_{m+1}, \dots, x_{m-1+k})) \end{cases}$$

where $g(x_m, x_{m+1}, \dots, x_{m-1+k}) = 2 - \sin^2\left(n_p\pi\left(x_{m-1+k} - 0.5\sin\left(\pi x_{m-2+k}\right) + \frac{1}{2n_p}\right)\right)$, n_p is the number of global PSs.

Its search space is

$$x_i \in [0, 1], \text{ for } i = 1, 2, \dots, n$$

where n is the dimension of decision space; m is the dimension of objective space $k = n-(m-1)$.

Its ith (i = 1, 2,…, n_p) global PSs are

$$x_n = 0.5\sin\left(\pi x_{n-1}\right) + \frac{1}{n_p}\cdot(i-1),\ x_j \in [0,1] \text{ for } j = 1 : n-1.$$

Its global PFs are

$$\sum_{j=1}^{M}\left(f_j\right)^2 = (1+g^*)^2$$

where g^* are the global optima of $g(x)$.

When $n_p = 2, m = 2, n = 3$, its true PSs and PFs are shown in Fig. 14.

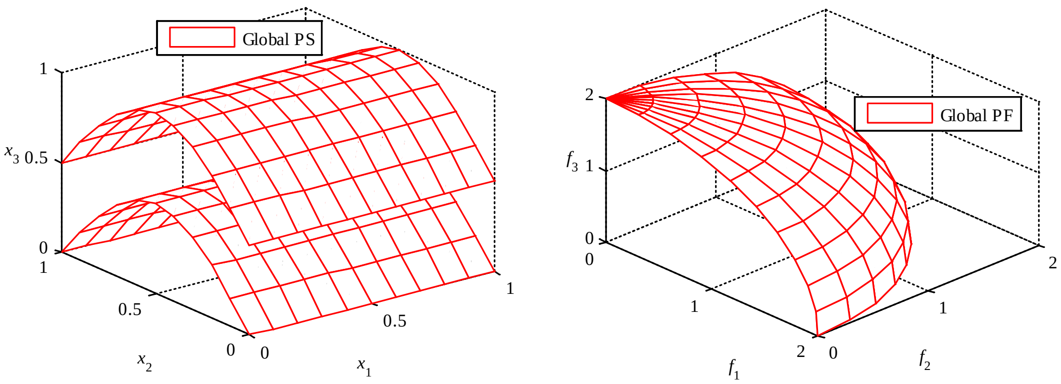

(a) True PSs of MMF14_a (b) True PFs of MMF14_a

Fig. 14. The true PSs and PFs of MMF14_a.

MMF15 / MMF15_*l*

$$\text{Min}\begin{cases} f_1 = \cos(\pi/2\, x_1)\cos(\pi/2\, x_2)\cdots\cos(\pi/2\, x_{m-2})\cos(\pi/2\, x_{m-1})\left(1+g(x_m, x_{m+1}, \ldots, x_{m-1+k})\right) \\ f_2 = \cos(\pi/2\, x_1)\cos(\pi/2\, x_2)\cdots\cos(\pi/2\, x_{m-2})\sin(\pi/2\, x_{m-1})\left(1+g(x_m, x_{m+1}, \ldots, x_{m-1+k})\right) \\ f_3 = \cos(\pi/2\, x_1)\cos(\pi/2\, x_2)\cdots\sin(\pi/2\, x_{m-2})\left(1+g(x_m, x_{m+1}, \ldots, x_{m-1+k})\right) \\ \vdots \\ f_{m-1} = \cos(\pi/2\, x_1)\sin(\pi/2\, x_2)\left(1+g(x_m, x_{m+1}, \ldots, x_{m-1+k})\right) \\ f_m = \sin(\pi/2\, x_1)\left(1+g(x_m, x_{m+1}, \ldots, x_{m-1+k})\right) \end{cases}$$

where $g(x_m, x_{m+1}, \ldots, x_{m-1+k}) = 2 - \exp\left[-2\log(2)\cdot\left(\frac{x_{m-1+k}-0.1}{0.8}\right)^2\right]\cdot\sin^2\left(n_p \pi x_{m-1+k}\right)$, n_p is the number of global PSs.

Its search space is

$$x_i \in [0,1], \text{ for } i = 1, 2, \ldots, n,$$

where n is the dimension of decision space; m is the dimension of objective space; $k = n-(m-1)$.

Its global PS is

$$x_n = \frac{1}{2n_p}, x_j \in [0,1] \text{ for } j = 1:n-1.$$

Its ith (i = 2, 3,…, n_p) local PSs are

$$x_n = \frac{1}{2n_p} + \frac{1}{n_p}\cdot(i-1), x_j \in [0,1] \text{ for } j = 1:n-1.$$

Its global PF is

$$\sum_{j=1}^{M}\left(f_j\right)^2 = (1+g^*)^2$$

where g^* is the global optimum of $g(x)$.

Its ith local PFs are

$$\sum_{j=1}^{M}\left(f_j\right)^2 = (1+g_l^*)^2$$

where g_l^* are the local optima of $g(x)$.

When $n_p = 2, m = 2, n = 3$, its true PSs and PFs are shown in Fig. 15.

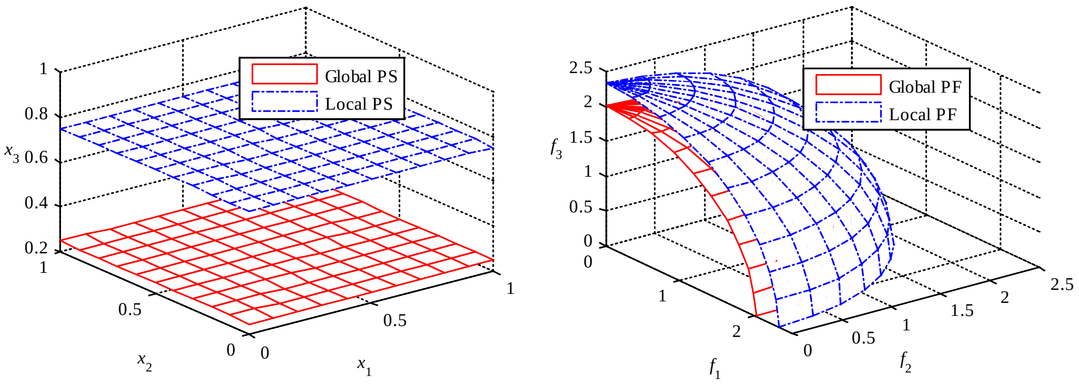

(a) True PSs of MMF15 (b) True PFs of MMF15

Fig. 15. The true PSs and PFs of MMF15.

MMF15_a / MMF15_a_*l*

$$\text{Min}\begin{cases} f_1 = \cos(\tfrac{\pi}{2}x_1)\cos(\tfrac{\pi}{2}x_2)\cdots\cos(\tfrac{\pi}{2}x_{m-2})\cos(\tfrac{\pi}{2}x_{m-1})\left(1+g(x_m, x_{m+1}, \ldots, x_{m-1+k})\right) \\ f_2 = \cos(\tfrac{\pi}{2}x_1)\cos(\tfrac{\pi}{2}x_2)\cdots\cos(\tfrac{\pi}{2}x_{m-2})\sin(\tfrac{\pi}{2}x_{m-1})\left(1+g(x_m, x_{m+1}, \ldots, x_{m-1+k})\right) \\ f_3 = \cos(\tfrac{\pi}{2}x_1)\cos(\tfrac{\pi}{2}x_2)\cdots\sin(\tfrac{\pi}{2}x_{m-2})\left(1+g(x_m, x_{m+1}, \ldots, x_{m-1+k})\right) \\ \vdots \\ f_{m-1} = \cos(\tfrac{\pi}{2}x_1)\sin(\tfrac{\pi}{2}x_2)\left(1+g(x_m, x_{m+1}, \ldots, x_{m-1+k})\right) \\ f_m = \sin(\tfrac{\pi}{2}x_1)\left(1+g(x_m, x_{m+1}, \ldots, x_{m-1+k})\right) \end{cases}$$

where $g(x_m, x_{m+1}, \ldots, x_{m-1+k}) = 2 - \exp\left[-2\log(2)\cdot\left(\frac{t-0.1}{0.8}\right)^2\right]\cdot\sin^2\left(n_p\pi t\right)$,

$t = x_{m-1+k} - 0.5\sin\left(\pi x_{m-2+k}\right) + \frac{1}{2n_p}$, n_p is the number of global PSs.

Its search space is

$$x_i \in [0,1], \text{ for } i = 1, 2, \ldots, n$$

where n is the dimension of decision space; m is the dimension of objective space; $k = n - (m-1)$.

Its global PS is

$$x_n = 0.5\sin\left(\pi x_{n-1}\right), \; x_j \in [0,1] \text{ for } j = 1 : n-1.$$

Its ith (i = 2, 3,…, n_p) local PSs are

$$x_n = 0.5\sin\left(\pi x_{n-1}\right) + \frac{1}{n_p}(i-1), \; x_j \in [0,1] \text{ for } j = 1 : n-1.$$

Its global PF is

$$\sum_{j=1}^{M}\left(f_j\right)^2 = (1+g^*)^2$$

where g^* is the global optimum of $g(x)$.

Its ith local PFs are

$$\sum_{j=1}^{M}\left(f_j\right)^2=(1+g_l^*)^2$$

where g_l^* are the local optima of $g(x)$.

When $n_p=2, m=2, n=3$, its true PSs and PFs are shown in Fig. 16.

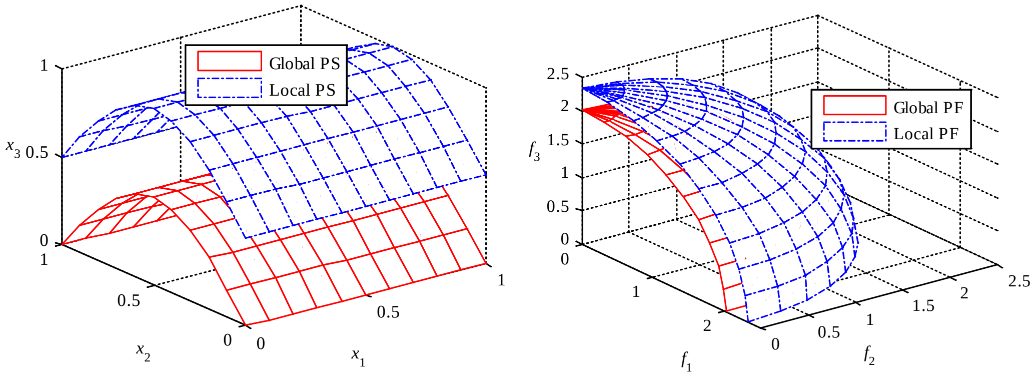

(a) True PSs of MMF15_a (b) True PFs of MMF15_a

Fig. 16. The true PSs and PFs of MMF15_a.

MMF16

$$\text{Min}\begin{cases}f_1=\cos(\pi/2\,x_1)\cos(\pi/2\,x_2)\cdots\cos(\pi/2\,x_{m-2})\cos(\pi/2\,x_{m-1})(1+g(x_m,x_{m+1},\ldots,x_{m-1+k}))\\ f_2=\cos(\pi/2\,x_1)\cos(\pi/2\,x_2)\cdots\cos(\pi/2\,x_{m-2})\sin(\pi/2\,x_{m-1})(1+g(x_m,x_{m+1},\ldots,x_{m-1+k}))\\ f_3=\cos(\pi/2\,x_1)\cos(\pi/2\,x_2)\cdots\sin(\pi/2\,x_{m-2})(1+g(x_m,x_{m+1},\ldots,x_{m-1+k}))\\ \vdots\\ f_{m-1}=\cos(\pi/2\,x_1)\sin(\pi/2\,x_2)(1+g(x_m,x_{m+1},\ldots,x_{m-1+k}))\\ f_m=\sin(\pi/2\,x_1)(1+g(x_m,x_{m+1},\ldots,x_{m-1+k}))\end{cases}$$

where

$$g(x_m,x_{m+1},\ldots,x_{m-1+k})=\begin{cases}2-\sin^2\left(2n_{p\_g}\pi\left(x_{m-1+k}\right)\right) & 0\le x_{m-1+k}<0.5\\ 2-\exp\left[-2\log(2)\cdot\left(\dfrac{x_{m-1+k}-0.1}{0.8}\right)^2\right]\cdot\sin^2\left(2n_{p\_l}\pi x_{m-1+k}\right) & 0.5\le x_{m-1+k}\le 1\end{cases},$$

n_{p_g} is the number of global PSs and n_{p_l} is the number of local PSs.

Its search space is

$$x_i\in[0,1],\ \text{for}\ i=1,2,\ldots,n,$$

where n is the dimension of decision space; m is the dimension of objective space; $k=n-(m-1)$.

Its ith (i = 1, 2 … $\frac{n_p}{2}$) global PS is

$$x_n = \frac{1}{2n_p} + \frac{1}{n_p} \cdot (i-1),\ x_j \in [0, 0.5)\ \text{for}\ j = 1 : n-1$$

Its ith (i = 1, 2 … $\frac{n_p}{2}$) local PSs are

$$x_n = \frac{1}{2n_p} + \frac{1}{n_p} \cdot (i-1),\ x_j \in [0.5, 1]\ \text{for}\ j = 1 : n-1.$$

Its global PFs are

$$\sum_{j=1}^{M} \left(f_j \right)^2 = (1 + g^*)^2$$

where g^* are the global optima of $g(x)$.

Its local PFs are

$$\sum_{j=1}^{M} \left(f_j \right)^2 = (1 + g_l^*)^2$$

where g_l^* are the local optima of $g(x)$.

When $n_p = 4, m = 2, n = 3$, its true PSs and PFs are shown in

MMF16_*l*1

Test function is the same with MMF16. The parameters are set as:

$n_{p\_g} = 2, n_{p\_l} = 1, m = 2, n = 3$,

Its true PSs and PFs are shown in Fig. 17.

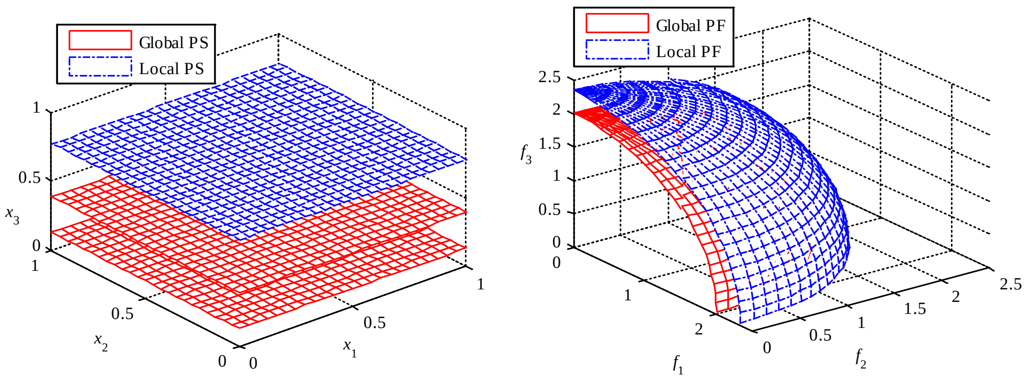

(a) True PSs of MMF16_*l*1 (b) True PFs of MMF16_*l*1

Fig. 17. The true PSs and PFs of MMF16_*l*1.

MMF16_*l*2

Test function is the same with MMF16. The parameters are set as:

$n_{p\_g} = 1, n_{p\_l} = 2, m = 2, n = 3$,

Its true PSs and PFs are shown in Fig. 18.

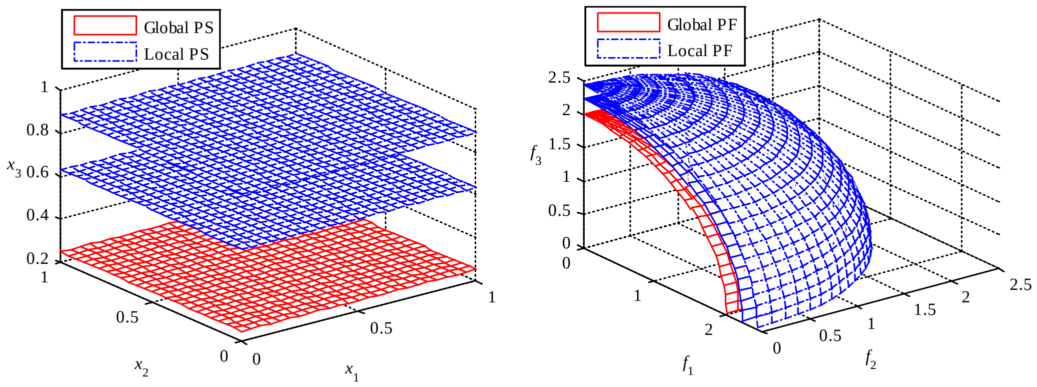

(a) True PSs of MMF16_*l*2 (b) True PFs of MMF16_*l*2

Fig. 18. The true PSs and PFs of MMF16_*l*2.

MMF16_*l*3

Test function is the same with MMF16. The parameters are set as:

$$n_{p\_g} = 2, n_{p\_l} = 2, m = 2, n = 3,$$

Its true PSs and PFs are shown in Fig. 19.

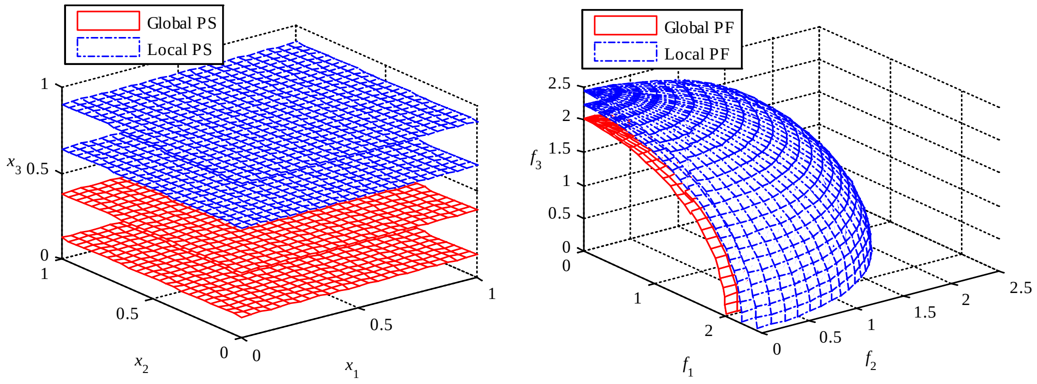

(a) True PSs of MMF16_*l*3 (b) True PFs of MMF16_*l*3

Fig. 19. The true PSs and PFs of MMF16_*l*3.

2 Evaluation criteria

2.1 Performance indicators

Four performance indicators, the reciprocal of Pareto Sets Proximity (1/*PSP*) [3], Inverted Generational Distance (*IGD* [12]) in decision space (*IGDX*) [13], the reciprocal of Hypervolume (1/*HV*) [14], and *IGD* in objective space (*IGDF*) [13] are employed to compare the performances of different algorithms. Among the indicators, 1/*PSP* and *IGDX* are used to compare the performance in decision space, while 1/*HV* and *IGDF* are used to compare the performance in objective space. The reference data including reference PFs, PSs and reference points of *HV* are available on http://www5.zzu.edu.cn/ecilab/info/1036/1163.htm. For all the four indicators, the smaller value means the better performance.

2.2 **Performance comparison method**

Algorithms are ranked through the Friedman of *k* algorithms over *N* case problems. Each algorithm is assigned four ranks according to the four indicators (1/*PSP*, *IGDX*, 1/*HV* and *IGDF*). The mean value of the four ranks acts as the final score. The algorithm with the smallest score ranks first.

2.3 **Experimental setting**

Running times: 21 times

Population size: 200**N*_ops

Maximal fitness evaluations (MaxFES): 10000 * *N*_ops

where *N_var* represents the number of variables and *N*_ops means the number of local and global PS to be obtained.

3 Results Format

Provide the best, worst, mean, median, and standard deviation values of each indicator value for the 21 runs.

The participants are required to send the final results as the following format to the organizers and the organizers will present an overall analysis and comparison based on these results.

Create one txt document with the name “AlgorithmName _IndicatorName.txt” for each indicator.

For example, the reciprocal of *PSP* of MO_Ring_PSO_SCD for test function MMF1, the file name should be “MO_Ring_PSO_SCD _rPSP.txt”.

Then save the results matrix (the gray shadowing part 24*26) as Table II-Table V in the file:

Table II. Information matrix for 1/*PSP*

***.txt	Run1	Run2	…	Run21	Best	Worst	Mean	Median	Standard deviation
MMF1									
MMF2									
MMF4									
MMF5									
…									
MMF16_*l*3									

Table III. Information matrix for 1/*HV*

***.txt	Run 1	Run2	…	Run21	Best	Worst	Mean	Median	Standard deviation
MMF1									
MMF2									
MMF4									
MMF5									
…									
MMF16_*l*3									

Table IV. Information matrix for *IGDX*

***.txt	Run1	Run2	…	Run21	Best	Worst	Mean	Median	Standard deviation

MMF1									
MMF2									
MMF4									
MMF5									
…									
MMF16_*l*3									

Table V. Information matrix for *IGDF*

***.txt	Run1	Run2	…	Run21	Best	Worst	Mean	Median	Standard deviation
MMF1									
MMF2									
MMF4									
MMF5									
…									
MMF16_*l*3									

References

[1] K. Deb, "Multi-Objective Genetic Algorithms: Problem Difficulties and Construction of Test Problems," *Evolutionary Computation*, vol. 7, no. 3, pp. 205-230, 1999.

[2] J. J. Liang, C. T. Yue, and B. Y. Qu, "Multimodal multi-objective optimization: A preliminary study," in *IEEE Congress on Evolutionary Computation*, Vancouver, 2016, pp. 2454-2461: IEEE, 2016.

[3] C. T. Yue, B. Y. Qu, and J. J. Liang, "A multiobjective particle swarm optimizer using ring topology for solving multimodal multiobjective problems," *IEEE Transactions on Evolutionary Computation*, vol. 22, no. 5, pp. 805-817, 2018.

[4] J. J. Liang, Q. Q. Guo, C. T. Yue, B. Y. Qu, and K. J. Yu, "A Self-organizing Multi-objective Particle Swarm Optimization Algorithm for Multimodal Multi-objective Problems," in *International Conference on Swarm Intelligence*, 2018, pp. 550-560: Springer.

[5] Y. Liu, G. G. Yen, and D. Gong, "A Multi-Modal Multi-Objective Evolutionary Algorithm Using Two-Archive and Recombination Strategies," *IEEE Transactions on Evolutionary Computation*, vol. 23, no. 4, pp. 660-674, 2018.

[6] R. Tanabe and H. Ishibuchi, "A Decomposition-Based Evolutionary Algorithm for Multi-modal Multi-objective Optimization," in *International Conference on Parallel Problem Solving from Nature*, Coimbra, 2018, pp. 249-261: Springer.

[7] T. Tušar and B. Filipič, "Visualization of Pareto front approximations in evolutionary multiobjective optimization: A critical review and the prosection method," *IEEE Transactions on Evolutionary Computation*, vol. 19, no. 2, pp. 225-245, 2015.

[8] P. Kerschke and C. Grimme, "An Expedition to Multimodal Multi-objective Optimization Landscapes," in *International Conference on Evolutionary Multi-Criterion Optimization*, 2017, pp. 329-343: Springer.

[9] Y. Liu, H. Ishibuchi, and Y. Nojima, "A Double-Niched Evolutionary Algorithm and its

Behavior on Polygon-Based Problems," in *International Conference on Parallel Problem Solving from Nature*, 2018, pp. 262-273.

[10] C. T. Yue, J. J. Liang, B. Y. Qu, K. J. Yu, and H. Song, "Multimodal Multiobjective Optimization in Feature Selection," in *IEEE Congress on Evolutionary Computation*, 2019, pp. 302-309.

[11] C. T. Yue, B. Y. Qu, K. J. Yu, J. J. Liang, and X. Li, "A Novel Scalable Test Problem Suite for Multimodal Multiobjective Optimization," *Swarm and Evolutionary Computation*, vol. 48, no. 2019, pp. 62-71, 2019.

[12] Q. Zhang, A. Zhou, and Y. Jin, "RM-MEDA: A Regularity Model-Based Multiobjective Estimation of Distribution Algorithm," *IEEE Transactions on Evolutionary Computation*, vol. 12, no. 1, pp. 41-63, 2008.

[13] A. Zhou, Q. Zhang, and Y. Jin, "Approximating the Set of Pareto-Optimal Solutions in Both the Decision and Objective Spaces by an Estimation of Distribution Algorithm," *IEEE Transactions on Evolutionary Computation*, vol. 13, no. 5, pp. 1167-1189, 2009.

[14] E. Zitzler, L. Thiele, M. Laumanns, C. M. Fonseca, and V. G. D. Fonseca, "Performance assessment of multiobjective optimizers: an analysis and review," *IEEE Transactions on Evolutionary Computation*, vol. 7, no. 2, pp. 117-132, 2003.

View publication stats
